# A survey of sequences of KT-HAK-KUP transporters in green algae and basal land plants

**DOI:** 10.1016/j.dib.2018.07.011

**Published:** 2018-07-10

**Authors:** Guillermo E. Santa María, Sonia Oliferuk, Jorge I. Moriconi

**Affiliations:** Instituto Tecnológico Chascomús (INTECH), Consejo Nacional de Investigaciones Científicas y Técnicas (CONICET) and Universidad Nacional de San Martín (UNSAM), Avda Intendente Marino km 8,2, Chascomús 7130, Provincia de Buenos Aires, Argentina

**Keywords:** Algae, HAK, KUP, KT, Transporter

## Abstract

In this data article, information is provided on sequences of KT-HAK-KUP transporters from green algae and basal land plants. A data set is offered containing sequences corresponding to the chlorophyte algae *Chlamydomonas eustigma*, *Gonium pectorale and Coccomyxa subellipsoidea*, the charophyte algae *Coleochaete orbicularis* and *Klebsormidium flaccidum,* the bryophyte *Sphagnum fallax,* the marchantophyte *Marchantia polymorpha* and the gymnosperm *Pinus taeda*, which have been not formerly analyzed. In addition, an analysis of similarity scores among representatives of the clusters recognized in photosynthetic green organisms (namely, chlorophyte algae, charophyte algae, basal embryophytes and higher embryophytes) is performed as well as an analysis of membrane topology for them.

**Specifications Table**

**Table:** 

Subject area	*Plant Biology*
More specific subject area	*Plant ion transport, transporter phylogeny*
Type of data	*Protein sequences retrieved from public databases, analysis of similarity and topological models*
How data was acquired	*Retrieved from public databases and further bioinformatic analysis*
Data format	*Raw data in fasta (for sequence data set), analyzed data (Tables and Figures)*
Experimental factors	*No applicable*
Experimental features	*No applicable*
Data source location	*Chascomús, Buenos Aires, Argentina*
Data accessibility	The data are available within this article
Related article	*KT-HAK-KUP transporters in major terrestrial photosynthetic organisms: a twenty years tale*[1]

**Value of the data**

• Data presented correspond to sequences of KT-HAK-KUP transporters retrieved from public databases which have been not formerly analyzed for the organisms *Coccomyxa subellipsoidea*, *Chlamydomonas eustigma, Gonium pectorale*, *Sphagnum fallax*, *Marchantia polymorpha, Pinus taeda*, *Coleochaete orbicularis* and *Klebsormidium flaccidum* and a new sequence for *Chlamydomonas reinhardtii.* The data set is complemented with sequences already examined for *Chlamydomonas reinhardtii, Physcomitrella patients*, *Selaginella moellendorffii*, *Picea abies*, *Amborella trichopoda*, *Arabidopsis thaliana*, *Prunus persica*, *Oryza sativa* and *Zea mays.*
• These sequences can be used to build up phylogenetic trees and to perform complementary analyses based on them. In this data article, they have been used to analyze the similarity scores in pair comparisons among members of different KT-HAK-KUP clades as well as to predict their possible topology.

## 1 Data

Data introduced correspond to new KT-HAK-KUP sequences derived from public databases and the analysis performed for them. The description of the sources for all the sequences used for *Coccomyxa subellipsoidea*, *Chlamydomonas reinhardtii*, *Chlamydomonas eustigma, Gonium pectorale, Sphagnum fallax*, *Marchantia polymorpha, Pinus taeda*, *Coleochaete orbicularis* and *Klebsormidium flaccidum* is provided in Table 1, while the corresponding sequences are provided in the accompanying data set (Appendix A. Supplementary material, Table, mentioned as Table 6 in reference [1]). The data set also contains the KT-HAK-KUP sequences already posted by Nieves-Cordones et al. [2] for *Physcomitrella patients*, *Selaginella moellendorffii*, *Picea abies*, *Amborella trichopoda*, *Arabidopsis thaliana*, *Prunus persica*, *Oryza sativa* and *Zea mays* (Supplementary material Table), which have been used to construct a phylogenetic tree of KT-HAK-KUPs in green algae and land plants [1]. The data set includes only full length sequences that contain a region with homology to the first putative first transmembrane domain as well as the highly conserved GGT(A/L/I/P/S)F(L/A)A(S)L(V/I/M/A)YS(T/A) motif as determined following their alignment with MAFFT. An alignment of chlorophyte and charophyte algae sequences together with those corresponding to *Arabidopsis thaliana* is provided (Appendix A. Supplementary material, Fig. 1). Percent similarity scores among representatives of KT-HAK-KUPs corresponding to the 12 clades identified in green photosynthetic organisms were estimated (Table 2). In the same way percent similarity scores among the members identified in each green algal clade are shown (Table 3). The capacity of four topology prediction services to generate satisfactory topological models for the bacterial KUP transporter, for which the topology has been experimentally determined [3], is summarized in Appendix A. Supplementary material, Fig. 2. It was found that all the services (TOPCONS, TOPCONS-single, THMHH 2.0 and TMPred) provided an accurate agreement with the topology of KUP. Therefore, these four services were used to analyze the number and orientation of transmembrane domains in selected members of the above mentioned clades of green photosynthetic organisms. The predicted molecular weights of these representative KT-HAK-KUP transporters are given along with a prediction of the number of transmembrane domains derived from the use of the four topology prediction services (Table 4). In Table 5 a more detailed analysis of the residues involved in each transmembrane domain is provided for canonical members of the KT-HAK-KUP family of transporters, namely HvHAK1, AtHAK5, AtKUP4(TRH1) and AtKUP7.

**Table 1:** List of sequences retrieved from data-bases, indicating their accession number and source. Sequences are provided in Supplementary Table.

**Organism**	**Identification**	**Retrieved from**	**Name**	**Previous description**
*Chlamydomonas reinhardtii*	Cre17.g714200.t1.1	Phytozome	CrHAK1	He et al. [4]
*Chlamydomonas reinhardtii*	Cre17.g714450.t1.2	Phytozome	CrHAK2	He et al. [4]
*Chlamydomonas reinhardtii*	Cre17.g714150.t1.1	Phytozome	CrHAK3	He et al. [4]
*Chlamydomonas reinhardtii*	Cre04.g217350.t1.2	Phytozome	CrHAK4	He et al. [4]
*Chlamydomonas reinhardtii*	Cre04.g214657.t1.1	Phytozome	CrHAK5	None
*Chlamydomonas eustigma*	GAX79004.1	NCBI	CeuHAK1	None
*Chlamydomonas eustigma*	GAX81809.1	NCBI	CeuHAK2	None
*Chlamydomonas eustigma*	GAX85040.1	NCBI	CeuHAK3	None
*Coccomyxa subellipsoidea C-169*	46832 Primary	Phytozome	CosubHAK2	None
*Gonium pectorale*	KXZ41691.1	NCBI	GpHAK1	None
*Klebsormidium flaccidum*	kfl00150_0260_v1.1	Transcriptome shotgun assembly database	KflHAK1	None
*Klebsormidium flaccidum*	kfl00335_0050_v1.1	Transcriptome shotgun assembly database	KflHAK2	None
*Klebsormidium flaccidum*	kfl00663_0030_v1.1	Transcriptome shotgun assembly database	KflHAK3	None
*Klebsormidium flaccidum*	kfl00971_0030_v1.1	Transcriptome shotgun assembly database	KflHAK4	None
*Klebsormidium flaccidum*	kfl00236_0145_v1.1	Transcriptome shotgun assembly database	KflHAK5	None
*Coleochaete orbicularis*	GBSL01056787.1	Transcriptome shotgun assembly database	ColorbHAK1	None
*Coleochaete orbicularis*	GBSL01029532.1	Transcriptome shotgun assembly database	ColorbHAK2	None
*Coleochaete orbicularis*	GBSL01029531.1	Transcriptome shotgun assembly database	ColorbHAK3	None
*Coleochaete orbicularis*	GBSL01051607.1	Transcriptome shotgun assembly database	ColorbHAK4	None
*Coleochaete orbicularis*	GBSL01046951.1	Transcriptome shotgun assembly database	ColorbHAK5	None
*Coleochaete orbicularis*	GBSL01051393.1	Transcriptome shotgun assembly database	ColorbHAK6	None
*Coleochaete orbicularis*	GBSL01030503.1	Transcriptome shotgun assembly database	ColorbHAK7	None
*Marchantia polymorpha*	Mapoly0070s0079.1	Phytozome	MapolHAK1	None
*Marchantia polymorpha*	Mapoly0076s0088.1	Phytozome	MapolHAK2	None
*Marchantia polymorpha*	Mapoly0011s0118.1	Phytozome	MapolHAK3	None
*Marchantia polymorpha*	Mapoly0046s0054.1	Phytozome	MapolHAK4	None
*Marchantia polymorpha*	Mapoly0317s0002.1	Phytozome	MapolHAK5	None
*Marchantia polymorpha*	Mapoly0045s0071.1	Phytozome	MapolHAK6	None
*Marchantia polymorpha*	Mapoly0014s0103.1	Phytozome	MapolHAK7	None
*Sphagnum fallax*	Sphfalx0105s0022.1	Phytozome	SphfalHAK1	None
*Sphagnum fallax*	Sphfalx0153s0001.1	Phytozome	SphfalHAK2	None
*Sphagnum fallax*	Sphfalx0008s0191.1	Phytozome	SphfalHAK3	None
*Sphagnum fallax*	Sphfalx0147s0026.1	Phytozome	SphfalHAK4	None
*Sphagnum fallax*	Sphfalx0017s0013.1	Phytozome	SphfalHAK5	None
*Sphagnum fallax*	Sphfalx0076s0081.2	Phytozome	SphfalHAK6	None
*Sphagnum fallax*	Sphfalx0162s0051.1	Phytozome	SphfalHAK7	None
*Sphagnum fallax*	Sphfalx0100s0051.1	Phytozome	SphfalHAK8	None
*Sphagnum fallax*	Sphfalx0001s0210.1	Phytozome	SphfalHAK9	None
*Sphagnum fallax*	Sphfalx0045s0071.1	Phytozome	SphfalHAK10	None
*Sphagnum fallax*	Sphfalx0049s0008.1	Phytozome	SphfalHAK12	None
*Pinus Taeda*	lcl|PITA_000007335	Congenie	PtHAK1	None
*Pinus Taeda*	lcl|PITA_000007338	Congenie	PtHAK2	None
*Pinus Taeda*	lcl|PITA_000021914	Congenie	PtHAK3	None
*Pinus Taeda*	lcl|PITA_000022279	Congenie	PtHAK4	None
*Pinus Taeda*	lcl|PITA_000005025	Congenie	PtHAK5	None
*Pinus Taeda*	lcl|PITA_000022280	Congenie	PtHAK6	None
*Pinus Taeda*	lcl|PITA_000092317	Congenie	PtHAK7	None

As stated, the remaining sequences used were retrieved from Nieves-Cordones et al. [2].

**Table 2:** Percent similarity scores, as determined by pair comparisons at https://www.ebi.ac.uk/Tools/psa/emboss_needle/, among selected transporters corresponding to clusters of KT-HAK-KUPs in photosynthetic green organisms. Clusters I to VI correspond to Embryophyta while clusters VII to XII correspond to Chlorophyta and Charophyta (Klebsormidiophyceae + Coleochaetophyceae). Taken in consideration the apparent diversity, Clusters II and XII are represented by three and two transporters, respectively.

**Cluster**		I	II b	II c	II a	III	IV	V	VI	VII	VIII	IX	X	XI	XII a
	**Transporter**	AtHAK5	AtKUP1	AtKUP2	AtKUP4	AtKUP10	PpHAK13	AtKUP7	PpHAK6	ColorbHAK7	KfHAK3	KfHAK2	ColorbHAK1	KfHAK4	CrHAK1
**I**	AtHAK5														
**II b**	AtKUP1	59.6													
**II c**	AtKUP2	58.6	67												
**II a**	AtKUP4	56	64.8	65.8											
**III**	AtKUP10	61.7	63.5	62.1	58.9										
**IV**	PpHAK13	54.7	52.8	49.6	48.6	51.2									
**V**	AtKUP7	58.3	54.1	55.1	52.7	60.3	52.3								
**VI**	PpHAK6	62.1	62.4	59.5	57.4	60.5	51.8	56.1							
**VII**	ColorbHAK7	37	34.5	38	33.2	37.8	35.5	35.2	38						
**VIII**	KflHAK3	50.9	48.8	50.4	47.9	50.8	47.9	49.6	49.8	39.4					
**IX**	KflHAK2	47.8	49.1	46.4	45.1	47.8	45.4	46.1	48.2	33.7	48.5				
**X**	ColorbHAK1	53.9	51.7	49.1	49.8	49.4	45.4	45.6	49.9	33.8	45.8	44.4			
**XI**	KflHAK4	48.8	47.5	49.6	51	48.1	44.1	47.6	46.5	37.5	48.6	45.8	48.2		
**XII a**	CrHAK1	34.6	33	35.7	34.1	31.6	28.9	33.6	35.3	31.5	38	34.2	36.1	38.7	
**XII b**	CeuHAK2	37.8	36.6	37.2	36	38.2	35.1	34.5	38.8	28.2	38.3	36.5	37.1	38.2	44.3

**Table 3:** Similarity scores (as %) within green algae clusters of KT-HAK-KUP transporters, determined as described in Table 2.

**Group VIII**									
	KflHAK3	KflHAK5							
KflHAK3	–								
KflHAK5	54.7	–							
**Group IX**									
	KflHAK2	ColorbHAK4	ColorbHAK5						
KflHAK2	–								
ColorbHAK4	61.4	–							
ColorbHAK5	59.7	89.1	–						
**Group X**									
	KflHAL1	ColorbHAK1	ColorbHAK2	ColorbHAK3					
KflHAL1	–								
ColorbHAK1	51.4	–							
ColorbHAK2	58.6	49.8	–						
ColorbHAK3	57	49.6	83.6	–					
**Group XI**									
	KflHAK4	ColorbHAK6	CosubHAK2						
KflHAK4	–								
ColorbHAK6	57.5	–							
CosubHAK2	60.6	56.1	–						
**Group XII**									
	CrHAK1	CrHAK2	CrHAK3	CrHAK4	CrHAK5	CeuHAK1	CeuHAK2	CeuHAK3	GpHAK1
CrHAK1	–								
CrHAK2	96.1	–							
CrHAK3	95.8	95.3	–						
CrHAK4	92.4	92.8	91.4	–					
CrHAK5	36.8	37.8	36.7	37.2	–				
CeuHAK1	45.3	45.7	45.6	45	31.7	–			
CeuHAK2	44.3	43.5	42.7	43.5	34.8	49.8	–		
CeuHAK3	42.9	42.9	43.1	42.6	27.3	51	62	-	
GpHAK1	54	53.5	54.7	54.2	33.5	49.4	46.5	48.2	–

**Table 4:** Predicted topology for selected members of clades of KT-HAK-KUP transporters in green photosynthetic organisms. The number of residues of each protein, its predicted molecular weight (MW), the possible number of transmembrane domains (TMs), their putative orientation as predicted with four services (TOPCONS, TOPCONS-single, TMHMM 2.0 and TMPred) as well as the predicted number of amino acidic residues potentially situated between the TM II and TM III (as predicted with TMPred) are shown.

**Cluster**	**Protein**	**Residues**	**Predicted MW (kD)**		**Number TMs/ orientation**					**Residues between TMII and TMIII**
					**TOPCONS**	**TOPCONS-single**	**TMHMM 2.0**	**TMPred**		**(TMPred)**
I	AtHAK5	785	87.86		i12i	i12i	o12o	i13o		67
II a	AtKUP4	775	86.85		i14i	i12i	i14i	i14i		73
II b	AtKUP2	794	88.64		i14i	i12i	i12i	i14i		66
II c	AtKUP1	712	79.14		i14i	i12i	i13o	i13o		71
III	AtKUP10	796	89.23		i14i	i13o	i11o	i14i		66
IV	PpHAK13	798	88.58		i12i	i12i	i12i	i13o		75
V	AtKUP7	858	95.36		i14i	i12i	i12i	i12i		67
VI	PpHAK6	768	85.2		i14i	i12i	i11o	i14i		67
VII	CoeloHAK7	995	108.74		i14i	i10i	o8o	o11i		71
VIII	KflHAK3	895	97.56		i13o	i12i	i11o	i13o		64
IX	CoeloHAK5	724	79.03		i12i	i12i	o10o	i11o		68
XI	KflHAK4	897	99.66		i12i	i12i	i12i	i12i		73
X	ColorbHAK1	811	89.25		NC	i10i	i11o	i12i		66
XII a	CrHAK1	1264	131.01		i13o	i12i	i11o	i15o		143
XII b	CeuHAK2	1187	127.25		i13o	i12i	i11o	i11o		154

TOPCONS generates a consensus topology derived from that obtained through the use of OCTOPUS, Philius, PolyPhobius, Scampi and SPOCTOPUS.

TOPCONS-single generates a consensus topology derived from that obtained through the use of Scampi-seq, Stmhmm, Hmmtop and Memsat.

NC: no consensus prediction (The PolyPhobius algorithm does not predict TM regions).

It was observed that this algorithm does not predict TM regions for several algae KT-HAK-KUPs.

**Table 5:** Predicted position of putative transmembrane domains in AtHAK5, HvHAK1, AtKUP7 and AtKUP4 for which considerable structural information is available [1]. Prediction models used were TOPCONS, TOPCONS-single, TMHMM 2.0 and TMpred. Columns inform on the residue number included in the predicted transmembrane domains.

**Transmembrane Domain**															
**Transporter**	**Service**	**I**	**II**	**III**	**IV**	**V**	**VI**	**VII**	**VIII**	**IX**	**X**	**XI**	**XII**	**XIII**	**XIV**

**AtHAK5**	TOPCONS	55	92	183	220	248	297	328	371	420	451	477	506	none	none
		75	115	203	240	268	317	348	391	440	471	497	526		
	TOPCONS-single	66	98	185	219	248	297	325	373	420	452	478	506	none	none
		86	118	205	239	268	317	345	393	440	472	498	526		
	TMHMM 2.0	61	96	185	220	249	297	326	369	420	451	477	506	none	none
		83	118	207	239	271	316	348	391	439	473	499	528		
	TMPred	55	97	185	220	251	296	328	368	425	452	475	507	566	none
		82	117	203	239	271	316	350	388	443	473	495	529	593	
**HvHAK1**	TOPCONS	42	82	172	211	240	287	319	361	411	442	468	497	none	none
		62	102	192	231	260	307	339	381	431	462	488	517		
	TOPCONS-single	48	85	172	210	241	289	319	364	411	443	470	496	none	none
		68	105	192	230	261	309	339	384	431	463	490	516		
	TMHMM 2.0	48	83	173	216	245	288	317	360	411	440	467	496	none	none
		70	105	195	235	267	307	339	382	433	462	489	518		
	TMPred	42	84	172	211	242	287	319	359	416	438	468	497	none	none
		60	104	192	230	263	307	340	392	434	464	485	517		
**AtKUP7**	TOPCONS	101	142	232	271	300	348	377	419	471	502	528	557	607	629
		121	162	252	291	320	368	397	439	491	522	548	577	627	649
	TOPCONS-single	105	143	233	271	299	348	379	413	471	506	536	556	none	none
		125	163	253	291	319	368	399	433	491	526	550	576		
	TMHMM 2.0	105	142	233	275	300	345	379	411	475	502	529	556	none	none
		127	164	255	293	322	367	401	433	497	524	551	578		
	TMPred	105	141	232	275	301	344	377	410	470	none	526	556	none	628
		127	164	250	296	322	367	395	434	494		546	573		644
**AtKUP4**	TOPCONS	11	51	141	179	206	255	285	329	378	410	436	464	504	537
		31	71	161	199	226	275	305	349	398	430	456	484	524	557
	TOPCONS-single	12	51	141	178	207	255	287	330	378	405	436	465	none	none
		32	71	161	198	227	275	307	350	398	425	456	485		
	TMHMM 2.0	12	54	144	181	207	256	285	327	375	407	436	465	504	541
		34	76	166	198	229	278	307	349	397	429	455	484	526	560
	TMPred	8	52	142	181	206	253	280	326	378	405	436	465	498	540
		34	68	160	198	229	275	302	349	399	432	455	482	518	561

## 2 Experimental design, materials, and methods description

Sequences corresponding to the chlorophytes *C. reinhardtii* and *C. subellipsoidea*, identified by performing Blast, were retrieved from Phytozome 12. It was also the source for sequences corresponding to the bryophyte *S. fallax* and to the marchantophyte *M. polymorpha*. Sequences from the gymnosperm *P. taeda* were retrieved from Congenie.org. In turn, sequences from the charophyte algae *C. orbicularis* and *K. flaccidum* were retrieved by performing Blast on the transcriptome shotgun assembly database. Sequences form *C. eustigma* and *Gonium pectorale* were retrieved from NCBI. The remaining sequences corresponding to the moss *Physcomitrella patients*, the lycopodiophyte *S. moellendorffii*, the gymnosperm *P. abies*, the basal angiosperm *A. trichopoda*, the dicots *A. thaliana* and *P. persica* and the monocots *O. sativa* and *Z. mays* were obtained from Nieves-Cordones et al. [2]. Sequences for *C. reinhardtii* were denoted as proposed by He et al. [4], being an additional sequence retrieved from Phytozome 12. Following retrieval of sequences a phylogenetic tree was built up, being 6 clades recognized in embryophytes and 6 corresponding to chlorophyte and charophyte algae [1]. A subset containing only green algae sequences and those of *A. thaliana* were aligned. The multiple alignment was generated with the MAFFT program (version 7) at https://mafft.cbrc.jp/alignment/server/index.html. Pair comparisons of similarity among representatives of the 12 clades, and subgroups of clades II and XII, were performed at https://www.ebi.ac.uk/Tools/psa/emboss_needle/. In order to advance on the structure of these putative transporters four services for the prediction of transmembrane domains were used to predict the transmembrane domains of KUP (TOPCONS, TOPCONS-single, THMHH 2.0 and TMPred). These prediction services were next used to analyze the possible topology of potential representatives of the above mentioned clades of green photosynthetic organisms.
