# Supplementary material for: A survey of sequences of KT-HAK-KUP transporters in green algae and basal land plants
Source: Data Brief. 2018 Jul 10;19:2356–63. doi: 10.1016/j.dib.2018.07.011 (PMC6144887; doi:10.1016/j.dib.2018.07.011)
Supplement: Supplementary file 2 — Supplementary material. [file mmc2.docx]

**Supplementary Material**

**A survey of sequences of KT-HAK-KUP transporters in green algae and basal land plants**

Guillermo E. Santa María^a,*^, Sonia Oliferuk^a^, Jorge I. Moriconi^a^

**Figure 1.** Alignment of KT-HAK-KUP sequences from *Arabidopsis thaliana*, the chlorophyte algae *Chlamydomonas reinhardtii*, *Chlamydomonas eustigma*, *Coccomyxa subellipsoidea, Gonium pectorale* and the charophyte algae *Coleochaete orbicularis* and *Klebsormidium flaccidum*. Alignment was performed with MAFFT. Orange: regions corresponding to putative transmembrane domains in AtHAK5, Red: region placed between transmembrane domains II and III, Blue: regions with homology to the first transmembrane domain and to the conserved GGT(A/L/I/P/S)F(L/A)A(S)L(V/I/M/A)YS(T/A) motif.

CrHAK2

CrHAK1

CrHAK3

CrHAK4

CrHAK2

CrHAK1

CrHAK3

CrHAK4

CrHAK2

CrHAK1

CrhAK3

CrHAK4

CrHAK2

CrHAK1

CrhAK3

CrHAK4

CrHAK2

CrHAK1

CrHAK3

CrHAK4

CrHAK2

CrHAK1

CrHAK3

CrHAK4

CrHAK2

CrHAK1

CrHAK3

CrHAK4

CrHAK2

CrHAK1

CrHAK3

CrHAK4

CrHAK2

CrHAK1

CrHAK3

CrHAK4

CrHAK2

CrHAK1

CrHAK3

CrHAK4

CrHAK2

CrHAK1

CrHAK3

CrHAK4

CrHAK2

CrHAK1

CrHAK3

CrHAK4

**Figure 2.** Topology of *E. coli* KUP transporter as predicted by four different prediction services. The position of residues that according to the work of Sato et al. [3] should be oriented towards the periplasm is shown in blue; while that of residues oriented towards the cytoplasm is shown in yellow. All these services provide an accurate agreement with data of Sato et al. [3].


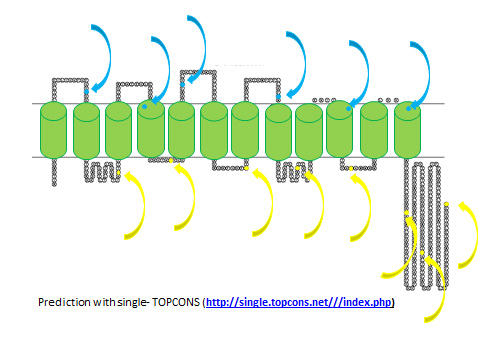

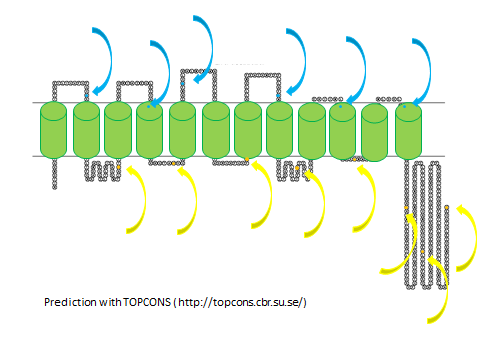


A

B

C

D


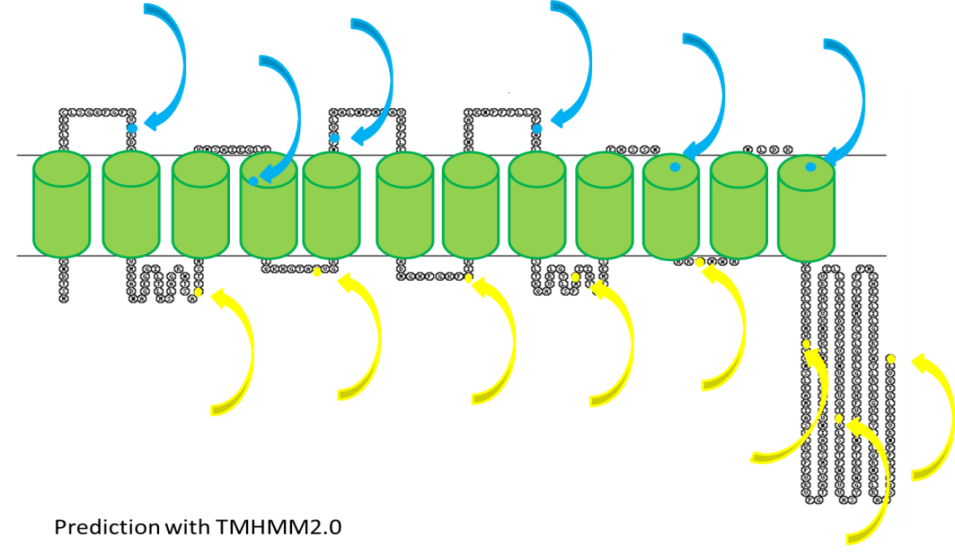

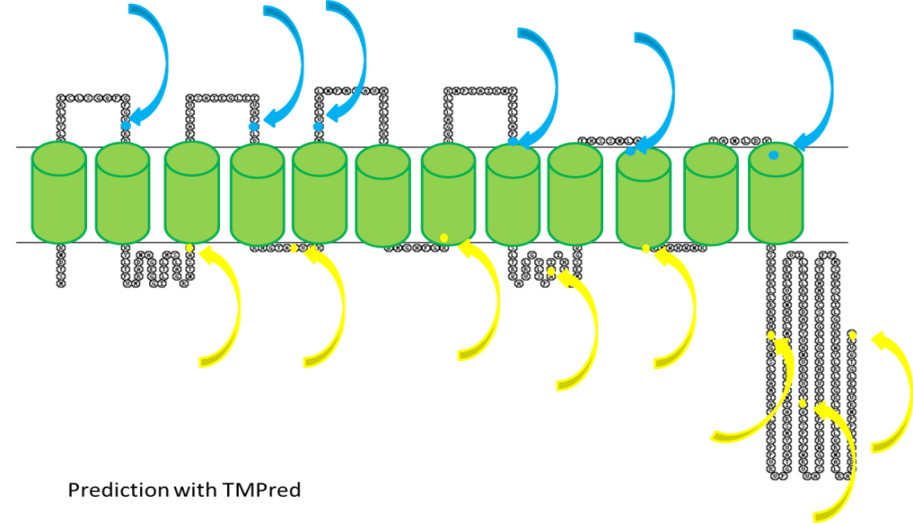


**Table: Sequences (in fasta format) used to construct Maximum Likelihood trees in Santa-María et al. [1].**

>AtHAK5

MDGEEHQIDGDEVNNHENKLNEKKKSWGKLYRPDSFIIEAGQTPTNTGRRSLMSWRTTMSLAFQSLGVVYGDIGTSPLYV

YASTFTDGINDKDDVVGVLSLIIYTITLVALLKYVFIVLQANDNGEGGTFALYSLICRYAKMGLIPNQEPEDVELSNYTL

ELPTTQLRRAHMIKEKLENSKFAKIILFLVTIMGTSMVIGDGILTPSISVLSAVSGIKSLGQNTVVGVSVAILIVLFAFQ

RFGTDKVGFSFAPIILVWFTFLIGIGLFNLFKHDITVLKALNPLYIIYYFRRTGRQGWISLGGVFLCITGTEAMFADLGH

FSVRAVQISFSCVAYPALVTIYCGQAAYLTKHTYNVSNTFYDSIPDPLYWPTFVVAVAASIIASQAMISGAFSVISQSLR

MGCFPRVKVVHTSAKYEGQVYIPEINYLLMLACIAVTLAFRTTEKIGHAYGIAVVTVMVITTLMVTLIMLVIWKTNIVWI

AIFLVVFGSIEMLYLSSVMYKFTSGGYLPLTITVVLMAMMAIWQYVHVLKYRYELREKISRENAIQMATSPDVNRVPGIG

LFYTELVNGITPLFSHYISNLSSVHSVFVLISIKTLPVNRVTSSERFFFRYVGPKDSGMFRCVVRYGYKEDIEEPDEFER

HFVYYLKEFIHHEHFMSGGGGEVDETDKEEEPNAETTVVPSSNYVPSSGRIGSAHSSSSDKIRSGRVVQVQSVEDQTELV

EKAREKGMVYLMGETEITAEKESSLFKKFIVNHAYNFLKKNCREGDKALAIPRSKLLKVGMTYEL

>AtKUP1

MNQSPSLIEQGISQQHLKTLSCANVLTLAYQSLGVIYGDLSTSPLYVYKTTFSGKLSLHEDDEEIFGVFSFIFWTFTLIA

LFKYVFIVLSADDNGEGGTFALYSLLCRYAKLSILPNHQEMDEKLSTYATGSPGETRQSAAVKSFFEKHPKSQKCLLLFV

LLGTCMAIGDSVLTPTISVLSAVSGVKLKIPNLHENYVVIIACIILVAIFSVQRYGTHRVAFIFAPISTAWLLSISSIGV

YNTIKWNPRIVSALSPVYMYKFLRSTGVEGWVSLGGVVLSITGVETMFADLGHFSSLSIKVAFSFFVYPCLILAYMGEAA

FLSKHHEDIQQSFYKAIPEPVFWPVFIVATFAAVVGSQAVISATFSIISQCCALDCFPRVKIIHTSSKIHGQIYIPEVNW

MLMCLCLAVTIGLRDTNMMGHAYGLAVTSVMLVTTCLMTLVMTIVWKQRIITVLAFVVFFGSIELLYFSSCVYKVPEGGW

IPILLSLTFMAVMYIWNYGTTKKHEFDVENKVSMDRIVSLGPSIGMVRVPGIGLVYSNLVTGVPAVFGHFVTNLPAFHKI

LVFVCVKSVQVPYVGEEERFVISRVGPKEYGMFRSVVRYGYRDVPREMYDFESRLVSAIVEFVETEPGLEEEEMSSVRRK

KEECMEIMEAKEAGVAYILGHSYAKAKQSSSVLKKLAVNVVFAFMSTNCRGTDVVLNVPHTSLLEVGMVYYV

>AtKUP2

MDLNLGKCCGSRSSKKESWRSVLLLAYQSLGVVYGDLSISPLYVFKSTFAEDIQHSETNEEIYGVMSFVFWTLTLVPLLK

YVFIVLRADDNGEGGTFALYSLICRHVKVSLLPNRQVSDEALSTYKLEHPPEKNHDSCVKRYLEKHKWLHTALLLLVLLG

TCMVIGDGLLTPAISVFSAVSGLELNMSKEHHQYAVIPITCFILVCLFSLQHFGTHRVGFVFAPIVLTWLLCISGIGLYN

IIQWNPHIYKALSPTYMFMFLRKTRVSGWMSLGGILLCITGAEAMFADLGHFNYAAIQIAFTFLVYPALILAYMGQAAYL

SRHHHSAHAIGFYVSVPKCLHWPVLAVAILASVVGSQAIISGTFSIINQSQSLGCFPRVKVIHTSDKMHGQIYIPEINWM

LMILCIAVTIGFRDVKHLGNASGLAVMAVMLVTTCLTSLVIVLCWHKPPILALAFLLFFGSIELLYFSASLTKFREGAWL

PILLSLIFMIIMFVWHYTTIKKYEFDLQNKVSLEWLLALGPSLGISRVPGIGLVFTDLTSGIPANFSRFVTNLPAFHRVL

VFVCVKSVPVPFVPPAERYLVGRVGPVDHRSYRCIVRYGYRDVHQDVDSFETELVSKLADFIRYDWHKRTQQEDDNARSV

QSNESSSESRLAVIGTVAYEIEDNLQPESVSIGFSTVESMEDVIQMAEPAPTATIRRVRFAVEENSYEDEGSTSSAEADA

ELRSELRDLLAAQEAGTAFILGHSHVKAKQGSSVMKRLAVNFGYNFLRRNCRGPDVALKVPPVSLLEVGMVYVV

>AtKUP3

MAPAESGVSPRRNPSQLSWMNLSSNLILAYQSFGVVYGDLSTSPLYVFPSTFIGKLHKHHNEDAVFGAFSLIFWTLTLIP

LLKYLLVLLSADDNGEGGTFALYSLLCRHAKLSLLPNQQAADEELSAYKFGPSTDTVTSSPFRTFLEKHKRLRTALLLVV

LFGAAMVIGDGVLTPALSVLSSLSGLQATEKNVTDGELLVLACVILVGLFALQHCGTHRVAFMFAPIVIIWLISIFFIGL

YNIIRWNPKIIHAVSPLYIIKFFRVTGQDGWISLGGVLLSVTGTEAMFANLGHFTSVSIRVAFAVVVYPCLVVQYMGQAA

FLSKNLGSIPNSFYDSVPDPVFWPVFVIATLAAIVGSQAVITTTFSIIKQCHALGCFPRIKVVHTSKHIYGQIYIPEINW

ILMILTLAMAIGFRDTTLIGNAYGIACMVVMFITTFFMALVIVVVWQKSCFLAALFLGTLWIIEGVYLSAALMKVTEGGW

VPFVLTFIFMIAMYVWHYGTRRKYSFDLHNKVSLKWLLGLGPSLGIVRVPGIGLVYSELATGVPAIFSHFVTNLPAFHKV

VVFVCVKSVPVPHVSPEERFLIGRVCPKPYRMYRCIVRYGYKDIQREDGDFENQLVQSIAEFIQMEASDLQSSASESQSN

DGRMAVLSSQKSLSNSILTVSEVEEIDYADPTIQSSKSMTLQSLRSVYEDEYPQGQVRRRHVRFQLTASSGGMGSSVREE

LMDLIRAKEAGVAYIMGHSYVKSRKSSSWLKKMAIDIGYSFLRKNCRGPAVALNIPHISLIEVGMIYYV

>AtKUP4

MADRRNRCNQILLLAYQSFGLVFGDLSISPLYVYKCTFYGGLRHHQTEDTIFGAFSLIFWTITLLSLIKYMVFVLSADDN

GEGGIFALYALLCRHARFSLLPNQQAADEEISTYYGPGDASRNLPSSAFKSLIERNKRSKTALLVLVLVGTSMVITIGVL

TPAISVSSSIDGLVAKTSLKHSTVVMIACALLVGLFVLQHRGTNKVAFLFAPIMILWLLIIATAGVYNIVTWNPSVYKAL

SPYYIYVFFRDTGIDGWLSLGGILLCITGTEAIFAELGQFTATSIRFAFCCVVYPCLVLQYMGQAAFLSKNFSALPSSFY

SSIPDPFFWPVLMMAMLAAMVASQAVIFATFSIVKQCYALGCFPRVKIVHKPRWVLGQIYIPEINWVVMILTLAVTICFR

DTRHIAFAFGLACMTLAFVTTWLMPLIINFVWNRNIVFSVLFILFFGTIELIFVASALVKIPKGGWITLLLSLFFTFITY

VWHYGSRKKYLCDQHNKVPMKSILSLGPSLGIIKVPGMGLIYTELASGVPATFKHFLTNLPAFYQVVVFVCCKTVPIPYV

PQKERYLIGRIGPKTYRMYRCIIRAGYKDVNKDGDDFEDELVMSIAEFIQLESEGYGGSNTDRSIDGRLAVVKASNKFGT

RLSRSISEANIAGSSRSQTTVTNSKSPALLKLRAEYEQELPRLSMRRMFQFRPMDTKFRQPQVKEELFDLVNAKDAEVAY

IVGHGHVKAKRNSVFVKQLVVNVAYSFLRKNCRSPGVMLNIPHICLIKVGMNYYL

>AtKUP5

MFHVEEESSGGDGSEIDEEFGGDDSTTSLSRWVFDEKDDYEVNEDYDDDGYDEHNHPEMDSDEEDDNVEQRLIRTSPAVD

SFDVDALEIPGTQKNEIEDTGIGKKLILALQTLGVVFGDIGTSPLYTFTVMFRRSPINDKEDIIGALSLVIYTLILIPLV

KYVHFVLWANDDGEGGTFALYSLICRHANVSLIPNQLPSDARISGFGLKVPSPELERSLIIKERLEASMALKKLLLILVL

AGTAMVIADAVVTPAMSVMSAIGGLKVGVGVIEQDQVVVISVSFLVILFSVQKYGTSKLGLVLGPALLLWFFCLAGIGIY

NLVKYDSSVFKAFNPAYIYFFFKRNSVNAWYALGGCVLCATGSEAMFADLSYFSVHSIQLTFILLVLPCLLLGYLGQAAY

LSENFSAAGDAFFSSVPSSLFWPVFLISNVAALIASRAMTTATFTCIKQSIALGCFPRLKIIHTSKKFIGQIYIPVLNWS

LLVVCLIVVCSTSNIFAIGNAYGIAELGIMMTTTILVTLIMLLIWQTNIIVVSMFAIVSLIVELVFFSSVCSSVADGSWI

ILVFATIMFLIMFVWNYGSKLKYETEVQKKLPMDLLRELGSNLGTIRAPGIGLLYNELAKGVPAIFGHFLTTLPAIHSMV

IFVCIKYVPVPSVPQTERFLFRRVCPRSYHLFRCVARYGYKDVRKESHQAFEQILIESLEKFIRKEAQERALESDGDHND

TDSEDDTTLSRVLIAPNGSVYSLGVPLLAEHMNSSNKRPMERRKASIDFGAGPSSALDVEQSLEKELSFIHKAKESGVVY

LLGHGDIRATKDSWFLKKLVINYLYAFLRKNSRRGITNLSVPHTHLMQVGMTYMV

>AtKUP6

MEIESGSYQNAKKESWRTVLTLAYQSLGVVYGDLSISPLYVYKSTFAEDIHHSESNEEIFGVLSFIFWTITLVPLLKYVF

IVLRADDNGEGGTFALYSLLCRHARVNSLPSCQLADEQLIEYKTDSIGSSSMPQSGFAASLKSTLEKHGVLQKILLVLAL

IGTCMVIGDGVLTPAISVFSAVSGVELSMSKEHHKYIELPAACVILIGLFALQHYGTHRVGFLFAPVILLWLMCISAIGV

YNIFHWNPHVYQALSPYYMYKFLKKTQSRGWMSLGGILLCITGSEAMFADLGHFSQLSIKIAFTSLVYPSLILAYMGQAA

YLSQHHIIESEYNIGFYVSVPERLRWPVLVIAILAAVVGSQAIITGTFSIIKQCSALGCFPKVKIVHTSSKIHGQIYIPE

INWILMVLCLAVTIGFRDTKRLGNASGLAVITVMLVTTCLMSLVIVLCWHKSVIFAIVFVVFFGTIESLYFSASLIKFLE

GAWVPIALAFCFLLAMCTWHYGTLKRYEYDVQNKVSVNWLLSLSQTLGIARVRGLGLIHTELVSGVPAIFSHFVTNLPAF

HQVLVFLCVKSVPVPHVRPQERFLVGRIGPKEFRIYRCIVRFGYRDVHKDDFEFEGDLVCSIAEFIRTEAETAATAAETN

GEDDDRMSVVGTCSTYMQGIEDHYESDIDDPDKPGTSEIRSPKPKKKSKSKVKKRVRFVVPETPKIEKETRQELMELTEA

REGGVAYIMGNAYMKAKPGSGLLKRLAINIGYEFLRRNTRGPRNMLTSPHASTLEVGMIYNV

>AtKUP7

MAEESSMEGSEKEEIDSSGGGFGDMASMDSIESRWVIQDDDDSEIGVDDDNDGFDGTGLESDEDEIPEHRLIRTGPRVDS

FDVEALEVPGAPRNDYEDLTVGRKVLLAFQTLGVVFGDVGTSPLYTFSVMFSKSPVQEKEDVIGALSLVLYTLLLVPLIK

YVLVVLWANDDGEGGTFALYSLISRHAKISLIPNQLRSDTRISSFRLKVPCPELERSLKLKEKLENSLILKKILLVLVLA

GTSMVIADGVVTPAMSVMSAVGGLKVGVDVVEQDQVVMISVAFLVILFSLQKYGTSKMGLVVGPALLIWFCSLAGIGIYN

LIKYDSSVYRAFNPVHIYYFFKRNSINAWYALGGCILCATGSEALFADLCYFSVRSVQLTFVCLVLPCLMLGYMGQAAYL

MENHADASQAFFSSVPGSAFWPVLFIANIAALIASRTMTTATFSCIKQSTALGCFPRLKIIHTSRKFMGQIYIPVLNWFL

LAVCLVVVCSISSIDEIGNAYGMAELGVMMTTTILVTLIMLLIWQINIVIVIAFLVVFLGVELVFFSSVIASVGDGSWII

LVFAVIMFGIMYIWNYGSKLRYETEVEQKLSMDLMRELGCNLGTIRAPGIGLLYNELVKGVPAIFGHFLTTLPAIHSMVI

FVCIKYVPVPVVPQNERFLFRRVCTKSYHLFRCIARYGYKDARKETHQAFEQLLIESLEKFIRREAQERSLESDGNDDSD

SEEDFPGSRVVIGPNGSMYSMGVPLLSEYRDLNKPIMEMNTSSDHTNHHPFDTSSDSSVSEAEQSLERELSFIHKAKESG

VVYLLGHGDIRARKDSWFIKKLVINYFYTFLRKNCRRGIANLSVPQSHLMQVGMTYMV

>AtKUP8

MDLERLSPRNPVKKESWWTVLTLAYQSLGVVYGDLATSPLYVYKSTFAEDITHSETNEEIFGVLSLIFWTLTLIPLVKYV

FIVLRADDNGEGGTFALYSLLCRHARISSLPNFQLADEDLSEYKKNSGENPMRLKVPGWSLKNTLEKHKFLQNMLLVLAL

IGTCMVIGDGVLTPAISVFSAVSGLELSMSKQQHQYVEVPVVCAILILLFSLQHYGTHRLGFVFAPIVLAWLLCISTIGV

YNIFHWNPHVYKALSPYYIYKFLKKTRKRGWMSLGGILLCITGSEAMFADLGHFTQLSIQIAFTFAVYPSLILAYMGQAA

YLSKHHVLQSDYRIGFYVSVPEQIRWPVLAIAILAAVVGSQAIITGTFSIIKQCTSLGCFPKVKIVHTSSRMHGQIYIPE

INWTLMLLCLAVTVGFRDTKHISNASGLAVITVMLVTTCLMSLVIVLCWRKSSLYALAFIFFFGTIEVLYFSASLIKFLE

GAWVPVALSFIFLLIMYVWHYGTLKRYEFDVQNKVSINWLLTLFGSSNLGIVRVHGIGVINTELVSGIPAIFSHFITNLP

AFHQVVVFLCVKSVPVPHVKPEERFLVGRVGPKEYRLYRCIARYGYRDVHKDDVEFENDLICSIAEFIRSDKPLNYSPDP

ENESGINERLTVVAASSSNLEGVQIYEDDGSDKQEPSSSSEVIMVAPSPRFKKRVRFVLPESARIDRSAEEELTELTEAR

EAGMAFIMGHSYVRAKSGSSVMKKIAINFGYDFLRRNSRGPCYGLSTPHASTLEVGMVYIV

>AtKUP9

MAERVEASSVPEGENTIEEREVGAMWELEQKLDQPMDEEANKLNNMYREKGLSMLMLLRLSFQSLGIVYGDLGTSPLYVF

YNTFPDGIDDSEDVIGALSLIIYSLLLIPLIKYVFIVCKANDNGQGGTLAIYSLLCRHAKVKLIPNQHRSDEDLTTYSRT

VSAEGSFAAKTKKWLEGKEWRKRALLVVVLLGTCMMIGDGILTPAISVLSATGGIKVNNPKMSGDIVVLVAIVILIGLFS

MQHYGTDKVGWLFAPIVLIWFLFIGATGMYNICKYDTSVLKAFSPTYIYLYFKRRGRDGWISLGGILLSITGTEALYADI

AYFPLLAIQLAFTFFVFPCLLLAYCGQAAYLVIHKEHYQDAFYASIPDSVYWPMFIVATGAAIVGSQATISGTYSIVKQA

VAHGCFPRVKIVHTSKKFLGQIYCPDINWILMLGCIAVTASFKKQSQIGNAYGKMTTTSKYKKNYFSQWTAVVLVMLVTT

LLMVLIMLLVWHCHWILVLIFTFLSFFVELSYFSAVIFKIDEGGWVPLIIAAISLLVMSVWHYATVKKYEFEMHSKVSMS

WILGLGPSLGLVRVPGIGLVYTELASGVPHIFSHFITNLPAIHSVVVFVCVKYLPVYTVPEEERFLVKRIGPKTFRMFRC

VARYGYKDLHKKDDDFENKLLTKLSSFIRIETMMEPTSNSSTYSSTYSVNHTQDSTVDLIHNNNNHNHNNNMDMFSSMVD

YTVSTLDTIVSAESLHNTVSFSQDNTVEEEETDELEFLKTCKESGVVHIMGNTVVKARTGSWLPKKIAIDYVYAFLAKIC

RANSVILHVPHETLLNVGQVFYV

>AtKUP10

MAGRVESSIGGGEIDEEGDERGSMWDLDQSLDQPMDEEAGRLRNMYREKKFSAFLLLQLSFQSLGVVYGDLGTSPLYVFY

NTFPRGIKDPEDIIGALSLIIYSLTLIPLLKYVFVVCKANDNGQGGTFALYSLLCRHAKVSTIPNQHRTDEELTTYSRTT

FHERSFAAKTKRWLENGTSRKNALLILVLVGTCMVIGDGILTPAISVLSAAGGLRVNLPHINNGIVVVVAVVILVSLFSV

QHYGTDRVGWLFAPIVFLWFLFIASIGMFNIWKHDPSVLKAFSPVYIFRYFKRGGQDRWTSLGGIMLSITGIEALFADLS

HFPVSAVQFAFTVIVFPCLLLAYSGQAAYLRKYPHHVEDAFYQSIPKRVYWPMFIIATAAAIVASQATISATFSLIKQAL

AHGCFPRVKVVHTSRKFLGQIYVPDINWILMILCIAVTAGFKNQNQIGNAYGTAVVIVMLVTTLLMMLIMILVWRCHWVL

VLLFTLLSLVVECTYFSAVLFKVNQGGWVPLVIAAAFLVIMYVWHYGTLKRYEFEMHSKVSMAWILGLGPSLGLVRVPGI

GLVYTELASGVPHIFSHFITNLPATHSVVIFVCVKNLPVYTVPQEERFLVKRIGPKNFHMFRCVARYGYRDLHKKDDDFE

KRLFESLFLFLRLESMMEGCSDSEDYSVCGSQQRQSRDGVNGNGNEIRNVSTFDTFDSIESVIAPTTTKRTSHTVTGSSQ

MSGGGDEVEFINGCRDAGVVHIMGNTVVRARREARFYKRIAIDYVYAFLRKICRENSAIFNVPQESLLNVGQIFYV

>AtKUP11

MAARVEAATMGGEIDEEESDERGSMWDLDQKLDQSMDEEAGRLRNMYREKKFSALLLLQLSFQSLGVVYGDLGTSPLYVF

YNTFPHGIKDPEDIIGALSLIIYSLTLIPLLKYVFVVCKANDNGQGGTFALYSLLCRHAKVKTIQNQHRTDEELTTYSRT

TFHEHSFAAKTKRWLEKRTSRKTALLILVLVGTCMVIGDGILTPAISVLSAAGGLRVNLPHISNGVVVFVAVVILVSLFS

VQHYGTDRVGWLFAPIVFLWFLSIASIGMYNIWKHDTSVLKAFSPVYIYRYFKRGGRDRWTSLGGIMLSITGIEALFADL

SHFPVSAVQIAFTVIVFPCLLLAYSGQAAYIRRYPDHVADAFYRSIPGSVYWPMFIIATAAAIVASQATISATFSLVKQA

LAHGCFPRVKVVHTSRKFLGQIYVPDINWILMILCIAVTAGFKNQSQIGNAYGTAVVIVMLVTTLLMTLIMILVWRCHWV

LVLIFTVLSLVVECTYFSAMLFKIDQGGWVPLVIAAAFLLIMWVWHYGTLKRYEFEMHCRVSMAWILGLGPSLGLVRVPG

VGLVYTELASGVPHIFSHFITNLPAIHSVVVFVCVKNLPVYTVPEEERFLVKRIGPKNFHMFRCVARYGYRDLHKKDDDF

EKRLFESLFLYVRLESMMEGGCSDSDDYSICGSQQQLKDTLGNGNENENLATFDTFDSIESITPVKRVSNTVTASSQMSG

VDELEFINGCRDAGVVHIMGNTVVRARREARFYKKIAIDYVYAFLRKICREHSVIYNVPQESLLNVGQIFYV

>AtKUP12

MEEIEEGSSNNSIRRVGTGSSDRRWVDGSEVDSETPLFSEIRDRDYSFGNLRRRLMKKPKRADSLDVEAMEIAGSHGHNL

KDLSLLTTLGIAFQTLGVVYGDMGTSPLYVFSDVFSKVPIRSEVDVLGALSLVIYTIAVIPLAKYVFVVLKANDNGEGGT

FALYSLICRYAKVNKLPNQQPADEQISSFRLKLPTPELERALGIKEALETKGYLKTLLLLLVLMGTSMIIGDGILTPAMS

VMSAMSGLQGEVKGFGTNALVMSSIVILVALFSIQRFGTGKVGFLFAPVLALWFFSLGAIGIYNLLKYDFTVIRALNPFY

IVLFFNKNSKQAWSALGGCVLCITGAEAMFADLGHFSVRSIQMAFTCVVFPCLLLAYMGQAAYLTKHPEASARIFYDSVP

KSLFWPVFVIATLAAMIASQAMISATFSCVKQAMALGCFPRLKIIHTSKKRIGQIYIPVINWFLMIMCILVVSIFRSTTH

IANAYGIAEVGVMMVSTVLVTLVMLLIWQTNIFLALCFPLIFGSVETIYLLAVLTKILEGGWVPLVFATFFLTVMYIWNY

GSVLKYQSEVRERISMDFMRELGSTLGTIRIPGIGLLYNELVQGIPSIFGQFLLTLPAIHSTIIFVCIKYVPVPVVPQEE

RFLFRRVCPKDYHMFRCIARYGYKDVRKEDSRVFEQLLIESLEKFLRCEALEDALESTLNDFDPDRVSVASDTYTDDLMA

PLIHRAKRSEPEQELDSEVLPSSSVGSSMEEDPALEYELAALREATDSGLTYLLAHGDVRAKKNSIFVKKLVINYFYAFL

RRNCRAGAANLTVPHMNILQAGMTYMV

>OsHAK1

MSSALEVEGSGSPGVEPAATATASRLKRHDSLFGDAEKVSGGKHHGGSAVSWAVTLHLAFQSVGIIYGDIGTSPLYVYSS

TFPDGIGHRDDLVGVLSLILYTLIIIPMLKYVFIVLYANDNGDGGTFALYSLISRYAKIRMIPNQQAEDAMVSNYSIEAP

SSQLRRAQWVKHKLESSRAAKMALFFLTILGTSMVMGDGTLTPAISVLSAVSGIREKAPNLTQTQVVLISVAILFMLFSV

QRFGTDKVGYTFAPIISVWFLLIAGIGLYNLVVHEITILKAFNPWYIVQYFRRNGKKGWVSLGGVVLCVTGTEGMFADLG

HFNIRAVQISFNCILFPSVALCYIGQAAYLRKFPENVSDTFYKSIPGPLFWPTFIVAILAAIIASQAMLSGAFAILSKAL

SLGCLPRVRVIHTSKKYEGQVYIPEVNFMMGLASIIVTIAFRTTTSIGNAYGICVVTTFMVTTHLMTVVMLLIWKKHLVF

ILLFYCVFGFTEVVYLSSILSKFVDGGYLPFCFAMVLMTMMATWHYVHVRRYWYELDHIVPTAELASLLEENGGVRRVPG

VGLLYTELVQGIPPLFPRLVRKIPSVHAVFVFISIKHLPIPHVAAAERFLFRQVGPRARRVFRCVARYGYTDALEEPREF

AAFLVDGLKMFIQEESAFAPHQEMIDAAADDDDEAAARPRRSTSSAVHSEEAIQAASSGRTTASSVQLQAGGEPPAAMDV

EEEKRLIDREVGRGVVYLMGEANVSAGPNSSILKRIAVNYIYTFLRKNLTEGHRALAIPNDQLLKVGITYEI

>OsHAK2

MDAEAGVGGADQLPWRQHYRNLLLLAYQSFGVVYGDLSTSPLYVYKSTFSGRLRRYQDEQTVFGVLSLIFWTFTLIPLLK

YVTIVLSADDNGEGGPFALYSLLCRHAKLSFLPNQQSADEELSTYYRNGFTSRHGSLPWLRRFMEKHKNARTVLLLIVLC

GASMMIGDGILTPAISVLSSMSGLKVRATGLHDRSVVLLSCIVLVGLFALQHRGTQKVAFMFAPIVVIWLFCIGGIGLYN

IIHWNPRIYQALSPYYIVKFFRTTGKDGWIALGGILLSMTGCEAMFADLGHFTSASVRLAFITIIYPCLILQYMGQAAFL

SKNILDMPTGFYDSIPGPIFWPVFVVATLAAVVGSQAVISATFSIVKQCHSLGCFPRVKVVHTSRWIYGQIYIPEINWIL

MVLCVAVTVAFRDITLIGNAYGVACMTVMFVTTFLMALIMIFVWQKNIIFALSFFLLFGSVEVVYLSSSLMKVTQGGWVP

LVLALIFMSVMYIWHYGTRKKYQYDLQNKVSMRYILSLGPSLDVVRVPGIGLIYTELVTGVPNIFTHFTTNLPAFHEVLV

FLCVKSVPVPYVSPDERYLVGRIGPRAYRMYRCIVRYGYKDVQRDDDNFENMLVMNIGKFIMMEAEDASSSASYDTANEG

RMAVITTSDDYDSPLAVRDSNDLADSMTMRSTKSESLRSLQSSYEQESPNVSRRRRVRFELPEEDDMDQQVKDELLALVE

AKHTGVTYVMGHVYIKARKNSSFFKRFAIDVGYSFLRKNCRGPSVTLHIPHISLIEVGMAYQV

>OsHAK3

MPVADCESGLSPADVTGAGAANGNPGHWRSYYRHVLLLAYQSCGVVYGDLSTSPLYVYKSTFIIGSLRRFQDEEIVFGVF

SLVFWTLTLIPLLKYVFIVLAADDNGEGGTFALYSLLVRHAKFSLMPNQEAADEELTSYYRPGYAPQETPILTALRRFLE

NHRKSRTFLLVTVLFGASLVIGDGVLTPPMSVLSSFSGLQVHSTALTSGEVEILSCTVLVCLFMVQHWGTHRVAFLFAPV

VIVWLLLLGALGVYNIVVWNPRVLRALSPYYLVRFFQHTGKDGWISLGGILLSMTGTEAMYADLGHFTAASIRVAFVGLI

YPCLVLQYMGQAAFLSKSPHCDIHFVFFESIPTGIFWPVLVIATLAAIVGSQAVISATFSIVRQCTALGCFPRVKIVHTS

RRIHGQIYSPEINWILMLLCIAVTMGLRDTTLIGNAYGMACAGVMLVTTLLMALVIVFVWQYSCLVAALFLVAFGVVEAV

YLSAALMKVPQGGWLPLVLSLVFVAVMYVWHYGTRRKHQFDVQNKVSLRWIHALGPSLGIVRVPGIGIIYSELATGVPAI

FSHFVTNLPAFHQVLVFICVKAVPVPHVRDEERHLVGRIGPREFRMYRCVVRHGYKDVLAEDTDFENDLVLRIAEFVQME

ADFDQRCSISDDGVVASVEVEGRMAVVPRPSDLARTGLLMREPGEEESVVARAAAAAKPESLIHSMHTMHEAESPGFASR

RRVRFEVANQHTDPRVKEELSALVEAKHAGVAYIMGHSYIKARKSSSVFKKFAVNVAYAFLRKNCRGPGLVLNIPHISLI

EVGMIYYV

>OsHAK4

MSSSHTVTVSMDVEAGQKNKDKKGISQDLILAYKTLGVVFGGLVTSPLYVYPSMNLTNPTEEDYLGIYSIMFWTLTLIGV

VKYICIALNADDHGEGGTFAMYSLLCQHANIGILPSKKIYTEEENLISNQPVVAGRPGRLRRFIESSIIARRLLLLTAIL

GMCMLIGDGILTPAISVLSAIDGLRGPFPSVSKPAVEGLSAAILVGLFLLQKYGTSKVSFMFSPIMAAWTFATPVIGVYS

IWRYYPGIFKAMSPHYIVRFFMTNQTRGWQLLGGTVLCITGAEAMFADLGHFSKRSIQIAFMSSIYPSLVLTYAGQTAYL

INNVDDFSDGFYKFVPRPVYWPMFIIATLAAIVASQSLISATFSVIKQSVVLDYFPRVKVVHTSKDKEGEVYSPETNYML

MLLCVGVILGFGDGKDIGNAFGVVVILVMLITTILLTLVMLIIWGTHVVLVALYLVPFLLLEATYVSAVCTKILRGGWVP

FAVSVALAAVMFGWYYGRQRKTEYEAANKVTLERLGELLSGPGLRRVPGLCFFYSNRQDGGWLTPVLAHYIRNMRSLHEV

TVFLTLRYLLVAKVDGKDRVQAVRRLGPAGVYGCTIQYGYADAIDFEEDDIAGQVVGALRERVVDGEEEGERVEAARAAG

VVHVRGKMRFHVGKDTRLFDRVLLGFYELLHGACRSALPALGIPLQQRVEIGMLYKA

>OsHAK5

MTEPLHTSSNGGAERGPNAAFESEKTLQTTTRLQRFDSLHMEAGKIPGGQSHTAKVGWATTLHLAFQSIGVVYGDMGTSP

LYVFSSTFTNGIKDTNDILGVMSLIIYTVVLLPLIKYCFIVLRANDNGDGGTFALYSLISRYARISLIPNQQAEDAMVSH

YKLESPSNRVKRAHWIKEKMENSPNFKIILFLVTILATSMVIGDGVLTPCISVLSAVGGIKESAKSLTQGQIAGIAIAIL

IVLFLVQRFGTDKVGYSFGPIILTWFIFIAGTGVYNLFKHDTGVLKAFNPKYIVDYFERNGKQGWISLGGVILCITGTEA

MFADLGHFNVRAIQIGFSVVLLPSVLLAYIGQAAYLRIYPEHVADTFYKSIPDPLYWPTFVVAVAAAIIASQAMISGAFA

IIAQSQILGCFPRVRVIHTSTKFHGQVYIPEINYVLMVLCVAVTAIFQTTDKIGNAYGIAVVFVMFITTLLVTLVMVMIW

KTSLLWIALFPVIFGGAELIYLSSAFYKFTQGGYLPLVFSAILMFIMATWHYVHVHRYKYELRNKVSNNYVAELAVKQNL

ARLPGIGFLYSELVQGIPPILPHLVEKVPSIHSVLVIISIKYLPISKIETKERFLFRYVEPKEYRVFRCVVRYGYNDKVE

DPAEFESLVIENLKQFIHEESLYSQSSHSLEGESIKEIGGVTDPTSEVQDAMSSRNNSDQHTTEPRNGCMDEIQSIHKEM

GNGVVHLLGETNVVAEPNADFLKKIIVDYVYNFIRKNFRQPEKITCVPHNRLLRVGMTYEI

>OsHAK6

MVPPGNGNGAAAAAGNDVILELSTPGDDWSHELQGDDVEANGGGNGDAPPRRTFSFGQAYKTRHRQPQVFTVWQTLMLGY

QSLGIVYGDLGTSPLYVFPSVVLPDADATDFLGILSLIIWTLTLMSLVKYALIVLKADDHGEGGTFALYSLLRQHVNFKG

NIPVPLTRLESDVHLKFHSKRRSRPSRLQLFLENSPKAQLAITIIVLIGTCMLIGDGALTPAISVLSAVQGIQSRSSHIK

QKHVVVLSAVILVLLFLVQRFGTSRVSFTFSPIMLLWFASIAGIGVYNIVMHYPPVLKAVSPHYIYYYFAKNKRVGWEQL

GAVILCITGAEAMFADMGHFNKSSIQVAFSTAVFPSLILAYSGQAAYLIKNPGDLSTAFYSSVPAPLFWPMFVVSTLAAI

VASQSLISASYSIIRQSIALGCFPRTTVKHTSDKYEGQVYCPEINYVLMVVCVLITVGFQGGPEIGRAFGVAVIWVMLLT

TTLMTVVMVVIWEVNGALAGGFFVFYLAIEGTYMTSLMTKVPQGGWVPFAITVAFLSVTLSWTYGRKKKREYEARHAVGD

GEFAGIVSRSARVPGMCLFCTDLMDGVPPIVRHYAANTGSLRELLLFVTFRTLPVRTVLAGERFLVAREGARAGVYRCIA

QYGYMDEQDMVGDDFVRAAVAALVEVAAAAAEADSGEEEAEMIGRAPASGVSYVIGRTVLRMRRARNWPKRFVINELYRF

LQKNFRSNVSTLKLDHAKTLQVGMIYEI

>OsHAK7

MPSYQYLLSLLFYILDCTDRFSVIVTIHNHRVGVLMIVLLQDQWKSYCRTISLLAFQSFGVVYGDLSTSPLYVYKSAFSG

RLNNYRDETTIFGLFSLIFWTLTLLPLLKYVIIVLNADDNGEGGTFALYSLLCRHAKFSLLPNQQSADEELSTYYQPGVG

GIISSPLKRFLEKHRKLRTCLLLFVLFGACMVIGDGVFTPAISVLSAISGLKDPGPGGIPDGWVVFIACIVLVGLFALQH

RGTHRVAFMFAPIVVVWLLSIGVIGLYNIIHWNHRIFLALSPHYVIKFFKMTGKDGWLSLGGVLLAITGTEAMFADLGHF

TAASIRLAFVGAIYPCLVLQYMGQAAFLSRNMSAVEDSFYQSVPRSLFWPVFVIATLAAVVGSQSIISATFSIVKQCLSL

GCFPRVKVVHTSRWIHGQIYIPEINWILMVLCLAVTLGFRDTTVIGNAYGLACIVVMFVTTWLMALVIIFVWQKNILLAL

LFVVAFGSIEVVYLSAAVTKVPQGGWAPIVFAFVFMLVMYVWHYGSRRKYLFDLQNKVSMKWILTLGPSLGIVRVPGIGL

IYTELVTGVPSIFSHFVTNLPAFHQVLVFVCVKSVPVPFVPEDERYLIGRIGPREYRMYRCIVRYGYKDVQKDDENFENH

LVMSIAKFIQMEAEEAASSGSYESSEGRMAVIHTEDTTGTGLVMRDSNNEASGTSLTRSSRSETLRSLQSIYEQESGSLS

RRRRVRFEIAEEERIDPQVRDELADLLDAKEAGVTYIIGHSYVKARKNSNFLKTFAIDYAYSFLRKNCRGPAVALHIPHI

SLVEVGMIYYV

>OsHAK8

MDLEFGRGMRSPQRDSWKTTLLLAYQSLGVVYGDLSISPLYVFKSTFAEDIQHSETNEEIFGVLSFVFWTLTLIPLIKYV

SIVLRADDNGEGGTFALYSLICRHANVSLLPNRQIADEELSTYKLECSSERTDKSCIKVWLEKHKKLHTALLIMVLIGTC

MVIGDGVLTPAISVFSAVSGLEFSLSKDHREYAVIPITCVILAFLFALQHYGTHRVGFLFAPIVLAWLICMSALGLYNII

HWNPHVYQALNPCYMFKFLKKTRKYGWMSLGGILLCMTGSEAMFADLGHFSYSAIQLAFTSLVYPALILAYMGQAAYLSK

HHDFYSNSQVGFYIAVPDKVRWPVLVLAILASVVGSQAIISGTFSIINQSQSLSCFPRVKVVHTSDKIHGQIYIPEINWL

LMILCIAVTVGFRDTKHMGNASGLAVITVMLVTTCLTSLVIMLCWRRPPVLALCFLLFFGSVEALYFSASLIKFLEGAWL

PILLALFLMAVMLVWHYTTIKKYEFDLHNKVTLEWLLALGDKLGMVRVPGIGLVYTDLTSGVPANFSRFVTNLPAFHQVL

VFVCVKSVPVPYVFPAERYLIGRVGPPGHRSYRCIVRYGYRDVHQDVDSFETELVESLATFIKLDASYRCSDASGGGGDH

EPEEERGTRLAVIGSSHASYDIQDSVQHSSAASVETTTTRRRSGGGDDDGSPGGGGGRAKQVRFFIDSHVASPEAADNKQ

VAEELEALAAARDAGTAFILGHSHVQCKPGSSLLKRLAVDVGYNFLRRNCRGPDVALRVPPASLLEVGMVYVL

>OsHAK9

MDPEFGRGMAPRKREPWRTTLLLAYQSLGVVYGDLSISPLYVYKSTFAEDITHSESNEEIFGVLSFVFWTLTLIPLIKYV

SIVLRADDNGEGGTFALYSLICRHANVSLLPNRQVADEELSTYKLEYPPEVANRSRIKEWLEKHKTLQTALLIMVMIGTC

MVIGDGVLTPAISVFSAVSGLELSLSRDQHEYAVIPITCVILVFLFALQHYGTHRVGFLFAPIVLAWLICMSMLGLYNII

HWNPQVYRALNPYYMLKFLRKTKKSGWMSLGGILLCMTGSEAMFADLGHFSYSAIQLAFTTLVYPALILGYMGQAAYLSK

HHTLNSTYQIGYYISVPESVRWPVLVLAILASVVGSQAIISGTFSIINQSQSLSCFPRVKVVHTSENIHGQIYIPEINWL

LMVLCIAVTVGFRDTKHMGNASGLAVITVMLVTTCLTSLVIMLCWHRSPALALVFFLFFGSIEVLYFSASLIKFREGAWL

PIMLALILMAVMFIWHHTTIKKYEFDLHNKVTLEWLLALGDKLGMVRVPGIGLVYTDLTSGVPANFSRFVTNLPAFHRVL

VFVCVKSVPVPHVLPAERYLVGRVGPAGHRSYRCIVRYGYRDVHQDVDSFEAELVESLATFIKLDALYHRCSDAGSGSEQ

LDDGRYERENALTVIGTNPLRRCLSYEASHDGVSSVDAARSPNGIVEVPAAAAAAPVTKKVRFVVEAASPEVEKGVVEEL

QELCEAREAGTAFILGHSHVQTKPGSSLLKKLAVGVGYNFLRRNCRGPDVVLRVPPASLLEVGMVYVL

>OsHAK10

MKSPSPVDPESPSSPDCKGGSSSKRRRLPWRMTMSLAYQSLGVVYGDLSTSPLYVYKAAFAEDIQHSETNEEILGVLSFV

FWTLTLVPLLKYVCVVLRADDNGEGGTFALYSLLCRHARAALLPPGGGGGGGEPGDEDQFLDAGADKKAAANGNALALSG

RGGGGGAAAGVRRLLERHKVLQRVLLVLALVGTCMVIGDGVLTPAISVFSAVSGLELSMEKHQHKYVEVPIACFVLVCLF

CLQHYGTHRVGFLFAPIVITWLLCISMIGVYNIVHWEPNVYRALSPYYMYKFLKKTQRGGWMSLGGILLCITGSEAMFAD

LGHFNQLSIQIAFTCMVYPSLILAYMGQAAYLCKHHIIESDYRIGFYVSVPEKIRWPVLAIAILAAVVGSQAVITGTFSM

IKQCTALGCFPRVKIVHTSDKVHGQIYIPEINWILMILCLAITIGFRDTKHLGNASGLAVITVMLVTTCLMSLVIVLCWH

KSIFLAFGFIIFFGTIEALYFSASLIKFREGAWVPIVLAFIFMAIMCIWHYGTIKKYEFDLQNKVSINWLLGLSPNLGIV

RVRGIGLIHTELDSGIPAIFSHFVTNLPAFHQVLIFLCIKNVPIPHVSPEERFLVGRIGPKEYRIYRCIVRYGYHDVHKD

DQEFEKELVCSVAEFIRSGAAAAADAAASSKPKNVCGGGAEESEKEEEERMSVIPSGSIRMMEEDGGAGAPSSEDTVGGS

GSGSGRGSSRGGGGAREIMSPSPSPPPVVVAPRKRVRFVLPAASPRPDAGVREELQELMDAREAGMAFILGHSYVKAKSG

SSFFRRLVINFCYDFLRRNSRGPNYAVTIPHASTLEVGMIYYV

>OsHAK11

MASLSESEGTNRGSMWELDQNLDQPMDEEASRLKNMYREKKFSSLLLLRLAFQSLGVVFGDLGTSPLYVFYNAFPHGVDD

EEDVIGALSLIIYTLTLIPLLKYVFVVLRANDNGQGGTFALYSLLCRHAKISTIPNQHKTDEDLTTYSRQTYEENSVGAK

IKRWLEAHAYKRNCLLIVVLIGTCTAIGDGILTPAISVLSASGGIKVQNPNMSTDVVVIVSVIILIGLFSMQHYGTDKVG

WLFAPIVLLWFILIGSVGALNIHKYKGSVLKAYNPVYIYRYFQRRNSDSWASLGGIMLSITGTEALFADLCHFPVFAIQI

AFTLIVFPCLLLAYTGQAAYIIAHKDHVADAFYRSIPDSIYWPAFVIATAAAIVASQATISATYSIIKQALALGCFPRVK

IVHTSKKFLGQIYIPDINWVLLILCIAVTAGFKNQSQIGNAYGTAVVIVMLVTTFLMVPIMLLVWKSHWILVVTFIVLSL

MVEIPYFSACLLKIDQGGWVPLVIATAFFIIMYVWHFCTVKRYEFEMHSKVSMAWILGLGPSLGLVRVPGIGFVYTELAS

GVPHIFSHFITNLPAIHSVVVFVCVKYLPVYTVPMDERFLVRRIGPKNFHIFRCVARYGYKDLHKKDEDFEKMLFNCLLS

FLRLESMMEGYSDSDDFSVPEQRTEGSISNAFLAEKTNNNTMCSHGDLSYSSQDSIVPVQSPLRGNSLLRYSSQASHTVS

DELEFLNRCKDAGVVHILGNTIVLARRDSGIIKKIAVNYMYAFMRKICRENSVIFNVPHESLLNVGQIYYI

>OsHAK12

MASISDSETTNHGSIWDLDQNLDQPMDEEASRLKNMYTEKKFSSILLLRLAFQSLGVVFGDLGTSPLYVFYNIFPHGVDD

DEDVIGALSLIIYTLTLIPLMKYVFVVLRANDNGQGGTFALYSLLCRHAKVSTIPNQHKTDEELTTYSRQTYEENSLAAK

IKRWLEGHVYKKNCLLILVLIGTCTAIGDGILTPAISVLSASGGIRVQNQKMSTDVVVVVAVIILIGLFSMQHYGTDKVG

WLFAPIVLLWFILIGTIGALNIHKYNSSVLKAYNPVYIYRYFRRGKSESWTSLGGIMLSITGTEALYADLCHFPVLAIQI

AFTLVVFPCLLLAYTGQAAYIISNKDHVVDAFYRSIPDTIYWPVFIIATLAAIVASQATISATYSIIKQALALGCFPRVS

VVHTSKKFLGQIYIPDINWVLMILCIAVTAGFKNQSQIGNAYGTAVVIVMLVTTFLMVPIMLLVWKSHWILVVIFIVLSL

MVELPYFTACINKVDQGGWVPLVVATTCFIIMYVWHFCTVKRYEFEMHSKVSMAWILGLGPSLGLVRVPGIGFVYTELAS

GVPHIFSHFITNLPAIHSVVVFVCVKYLPVYTVPTEERFIVKRIGPKNFHMFRCVARYGYKDIHKRDDDFEKMLLDRLLL

FVRLESMMDDYSDSEDFTMMEEKTQGSSNALLLTGKAGSNTMCSTGDLSYSSQDSIVPAKSPIRGNSLTRYSSQTFGDEL

EFLNLEFLNRCKDAGVVHILGNTVVHARPDSGIIKKVAVNYVFAFLRKICRENSVIFNVPHESLLNVGQIYYI

>OsHAK13

MDVEGGGGGGGGAPPRGRNSWGWQKGTLLLAYQSFGVVYGDLCISPVYVYKNTFSGKLRLHEEDEEILGVLSLVFWSLTL

IPLLKYIILVLGADDNGEGGTFALYSLLCRNSKMGLLNNMRANHGSLSAYNKEEPCKESRNSMLIKAFFEKHYSLRVVLL

LFVLMGTSMVIGDGVLTPTMSVLAAVSGLRIKFPELHENYTVLLACVILIGLFALQHYGTRRVGFLFAPILISWLTCIGG

IGIYNIIKWNPSVIRALSPYYIYNFFRKAGKDGWSSLGGIVLCLTGAEAMFADLGHFSKLSLRLGFTIVVYPCLVLAYMG

EAAYLSKHREDLQSSFYKALPDRVFWPVLFIATLATAVGSQAIISATFSIISQCRALGCFPRIKVVHTSSHVHGQIYIPE

VNWVLMSLCLAVTIGFRDTEMIGNAYGLAVILVMCATTCLMFLVITTVWNRWVVWAAAFTVVFGSVELLYLSACLAKVPH

GGWLPLLLSLTTLLVMSTWHYGTAMKQQHEVQNKVCLDHFLGLSSGIGLVRVPGVGFVYSSTTNGVPPMFAHFVTNFPAF

HRVLIFVSLQTLAVPKVSPEERFLVGRIGSPANRLFRCIVRYGYKEGRWDHFNFENQLLMKVVEFLRHQDGSGGGGGDRM

SAAASGEDEAMSVIPATSSSGGSNQHAFDAGTTTSSCEIDATAGGGGRRKVRFDNDGGGGGEEEEEAAEVKELMEEKEAG

VSYMIGHTCVFAHESSSAVKKFAVNVVYGFLRRNSRRPAVVLGIPHTSLIEVGMAYRV

>OsHAK14

METRSGGSGSASGGGGGGRMRLRKTESAEMRWVVSGGAYEEDEIESSDGGGGTPAAASGSRGGCSDSDDNYEEAEMLRQR

LVRTGPRADSLDVEAQDVAGMNRHQEITVGRSIVLAVQTLGVVFGDVGTSPLYAFDVMFNKYPITSKEDVLGALSLVIYT

LILIPLLKYTLIALWGNDDGEGGTFALYSLICRNARVSLLPNQLRSDTRISSFQLQVPSVELERSLKIKERLETSSMLKK

LLLMLVLFGTSMVIADGVVTPAMSVMSAVNGLKVGISSVNEGEVVMITVAVLIVLFTLQRFGSSKVALAVGPALFIWFCC

LAGIGIYNMKTYGSAVLQAFNPMYIYYYFERNPTQAWMSLGGCLLCATGSEAMFADLCYFSVKSVQLTFVFLVLPCLLLG

YLGQAAFLMENLTENQQVFFLSIPNQAFWPVVFIAILAAIIASRTMTTAIFSTIKQATALGCFPRLKIIHTSRSFMGQIY

IPMMNWFLLVSCLAFVTMFGSINEIGNAYGIAELGVMMMTTVLVTIIMLLIWQINIIVVLCFLTLSLGLELIFFSSVLGS

VADGSWVLLVFAAVLYLIMYIWNYGTKLKYETEVKQKLSMDLLMELGCNLGTVRVPGIGLLYNELARGVPGIFGQFLATM

PAIHSMIIFVCIKWVPVPVVPQNERFLFRRVCPKSYHMFRCIARYGYKDIRKEDYISFQQLLIESLEKFMRREAQERSLE

SDQYDGTDSEEEVASASSRALVGPNGSINSLGVPPAEAAGTTEHPTIGSSMSFDGSLDEAIDGRGSLDDELSFIHKAKES

GVVYLLGHGDIRARKESFFVKKLVINYFYAFLRRNCRRGIAALSIPPSRMMQVAMQYMV

>OsHAK15

MAASSSSSASASAMGGGGMRKAPSMEWRWVSTEEDDEGEEDGDTVEAAAAAVGAVGRGGSFGSEEEEDEEDGGGGGEGEG

EGEDGEKQKLIRTVPSVDWFDVEGYEVSVAQHIEDSEEFDFGRTMFLALQTLAVVFGDIGISPLYTFDVMFSKYPILGEE

DVLGALSLVLYTLISMPLVKYVLVVLWANDDGEGGIFALYSLICRNAKVSLIPNQVHSEKRMSSFRLKLPTPELERSIKV

KEKLESSLLLKKLLLGLVLFGTAMFISNGVITPAMSVLSAVSGLKVGIPNASQGLVVMISVVLLVILYSVQRYATSKMGF

ALGPSLLIWFCCLGGIGIYNLSTYGPAAFKAFNPLYIIYYFGRNPFQAWLSLAGCLLCATGSEAIFANLSYFPVRYVQSM

FALLVLPCLVLAYLGQGAFLIANQNSSEQIFFSSIPSGVFWPVFLIANLAALIASRTMTTAIFQCLKQSIALGCFPRLKI

IHTSRKFMAKIYIPVVNWFLLFSCLGFILLFRSIYDVGNAYAIAELGVMIMATVYVTIIMLLIWETSIVKVLSFVITFLS

LELVFFSSSLSSVGDGGWALIIFASGILMVMFIWNYGSKLKYDSEVKKKLSKDLMRKLGPNLGTIRAPGLGLVYSEIVKG

VPAIFGHFLIALPAIHSIIVFVCIRNVPVPVVPQTERFLFQRVCTRGYHMFRCIARYGYKDKNQESQSTFERLLIEGLEK

FIQREAVELSLQSGDDIDSDEEPPTPSRTIVAPNGSLYSLDVPLLADFVPSAEVIPEASCSTPQHDPVVDYTQNLELELA

FIRQAKQSGAVYLIDNPIVKARKNSWFFKKLIINYFFAFLRNNCRRAMMSMSIPHTNVMQVRLTSYV

>OsHAK16

MAQQQAGARGSKLEIVAARGGSGGSSSAGDAEAPPLDVLRQDSLYRDATRPAHGHHGQESWMRTLRLGFQCVGILHADLG

TSPLYVYQNTFKYGIKHEDDIIGVLSLIIYSFVLFTMVKIVFIALHANDDGDGGTFALYSLISRYAKVCLIPNQQAEDEL

VTRYNDHGKPPATLRRAQWMKSQLEKKPAKIAVFFLTIFATALAISDCVLNPSVSVLSAVNGLKLRAPHLTTDEVVWITV

GILVVFFAVQRFGTDKIGYTFAPVVVVWLLLISGIGIYDLVKYDVGVLRAFNPKYIIDYFRRNKKDGWVQLGEVLLTFTG

TEALFADLGYFSIKSIQLSSTFVLLPSVLCTYIGQAAYLRKHMDQQHIQNAFFNSIPRPLFWPMFVLAIMTSVIGCQAMV

SCAFATMSHLQTLNCFPRIKILHTSRRYSGQLYSPEVNFFLCLLSCVITLSFRTTGFIVKAHEICVVLVMVITTILMTIV

MLLVWKVNIWWIVLFFVVFMSTETVYLSAVLYKFTKGPYMPLAMSAVLMVIMFVWHYVHVKRYKFELEHTVSPNKVRELL

ERRDLKRVPGVGLFYTELVQGIPPIFPHLIEKIPTIHSVIVFISMKHLPIPHVDVSERFLFRQVEPKECMVFRCVARYGY

RDTLEMADDFVTTLVEYLQYYIRDLNLYNTVEPLKMSCPSIRIDSFSWDRRPSGHGIYAEEMLTPIQSFSELTMHPVGMS

SRLAQFQTTKMSLEEMLKIEEDQKLIQREVDNGVVYILGESEVVAKPHSNLLKKVVVNYIFNFLRKNSRKGEKMLSIPRR

KLLKVGITYEI

>OsHAK17

MWFIALLRCVPNRNLKYHLCVLQFILMFSLNSEQDSNVWKDLFLAYKTLGVVFGGLVTSPLYVYPSMNLSSPTEADYLGI

YSIMFWTLTLIGVVKYVCIALNADDHGEGGTFAMYSLLCRHADIGILPSKRVYAEEDPLLHSQSAIARRPSRLGKFFEQS

ITARRVLLFVAVLGMCMLIGDGILTPAISVLSAIDGIRGPFPTVSKPVVEALSAAILIGLFLLQKYGTSKVSFLFSPIMA

AWTFTTPIIGLYSIVHYYPGIFKAISPYYIVHFFLRNKRQGWQLLGGTVLCITGAEAMFADLGHFSKKAIQIAFLSSIYP

SLVLTYAGQTAYLINNVNDFGDGFYKFVPRPVYWPMFVVATLAAIVASQSLISATFSVIKQSVVLDYFPRVKVVHTSQHK

EGEVYSPEINYILMVLCVGVILGFGGGKAIGNAFGVVVIMVMLITTVLLTLVMIIIWRTPLVLAGLYFVPFFIMEGAYVS

AVFTKIPEGGWLPFAVSITLAMIMFGWYYGRQRKFEYEMTNKVSLEHLGELLARPEVQRVPGLCFFYSNIQDGLTPILSH

YIKNMSSLHTVTIFVTLRSLLVAKVDQSKRILINRLGPNGVYGCTVQYGYADNLSLEGGDDLAAQVTSCLQWHIQMDTDG

RRSPEEEMAQLEAARLAGVVHVRGKMRFYVGEDAGWFDKIMLGFYEFLHGICRSALPVLGMPLQQRVEIGMLYKV

>OsHAK18

METRTNEYSRKGAMWELERNLDQPMDAEAGRLRNMYREKTYPTILLLRLAFQSLGVVFGDLGTSPLYVFYNIFPHGIEDT

EQVIGALSLIIYSLTLIPLVKYVFIVLRANDNGQGGTFALYSLLCRHAKINIIPNQHRTDQDLTTYSRRTYEEKSLAAKI

QRWLEGHQFRKNLILILVLFGTCMAVGDGILTPAISVLSATGGIQVEEGRMRNDVVVIISVLILIGLFSMQHYGTDKVSW

LFAPIVFVWFILIGILGAVNICKYDHSVLKAFNPVYVYRYFKRGKTSWTSLGGIMLSITGTEALFADLSYFPVQAIQIAF

TVVVFPCLLLQYTGQAAFIAANTNQVSHAFYISLPAPILWPAFAVATAAAIVASQATISATYSIIKQALALGCFPRVKII

HTSKKYLGQIYSPDINWILMVFCIAVTAGFKNQSQIANAYGTAVIMVMLVTTFLMIPIMLLVWRSHWTLVVAFTVLSLLV

EIPYFSAVVRKIDQGGWVPLVFAAGFMIIMYVWHYGTLKRYEFEMHSKVSMAWILGLGPSLGLVRVPGIGLVYTELASGV

PHIFSHFITNLPAIHSTLVFVCVKYLPVYTVPPDERFLVKRIGPKNFHMFRCVARYGYKDIHKKDDDFEKMLFDSLILFV

RLESMMEEYSDSDEYSTLMMSLPNNPGISNGGVTTTGTNNVMEVMSCTSTHDSIVPVNSRSDDTGSSQVMPASGQMAFQS

VGDEIAFLNACRDAGVVHILGNTVIRARRDSGFVKKIVINYMYAFLRKICRENSAIFNVPHESMLNVGQVFYV

>OsHAK19

MSVQEDGAARPEPDVLRRHDSLYGDAEKVSNNKRHGAGGSWARTLQLAFQSIGVVYGDVGTSPLYVYSSTFPNGIKHPDD

LVGVLSLILYTLILIPMVKYVFIVLYANDNGDGGTFALYSLISRHAKIRMIPNDQTEDANVSNYSIEAPSSQLRRAEWVK

QKLESSNAAKIALFTITILGTSMVMGDGTLTPAISVLSAVSGIREKAPNLTQSQVVWISVAILFVLFSMQRFGTDKVGYT

FAPVISVWFLLIAGIGMYNLTVHEITILRAFNPKYIVDYFRRNGKEAWVSLGGVVLCITGTEAMFADLGHFNIRAIQLSF

TCVLFPSVALCYMGQAAYLRKFPENVGDTFYRSIPAPLFWPVFVVAIMGAIIASQAMLSGAFAILSKALSLGCFPRVEVV

HTSNKYEGQVYIPEVNFLIGAASVAVTLAFQTTANIGNAYGICVVTVFSITTHLMTVVMLLIWKVRLPFIAAFYAAFGLA

EFLYLSSILSKFAEGGYLPFCFSLVLMALMATWHYVHVKRYWYELDRVVPAAETTALLARRDVRRVPGVGLLYSELVQGI

PPVFPRLVDKIPSVHAVFVFMSIKHLPVPRVAPAERFIFRRVVGADAGAGHRLFRCVARYGYTDQLEGAKEFAAFLLDRL

KVFVHEESVFACSRGDNDDDDAMRRAQAMAEEEKRVIDAEAERGVVYLMGEANVTAAAGSSVMKRIVVNYVYTLLRKNLR

EGHKALSVPKDQLLKVGITYEI

>OsHAK20

MSVQEDDDAAGPEVDRLRRHDSFYGDAEKVSNDKSHGTGENWARTLQLAFQSIGVVYGDVGTSPLYVYSSTFPDGVKHPD

DLVGVLSLMLYTLILIPMVKYVFIVLYANDNGDGGTFALYSLISRHAKIRMIPNDQTEDANVSNYSIEAPSSQLRRAEWV

KQKLESSNAAKIALFTITILGTSMVMGDGTLTPAISVLSAVSGIREKAPSLTQLQVVWISVPILIVLFSVQRFGTDKVGY

SFAPVISVWFVLIAGIGAYNLAVHEITILRAFNPMYIIDYFRRNGKEAWVSLGGAVLCITGTEAMFADLGHFNIRAIQLS

FTCVLFPSVALCYMGQAAYLRKFPEDVGDTFYKSLPAPLFWPVFVVAIMAAIIASQAMLSGAFAILSKALPLGCFPRVEV

VHTSNKYEGQVYIPEVNFLIGVASVAITVAFQTTANIGNAYGICVVMVFSITTHLMTVVMLLIWKVRLPFIAAFYVVFTF

TEFLYLSSILSKFAEGGYLPFCFSLVLMALMATWHYVHVKRYWYELDHIVPPDEMAALLARRDVRRVPGVGLLYTELVQG

IPPVFPRLVDKIPSVHAVFVFMSIKHLPIPRVAPAERFIFQRVGPDAGHRIFRCVARYGYTDPLEGAKEFAAFLLDRLKV

FVYEEAVFACQCAEDGGGGGGGDDDGVLRRAEEMAAEEKRLIDAEAERGLVYLMGEANVEAAPGSSLMKQIVVNYVYTRL

RKNLREEHKALSIPKDQLLKVGITYEI

>OsHAK21

MDPGVEKKKQQMELVDVESGGLPVERQDSLFREAVRAEHAGAAHWDEQDSWGRTMSLAFQCVGILYGDIGTSSLYVYSST

FEHGIGHPDDVVGVLSLIVYSFMLFTVIKIVFVALHANDHGDGGTFALYSLISRHAKVSLIPNHQAEDELISGYSSSGKP

SATLRRAHWLKQLLEASKAAKISLFLLTILAIAMVISDAVLTPPISVLSAVGGLREKVPHLTTDQIVWITVAILVVLFAI

QRYGTDKVGYSFAPIILLWLLLIGATGLYNLIKHDISVLRAFNPKYIIDYFRRNKKEGWVSLGSILLCFTGSEALFANLG

YFSIRSIQLSFSFALLPSVLLTYIGQAAFLSKNPKNVANTFFAATPISLFWPTFIMAIAASIIGSQAMISCAFATVSHLQ

SLSCFPRVKILHTSKRFPGQLYIPGVNFLLCVAACVVTVSFKTTVIIGKAHEICVILVMIITTLLMTIVMLLVWKINILW

VALFFITFTSTEAVYLSSVLYKFTHGPYVPVAMSVVLMVVMIVWHYVHVKRYKYELEHTVSTDKVKEMLESHDLKRVRGV

ALFYTELVQGIPPIFPHLIEKIPTIHSVLVFISIKHLPVPHVDTSERFLFRQVELKDYKVFRCVARYGYRDSLEEAKDFV

VTLLENLQDYIRDVNLYTDEPHTISAHSSCNHSFSREKPSGRYAVHAEDMLTPIESFSEITALSNYGSDRLPHFKASKMN

MEELAKIEQEQMFIEKEMEKGVVYILGETEVVVRPHSSLLKKIVVNYVYSFLRKNFVQGQKMLFIPHRQLLKVGISYEI

>OsHAK22

MAQQQGQGAGTTTVAMMSRNPSYYYSGEGELSLAVQRQDSLYRDASRAGQHEQAHGEGWARTLRLAFQCFGVLYGDIGTS

PLYVYSTTFDGGIRHTDDLLGVLSLIIYSFLLFTIIKYVYIALRANDDGDGGTFALYSLISRHAKVSLVPNQQAEDELHL

HISKSSSLRRPSVQRLASTAEERAQWVKDLLENSRPVRISLFLLTILATAMVISDACLTPAISVLSAVGGLKDKAPHLNT

EQVVWVTVGILVMLFAVQRFGTDKVGYLFAPVVLLWLLLIGGVGVYNLAAHDVGVLRAFNPKYILDYFRRNGRHGWVSLG

GVLLCFTGTEALFADLGCFSIRSIQLSFAFGLVPAVLLAYAGQAAYLRVYPDHVGDAFYASTPQVLFWPTLVLALAASVV

GSQAMISCAFATISHSQAMGCFPRVKVVHTSRQYQGQVYIPEINLLLGAAACVVTVAARDTVVIGEAHGICVVLVMLITT

LLLTVVMVLVWRVNIGWVLVFACVFASTESVYLTSVLYKFAHGGYIPVAMSAVLMGVMGVWHYVHVRRYKYEMERTVSTE

RVRELVSRRELQRVPGVGLFYTDLVQGIPPVFPHLIDKIPSIHTVLLFVSVKHLPVPHVDPSERFLFRQVEPQEHKLFRC

VARYGYRDRLEDARDFVANLVERLQYYVRDVNLYGAAANNKVSYPSSRCDSMGIPKSASYAERLQLQRARSVAMLHSHSQ

HQQQPLPQQLGQLLQYSASTGEQQRRSVYAEEMLTPAESFSEMGTMAASGRQLMAVAVKMSLEEMARIEEEQRFIQREME

KGVVFILGESEVVARPHSSLLKKLVVNYAYSFLRRNCRQGDKMLAIPRSQLLKVGMSYEI

>OsHAK23

MDDDDSGIQEEPAPPPPPPPPPPPPPPLRRLLTATRSGGSRWVDGSEVGSSESAPWSLDGDRSLRLSVDSAASAGGASGG

GGGGGPLSRASSGAFRRRFGKQPRRVDSLDVEAMSVRGAHGHSSKEISMLSTVAMAFQTLGVVYGDMGTSPLYVFSDVFS

KVPIKSEVEILGALSLVMYTIALIPFAKYVFIVLKANDNGEGGTFALYSLICRYAKVSLLPNQQRVDEDISSFRLKLPTP

ELERALSVKESLEKNPVFKNILLFLVLMGTSMVIGDGILTPSMSVMSAVSGLQGRVPGFGTDAVVIVSILFLVLLFSVQR

FGTGKVGFMFAPILALWFINLGTIGIYNLAKYDISVVRAFNPVYIYLFFQTNGIKAWSALGGCVLCITGAEAMFADLGHF

SVKSIQVAFTAVVFPCLLIAYMGQAAYLMKYPFAVERIFYDSVPEILFWPVFVIATLAAMIASQAMISATFSCIKQAMAL

GCFPRIKIIHTSKKVMGQIYIPVMNWFLMVMCIIIVATFRSTNDIANAYGIAEVGVMMVSTALVTLVMLLIWQTNLFLVM

CFPVIFGSVEFVYLTAVLSKIQEGGWLPLAFSSLFLCIMYTWNYGSVLKYQSEMRGKISLDFILDLGSTLGTVRVPGIGL

VYNELVQGIPSIFGHLLVTLPAMHSTIVFVCIKYVPVPYVPFEERFLFRRIGQKDYHMFRCVARYGYKDVRKEEHGFFEQ

LLVETLEKFLRKESQEMALEASAMAVERDDVSVVSDIPSSPVEAGDLHVPLLSDQRLGDGTQTFITEGNTPVLPTSSISE

EDPSLEYELESLREAIASGFTYLLAHGDVRARKESFFTKKFIINYFYAFLRRNCRAGTATLKVPHSNIMRVGMTYMV

>OsHAK24

MDVEGGGAAARRKGGWWWWREEAVLAYQSLGVVYGEVAAAPLYVYRSAFAGGDIEHSAGNEEIYGALSLVFWTLTLVPLA

KYVLLVLRADDAGEGGTFALYSLICRRVRAGLLPPCAAAAAGEELDAAGAAAAPVSAVRAALERHRVLQRLLLLLALLGT

CMVIGDGVLTPAVSVFSAVSGLELSMDKDQHKYILLPITCVILVCLFALQHYGTHRVGFLFAPIVCLWLLCISIIGVYNI

IHWNPHVYQALSPYYMYKFLRKTQTGGWMSLGGILLCVTGSEAMYADLGHFTQNSIKMAFTLLVYPALVLAYMGQAAYIS

RHHNFEDGSHIGFYVSVPEKIRWPVLGIAILASVVGSQAIITGTFSIIKQCSSLNCFPRVKIVHTSSTVHGQIYIPEINW

ILMILCLSVTIGFRDTKHLTNAQGLAVITVMLVTTCLMSLVILLCWNKSIVYALSFLLFFGAIEVIYFAASLVKFHEGAW

VPVTLSFIFMMVMCVWHYGTKKKYEFDVQNKVSISWLLNIGPSLGIVRVRGIGLIHTELMSGIPAIFSHFVTNLPAFHQV

LVFLCIKSVSVPHVQPEERFLVGRIGPKKYRIYRVIVRYGYRDVQKDDVEFEKDLVSSIAEFIRCADSNQNSFMDGASHS

CEGLSFISKGLPLEEEEGEFDGSDSTGSSAHKEINPNTTAPKPKRVRFALPKDTKIDREVRGELQELMEAREAGMSFITG

RSHMKAKSGSGLIKQIVINFGYEFLRRNSRGPAFAVNLPHVSTVEVGMICLV

>OsHAK25

MDLEAAHGAAAAPGKRRRRARESWGASLLLAYQSLGVVYGDVATSPLYVYKSAFAGDDIQHSAGNEEIYGVLSFVFWTLT

LISLVKYVLIVLRADDGGEGGTFALYSLICRHVRAGLLPGGGGGAGDELAVGGRRDARAMSRLRAMLERYRVLQRLLLLF

ALLGTCMVIGDGVLTPAVSVYSAVSGLELSMEHEHHKYVQLPVTCAILIGLFALQHYGTHRVGFIFAPIVCVWLLCISAI

GVYNIVHWNHHVYRALSPYYMYQFLKKTQTGGWMSLGGILLCVTGSEAMYADLGHFSQSSIKIAFMSVVYPALVLAYMGQ

AAYISQHHSFENAYHIGFYVSVPEKLRWPVLVIAILAAVVGSQAVITGTFSIIKQCSSLSCFPGVKIVHTSSTVHGQIYI

PEINWILMILCLAVTLGFRNTKHLANAQGLAVITVMLVTTCLMSLVIVLCWNKSIFLALGFLIFFGTIEVLYFSASLVKF

HEGAWVPITLSFIFMIVMCVWHYGTIKKYEFDFQNKVSVNWLLNLGPSLGIVRVRGIGLIHTELVSGIPAIFSHFVTNLP

AFHQVLVFLCVKSVPVPHVQPEERFLVGRIGPKEYRLYRVIVRYGYRDVQKDDIEFEKDLVSSIAEFIRSGDSHHNGVLE

DTDKSCEKLSSISNGIPLWMEDGEVDASASPHKETDTQIISPNRKKARFVLPKNAQVDSEVRRELQELMDAREAGMSFIL

GHSYMKAKSGSSFIKRIVINFFYEFLRRNSRGPSYAATIPHASTLEVGMVYQV

>OsHAK26

MEYHHRPHSPPPSDDDVVVIQMNAAAIAAVDEWSSTNEVDDAAAGKGGGLTRRTFSQAYKMKHRTPLEFTWRQVALLSFQ

SLGVVYGDLGTSPLYVFSSISLDDPGEADFVGILSIILWTFTMICLVKYVFIVLKADDHGEGGTFALYSLLRQHVNFKGN

MPVPVTHLASDINLKFHSKKRILTSKLLKFLEQSTKWQAVITYIVLAGTCMVLGDGALTPAISVLSAVQGIQSRSSSITQ

AHVVLLSVIILFILFFFQKHGTSKVSFTFSPIMILWFTFVAFIGLYNIIKHYPPILKAVSPHYIIIYFIRNKRAAWETLG

AIVLCITGAEAMFADLGHFNKSSIQMAFSVIVYPSMILAYAGQAAFLVKNPSKLSTTFYSSTPEPLFWPMFIIATLAAIV

ASQALISASFSIIRQSIALGCFPRVTMKHTSGKHEGQVYSPEINYFLMVACILITVGFKGGPEIGQAFGVAVIFVMLFTT

NLMTVVMLIIWESNIALASLFFVFFFSIEGIYMTSLMNKILQGGWVPFAITAFFLIITLSWTYGRSKKGEYELANVMERE

EFIKTVTTRSRVPGVCIFCTDMMNGIPPIVRHYVQHVASLRELMVFVTIRVLPVRTVLPEERFIIDKLEPVGVYRCIVQY

GYMDNHNMEGDDYVASVIASLKEIAENDDEILVLDSALINGSTFVLGRTIIKMGTRHNCLKRFFINNLYRFLQKNFRSNM

SSLKINPGKTLQVGMLYEI

>OsHAK27

MGDDVLGRGSRRDQEIVLVDIVDDDDHDDVPAVRRQDSLYVDATRAGGANHRGGQEESWARTLKLAFQCVGILYGDIGTS

PLFVYSSTFKDGVRHPDDLLGALSLIIYSFALFTIVKYVFIALRANDDGDGGTFALYTLISRHAKVSLIPNQQAEDELIS

KYNTGKPQATLRRARWMKELLETNRAVKIWLFLLTILATAMVISDAVLTPAISVLSAVGGLKEKAPNLTTDEIVWITVAT

LVVLFAIQRFGTDKIGYLFAPIILLWLLLIGCVGIYNTIKFDTGVLRAFNLKYIIDYFRRNKKDGWISLSGILLCFTGTE

ALFSDLGYFSIRSIQLSFSFGLVPSVLLAYIGQAAYLREHPEHIANTFYRSTPNVMFWPTFILAVAASIIGSQAMISCAF

ATISHLQTLNCFPRVKILHTSRQYSGQLYIPEVNFLLCVGACLVTIGFKTTVIIGEAHAICVVFVMIITTLLLTIVMLLV

WKVSIWYVALFFIVFMSSESIYLSAVLYQFVHGEYVPVAMSVFLMIVMTVWHYVHVKRYEFELEHTVPRDKVKELLERRD

IQRVPGVGLFYTDLVQGIPPVFPHLIEKIPSIHSVLIFVSIKHLPIPSVDRSERFIFRHVDKEEYKVFQCVARYGYRDPM

EEAKDFVDALTENLQYYIRDVNFYTTGGDQHIFRSTSYASSIAESFASYEKHSGHAVYAEEMLTPAESFSEHTKQLSGRS

KHFKQFQVENMNMQKMEKVQQEQQAILREMENGVVYILGESDIVASPHSSLLNKIIVNYIYSFLRKNCRNGEKMLSIPRS

QVLKVGIAYEI

>AmtHAK1

MDLEIGLSNTNNLMSSWSSLRSIAMLAYQSVGVVYGDMSISPLYVYSSSLSRLRGGVDMEEEEILGMLSFILWTFSLIPL

LKYVFFVLNADDEGEGGLFALYSLLCKNANLRVPKDSVKPHFRGGVDKSFFERHAWARVSVLLIVMLGTGMVIVDGVLTP

AISVLSAVSGIRVKANLHEGLVLAIACLVLVGLFALQHFGTRKVAFMFAPVVILWLLSIGAIGLYNVVSWNPRVLRAISP

CYMFHFLRMTGKDGVVSLGGILLCISGAEAMHADLGHFSRFSIKVAFLGLVYPCLVLAYMGEAAFLTKHPESIHMSFYNS

IPESVFWPAFLTATLASVVGSQAVISATFSVVQQCAALDCFPRVRVVHTSKLIYGQIYIPEVNWILMLLCLAVTLGFQDT

LSIGHAYGIN*

>AmtHAK2

MEVGAHQTVVTVEMAENNEVVVSESQMQIESKSSSTGLRREGFKRLDSLDKEANNLSGLSVSKALSIMGVLKLAFQSIGV

VYGDIGTSPLYVFESTFAEARSPPTREHIVGALSLIIYSLTLFPLIKYIFIVLWANDNGDGGTFAMYSLICRYCRVSATT

NEQAEDITAYKEGHKKRMNGRAKRIKEAIEGSGFAKLLLLCIALFGTCMVIGDGILTPCISVLSAVEGVKKMDASISQEA

VVTLSVAILVVLFSVQRLGSDKVGYTFAPAILVWYSFITVIGVYNILHHDFTVLKAFNPLCILQYFTSASSNPKQAWISL

GGIVLCMTGTEAMFADLGHFSVRSIQIAFAGLVYPCLICAYVGQAAYLSHRPDHIGDAFYKSTPDSVYWPMFVVAVLASI

IASQAMISATFAIIKQSMALGCFPRVRIVHTSRSHEGQVYIPEINFLLMLACVMVTASFKDTANIGNAYGIAVVAVMLVT

TALLTLIMLMVWQTSLILVALFVAIFGSLELIYFSSVLYKFDKGGYLPLSFAAALFLVMYVWYYVQTKRYAYEFEHKLSA

EQMTTNPGLTRVPGIGLLYTELTHGVPSIFSHFLTNLPAMHSVVVLVSVKYLPINKVPVSERFLFRRVGPKACNMYRCIA

RYGYRDRRVGNQEFERFLMDHLKAFIRTEGWEKADIDGQVSERNEVEVSIGELAIDHVVPKTQCGNTQSTEGGEDNCEER

SNEIIREEDAEEEIRFLERSRESGVVYLLGHSEVRASKESGLVKKMVVDYVYDFLRRNSRQGLVALQIPHKNLLQVGMNY

YI*

>AmtHAK3

MDIEGGLYANSVKKESWKTVLTLAYQSLGVVYGDLSTSPLYVYKSTFAEDIRHSETNEEIYGVLSFVFWTLTLVPLLKYV

FIVLKADDNGEGGTFALYSLLCRHARVSFLPNCQVADEALSTYKKDSLPETSIGSRVKLLLEKHKVLQRVLLILALIGTC

MVIGDGVLTPAISVFSAVSGLELSMSKEHHKYAEVPIACIILVGLFALQHYGTHRVGFMFAPIVITWLLCISIIGVYNIF

HWNPHVYQALSPYYMYKFLKKTQRGGWMSLGGILLCITGSEAMFADLGHFSQLSIQIAFTFVVYPSLILAYMGQAAYLSM

HHVIESHYHIGFYVSVPEKIRWPVLAIAILAAVVGSQAIITGTFSIIKQCSNMACFPRVKIVHTSDKIHGQIYIPEINWI

LMLLCLAVTIGFRDTKRMGNASGLAVITVMLVTTCLMSLVIVLCWHKTILLAICFIFFFGSIEALYFSASLIKFREGAWV

PVALSLAFMGVMLVWHYATLKKYEFDMQNKVSMQWLLGLGPSLGIVRVPGIGLVHTELVSGVPAIFSHFLANLPAFHQVL

VFVCVKSVPVPYVRPGERFLVGRVGPKDYHLYRCIVRYGYLDARKDDVEFENELVYSIAEFIRGSGGGLQNVGDELGENS

DAMTVVGSPGPESEIMRACDGGSGPAEPTGSTEIWSPRLGPGPVPRKRVRFVLPESPTMEAGVRAELNELMEAREAGMAF

ILGHSYVRAKPGSSLLKKVVINVGYDFLRRNCRGPAAALSIPHTSTLEVGMIYHV*

>AmtHAK4

MDPETPVIDGGLALQNRKGTSCKAILMLAYQSLGVVYGDLSTSPLYVYKSTFSGKLSLREDDEEILGVLSFIFWTFTLIP

LFKYIFIVLSADDNGEGGTFALYSLLCRHARLCILPNKQAADEKLSAYKMQDGVETWESSMLKSFFEKHPRFRLGLLMIV

LLGTCMVIGDGILTPTISVLSAVSGVKVKITELHENYIVAISCLILVGLFSLQHHGTHRVAFMFAPIVTAWLLCISGIGI

YNIFRWNTSVFHALSPSYMFKFLKATGKEGWVSLGGVILCITGAEAMFANLGHFSQLSIKVAFTLLVYPCLILAYMGEAA

YLSKHHEDIQRSFYKAIPEAVFWPVFIVATLAAVVGSQAVISATFSMISQCCALSCFPRVKIVHTSSEIYGQIYIPEINW

ILMCLCLAITVGIRDTNTIGHAYGLAVVTVMFVTTCLMSLVIVVVWKQKVIVALLFLSFFGFIELLYLSASLIKVHEGGW

IPLSLSLIFMGIMYVWHYGTIKKHEFDLENKVSMKRLLSLGPSLGMVRVPGIGLLYTDLVTGVPAVFGHFVTNLPAFHQV

LVFVCIKSVHVPHVLEKERFLVSRIGPKEFQMFRCTVRYGYKDLQQENYDFENRLVSAIFKLVQMEERELGPILKPETSS

QMRISPGSDSLNMHMQRLTHPNQEENMQSSCSMDIQVMMPEMGNSEIWDGLDELGKEPVIGQECLEIMKAREAGVAYILG

HSYAKAKKSSSLLKKLAIDVVYAFFSKNCRGPDVALNVPHVSLLEVGMIYYV*

>AmtHAK5

MKDSWRSLLLLAYQSLGVVYGDLSISPLYVYKSTFADDITHSETNEEIFGVLSFVFWTLTLIPLFKYVFIVLRADDNGEG

GTFALYSLICRHAKVSLLPNRQVADEELSTYIMESPPDKRNGSRVKIWLEKHKNFHTALLIVVLLGTCMVIGDGVLTPAI

SVFSAVSGLELSMSKEHREYAVVPITCFILVCLFALQHYGTHRVGFFFAPVVLTWLLCISTLGLYNIFHWNPYVYQALSP

YYMYKFLKKTRKGGWMSLGGILLCITGSEAMFADLGHFSYRAIQMAFTFVVYPALILAYMGQAAYLSKHHHIDTSYQIGF

YVSVPESVRWPILAIAILASVVGSQAIISGTFSIINQSQSLSCFPRVKVVHTSDKIHGQIYIPEVNWMLMVLTLAVAVGF

RDTKHMGNASGLAVMTVMLVTTCLTSLVILLCWHKPPLVALAFLLFFGPLELLYFSASLTKFLDGAWVPILLALILMTLM

FVWHYATIKSYEYDLHNKVSLDWLLALGPALGIARVPGIGLVLTDLVSGVPANFSRFVSNLPAFHRVLVFVCVKSVPVPR

VPAAERYLIGRVGPPGARLYRCISRYGYRDVRRDTHDFERELAARLADFVRLTSSPQSPASLLQSETSLERGSGDEMVKM

TVVGSAPDFLASCCVYETDESPQSVSLQESDFRASENGFRRRVRFVFPDSPRMDDSVREELEELATAQQAGTAFIMGHAH

VKASPGSSFLKRLAIDYGYTFLSRNCRGPAVALRVPHVSLLEVGMVYVV*

>AmtHAK6

MDQEMGSTSARPRGQVSWHDYCKNVLLLAYQSFGVVYGDLSTSPLYVFKSTFVGKLQNHENEDAIFGAFSLIFWTFTLIP

LFKYVFILLSADDNGEGGTFALYSLLCRHAKLSLLPNQQAADEELSTYKYGHSSQGIVSSPLKRFLEKHKRLRIGLLLIV

LFGACMVIGDGVLTPAISVLSAISGLRVCAEKLHDREMVIIACVVLIGLFALQHFGTHRVGFMFAPIVIIWLFCISAIGI

YNIIYWNPKIYHALSPYYVYKFFKETGKEGWISLGGVLLCITGTEAMFADLGHFTAASIRVAFAGVIYPCLVLQYMGQAA

FLSKNISDIEYSFYNSIPKPVFWPVFVIATLAAIVGSQAVISATFSIIKQCHSLGCFPRVKIVHTSKQIYGQIYIPEINW

ILMVLCLAITIGFRDTTLIGNAYGIACITVMFVTTWLMALVIIFVWQKSILVALSFLLVFGSIEAMYLSASVMKVAQGGW

VPIVLAFVFMLVMYVWHYGTRRKYLFDLQNKVSMKWILTLGPSLGIVRVPGIGLIYTELVTGVPAIFSHFITNLPAFHQI

LVFVCVKSVPVPYVTPDERYLIGRIGPKAYRMYRCIVRYGYKDVRKDNDDFENHLILSLAEFIQMEAEESQSSSYEGSTD

GRMAVISTPVRPGLRLVESENEGDDSILSLRSSKSSTLQSLQAMYEQESPQAMRRRRVRFELPKSPTVDATVREELMELI

EAKQAGVAYIMGHSYIKARRTSSFLKKIAIDIGYAFLRKNCRGPAVALNIPHISLIEVGMIYYV*

>AmtHAK7

MADFVQKDGSENGEERADVQSPPKEIKERKLSWARLRRVDSLDFEAGRVSGATGHGSRKGWLTALNLALQSIGVVYGDIG

TSPLYVYSSTFTRGIDDQEDILGVLSLIIYTIVLITFIKYVFIVLFANDNGDGGTFALYTLICKHAPKVGLIPNIQQEDR

EISNYKLDKPSSHLRRAQKVKEFLEKNKAAKTTLFLVTMIGTSMVIGDGILTPCISVLSAVGGIKTAASSLNQDAVVAIS

AVILILLFAVQKYGTDKVGYAFAPIVLIWFSFIGITGIYNLFKYDVGVLHAFNPQYIVKYFKRTGKQGWISLGGVLLCIT

GTEAMFADLGHFSVKAIQLSFTAIVFPALLCAYIGQASYLRKFPDHVGDTFYKSIPGPIYWPTFVVANAAAIIASQAMIS

GAFAVIAQCLPLGCFPRVKVIHTSAKYEGQVYIPELNYFLMAACVIVTVAFRTTNNIGNAYGIAVIAVMVKTTFVVTVIM

LMIWKISIWLVALFFVVFGVVELVYLSSALYKFKQGGYLPLAFSLAFTMIMITWHYVHKKRYQFELENKVSDEYMSSLAN

NPNLIRIPGMGLLYSELVHGIPPIFSHFISSIPSIHSVLIFVTIKSIPVSKVALEERFLFRQVEPKEYRMFRCVVRYGYN

DVMDEPKVFQSLLVEYLKEFVRQQDFFLETETTDQSNANQEAKESKQRASGSSTVYDERCLQQSSGPIQAVTSARSRNSS

SQIISGPIQAPQEEAQFLQKALDDGVVYLLGEAEVTAKAGSSLLKKFAVNYAYKFIRKNLRDGEKVMGVPRSRMLRVGMT

YEI*

>AmtHAK8

MASDSEDLEAGETNVTDKAVGRTLSFSNDYKPHPRKPKEFASLQHLFLAYQSLGVVYGDIGTSPLYVFSSVSLSNPGEKD

ILGIFSLIFWTLTMIGLLKYVFIVLHADDHGEGGTFALYSLLNQHINFKNRRISGQTQRLDTDSNLKYYSGGSLQSKMAK

KLLEKSSMAQSFLTFIVLLGTCMVIGDGALTPAISVLSAVQGIQLRSPKIDTNDVVLLSVVILVLLFLFQRFGTSKVSIS

FSPIMLLWFGSTAAIGVYNIVTYYPMVFKALSPHYIYYFFTRNHKHGWEMLGGAVLCITGAEAMFADLGHFNKRGIQIAF

SCIVYPCLLLTYGGEAAYLIKNPQNLSTTFYSSVPSPVFWPMFVIGTLAAIVASQALISASFSIIRQSMALGCFPRVNMF

HTSSKHEGQVYSPEVNYFLMVICILIVVGFKGGVEIGNAYGVAVIWVMLITTCLMTVVMLVIWDTNIMLISIFFFVFVSI

EGVYMTSLLNKVPQGGWVPFAVSAFFLIIMLSWTYGRSKKHEYEVEHKLSLVDLQELASNLNSSRVSGVCFFCTDLMNGI

PPIIRHYVQNVGSLHQIMVLVTVRILPITTVLPEERFLIGKLGPKGVYRCLVQYGYMDHPNMEGDEYVATVLEKLKDLAE

SHEELQLLESGKDRAVTFVMGRTILRTSEKTGWFQQLVIGWCQRLVIDGIYRFLQNNFRSSISTLRIPPSKTLQVGMQYE

I*

>AmtHAK9

MERELEPRGSVTSLPHCEIHVNGASDCLIEDGVDPNPRISALASHGHAPQLAHENEDAGKWRTVVLAYKTLGVVFGGLVT

SPLYVYPSMRLTSPSKADYLGIYCIIFWTLTLIGVVKYALIALKANDHGEGGTFALYSLLCRHADVGSISSRKSNSSMRL

SHCGIYATPDKSYLVKFIEQSVIARRLLLFVAMLGVCMLIGDGILTPAISVLSAMDGLKGPFHSISQSAIEALSAGVLII

LFLVQKFGTSRVSFLFSPIMAAWTFSTPIIGVYSILKYYPSIFKALSPHYAVLFFLRNGKKAWLLLSGIVLCITGSEAMF

ADLGHFSQRSIQIAFLFTIYPSLVLTYAGQTAYLIINPNDHSDGFYKFVPKPIYWPMFVVSTLAATVASQSLISATFSVI

KQAVVLDYFPRLTVKHTSRSKEGEVYSPEVNYILMVLCIAVVLGFRDGQDLGNAFGVVVILVMLITTILLTLVMIIIWKT

PLPLVALYFIPFFILEGVYVSSVFTKIPEGGWLPFAISIFLVLIMFTWYYGRQKKTEYEMTNKTTLDGLSKLLSHPGVNR

VPGLCFFYSNIPTGLPQTLEHYVKNMRSLHRVIIITTFKYLMVAKVAGGERIIIKRLGLRGVYRCIVQYGYADCLNPEGD

DINNRVINCLRTYLEKSSAESPELLQEEISELDHARKLEVVHVLGKTRFQISKRTRWFDKVFIGFYKVLHENCRSALPSL

GIPLNQRVEVGMLCEA*

>AmtHAK10

MQVRSKIYYKNILLLAYQSFGVVYGDLSTSPLYVFKSTFSDRLHKFHDEDAIFGAFSLIFWTLTLIPLFKYVFIVLSADD

NGEGGTFALYSLLCRHAKFSLLPNQQAADEELSTYYRPGYSSGIVGASSLKRFLEKHKKSRTGLLVVVLFGACMVIGDGV

LTPAISVLSSISGLQVRTHKLDNGVVVVISCVVLVGLFALQHHGTHKVAFMFAPIVIVWLLCIGTIGIYNVVVWNPRIFR

ALSPHYIYKFFKATGKDGWISLGGVVLCITGTEAMFADLGHFTNISIRVAFVGLIYPSLVLQYMGQAAFLSKNFSDIHTS

FYKSIPESVFWPVFVVATLAAIVGSQAVISATFSIVKQCHALGCFPRVKVVHTSKRIYGQIYIPEINWILMVLCLAITIG

FQDITVIGHAYGIACMTVMFITTALMALVIIFVWQRSIFWAFIFLLFFGCIECFYLSASIIKVPQGGWVPLVLSAVFMAV

MYIWHYGTRRKYLFDVQNKVSMKWILTLGPSLGIVRVPGIGLIYTELVTGVPAIFSHFVTNLPAFHQVLVFICVKSVPVP

YVPAEERYLVGRIGPKAYRMYRCIVRYGYKDVPKDDDVFENQLILSIAEFIQMEAEETQSEGPASSSGLLDGRMAVIRAP

VGPGTRLLMSEDDGTDEVAGEATSTIRSSKSVTLRGLQALYEQDSPSVSRRRRVRFELPECHNLDPNVRDELLELVEAKH

SGVAYIMGHSYIKARRTSSFIKKFAIDVAYSFLRKNCRGPAVALNIPHISLIEVGMIYYV*

>AmtHAK12

MGEPVIAVEEQPISTEIQGKSPIHGRLKRADSFDAESPGLKRGSQTHSWGAIFNLAFQSIGVVYGDIGTSPLYVYSSTFA

SSIQHNDDILGVLAIIIYTLILITLIKYVFIVLQANDNGDGGTFALYSLICRYAKVSLIPNQQAEDRDVSNYRLEPASRS

LARASKVKSWLEGSATAKFTLLLMTMLGTSMVIGDGVLTPCISVLSAVGGIKGAASSLTEGRIVSISVAILVCIFLVQRF

GTDKVGYSFAPIILVWFLFIGVIGFYNFCKYDPGVIKAFNPKYIIDYFSRNKKDAWISLGGIVLCITGTEAMFADLGHFT

VRSIQISMCSVVFPSLILAYTGQASYLRKHNEHVKDTFYKSIPTPLYWPMFVVAVGAAIIASQAMISGTFSIVQQSLAMG

CFPRVKVVHTSSKYEGQVFIPEVNYLLMIACVLVTAGFRTTEKIGNAYGIAVVFVMTLTTALVTLVMVMIWKTNIFLILA

FVLVLGSVELLYLTSVLYKFTQGGYLPLAFAAFLMTIMSVWSMVHLRKYNYELQHKVPTRNLLDLTTKPEVHRVPGIGLF

YSELVEGVPPIFSHYIDNVPAVHKVLVFVSMKWLPIGRVPQDERFLFRRVGPRELGVFRCIVRYGYTDAALVHGEFEAGI

VEGLKQYINSEHMWSMGEDGEVVAREFDDKEEVERELREVEKAMSTAVVYLVGQTEVVARSEARWWERVLIDYLYTWLRR

NVRQGVEVLRIPNRRMVKVGMTYEL*

>AmtHAK13

MEEGSEDSYRLLSSESRWVDGSEVDSESPPWSVEDQVLTNEELGTVRRRLTKKPKRLDSLDVEAMEISDAHGHHSKEVLS

WHTLALAFQTLGVVYGDLGTSPLYVFSDVFSKVPINGDDDVLGALSLVMYTIALLPFAKYVFIVLKANDSGEGGTFALYS

LICRYAKVNLLPNRQPADEHISSFKLRLPTPELERALNIKEKLENKPSLKTLLLLLVLMGTSMIIGDGILTPAMSVMSAV

SGLQGEIPGFDADAVVIVSIIILVLLFSIQRFGTGKVGFTFAPALALWFFCLGSIGVYNIVKYDITILRAFNPAYIYIFF

KRNSRKAWSALGGCVLCITGAEAMFADLGHFTVLSIQIAFTCVVFPCLLLAYMGQAAYLIKHPLSAERIFYDCVPDGFFW

PVFVIATLAAMIASQAMISATFSCIKQSMALGCFPRLKIVHTSKKFMGQIYIPVINWFLMIMCIVVVASFRNTTDIANAY

GIAEVGVMLVSTTLVTLVMLLIWQTNLFLALCFPIIFGTVELIYLSAVLTKIKEGGWLPLAFASCFLCIMYTWNYGSVLK

YQSEVRDKISMDFMLDLGSTLGTVRVPGLGLLYNELVQGIPSIFGQFLVSLPAVHSTVIFVCIKYVPVPVVPQEERFLFR

RVCPKDYHMFRCIARYGYKDVRKEDHLSFEQLLMESLEKFLRREAQELALESGRVEIDLESESSVGSHDIRPRSEGNELW

VPLMGTSGFDGGSSVAEDYGLEVGSSIARVGLGHGFGSGPSLPTTVIPTLDEDPGLEYELSALKEARDSGITYLLAHGDV

RARKDSWFFKKLVINYFYSFMRKNCRAGTANMTVPHMNIIQVGMTYMV*

>AmtHAK14

MDPSIENEGSDKGSMWVLDQNLDQPMDEEAGRLRSMYREKKFSTIFVLRLAFQSLGVVYGDLGTSPLYVFYSTFPHGIND

PEDVIGALSLIIYSLTLIPLLKYVFLVLRANDNGQGGTFALYSLLCRHAKIKTIPNQHRTDENLTTYSRHVYDENSLAAK

TKAWLEAQFYRKNVLLIVVLVGTCMVIGDGILTPAISVLSAAGGIKVDHPKMSNDVVVVVSVVILVGLFSVQHYGTDRVG

WLFAPVVLIWFISIGGIGAYNIWNYDRTVLKAFSPLYIYRYFRRRKAQSWMSLGGIMLCITGTEALFADLAHFSALAVQI

AFTVVVFPCLLLAYTGQAAYLMQNQERVVDVFYRSIPESIYWPMFLIATAAAIVASQATISATFSIIKQSLALGCFPRVK

VVHTSKKFLGQIYIPDINWVLMILCIIVTAGFRNTSQIGNAYGTAVVIVMLVTTFLMTLIMLLVWRSHWVLVLLFTVLSL

AVEGTYLTAVLIKVDQGGWVPLVIAAAFLLIMYVWHYGTVKRYEFEMHSRVSMAWIVGLGPSLGLVRVPGIGLVYTELAS

GVPHIFSHFITNLPAIHSVVVFVCVKYLPVYTVPQEERFLVKRIGPKNFHMFRCVARYGYKDLHKKDDDFEKMLLDSLFM

FVRMESMMEGCSDSEYSICLQQTQKSTDFLYSENSSNLVSSNIEMVQSSTDSIVADRSPLRTSKSLTISSGRIEGGVDEF

EYLKSCKEAGVVHILGNTIVRTRRDSGFIKRVAVDYIYAFLRRICRENSVIFNVPHESLLNVGQVFYV*

>AmtHAK15

MENELNSLFERPKKETWQNTLSLSFQSLGVVYGHLSVAPLYVFGSMSLSPKEIISEEELYGLLSFIFWTLTLIPLLKYIF

IVLQADDNGEGGTFALYSLLCRHAKVGLLPNYQISNHELLTDDMGSFSEMNMESKARKVFQSHTTFHYLLLSMALLGSCM

VIEDGALNPALSVFSASSGLEQSVLNVPTGSVAVPLTCVILIGLFVLQPYGTHKIGSMFAPIVIIWLLFIGGLGLYNIFC

WNHLVFHALCPKYMYNFMKTLDLKNWGSLSSILLCITGSEAMFADLGHFSQKSIKIGFGFMVYPSLVLCYLGQTAYLSRN

LEKFGTHFISCVPRCIHGPFTVLAILASIVGSQAIITGTFSVVNQCLALGCFPRVKVIHTSKNIRGRVYIPEVNWILMAL

CLISLVAFRDIEDLGNAIGLVVITGMLITTCLMPLIITLLWKKGIFIAACFAISFGSIEALYFSACLSNFLKGAWIIVIL

SLILMFTMISWHYGSTKKYQYDLENKVSIKWLTSLGPSLGAVRVPGIGFIYTDLVTGIPAFFSHFIINIPAYHQVLVFVS

FVSVPVPYIPPDKRHLIGRIYRKEYRVYRCIIRYGYRDNVKDSNDFEDQLIFKLGEFISTEDMGLELNSHVEGRVTVVGD

QKQIKAGLIPIDEADLNLGDPSFVDRENGDTQLRDDQLQSAIVPVKRKKVRFVLPPESPEMPASVVEELQELLDARDSGT

AYLLGHSRFVVRNGSNLLKKLLILVYIFLERNCRGPLVALDIPHVALLKVGTLYTI*

>AmtHAK16

MAEESANGGLVSMDSSESRWVYGDEEEEEDDDDDAEHEGSSNWMSGDSEEENVEQRLIRTGPRIDSFDVEAFEVPGAPRT

DYEDFTIGRALLLAFQTLGVVYGDVGTSPLYTFSVMFNKCPIHGEEDVLGALSLVLYTLILISLIKYVLVVLLANDDGEG

GTFALYSLICRHAKVSLLPNQLHSDTHISSFRLKVPSPELERSLKIKENLETSLTLKKWLLMLVLLGTSMVIADGIVTPA

MSVMSAVGGLKVGITGVQQDHVVMISVAFLVILFSLQRFGTSKVGLAIGPALFVWFCCLGTTGVYNLVKHDRSVLRAFNP

LHIYYFFKRNSTRAWMSLGGCFLCATGSEAMFADLCYFSVRSVQITFVFLVLPCLLLGYLGQAAFLMENQTKAEQVFFAS

VPDGCFWPVFFIANVAALIACRAMTTATFSCIKQSTTLGCFPRLKIIHTSRKFMGQIYIPVINWFLLVLCLVIVTTFTSI

NEIGNAYGIAELGVMMVTTVLVTIVMLLIWQINIIIVLTFLVFFLGIELTFFSSVLWSVGDGSWVILVYVAVFFMIMYIW

NYGSKLKYETEVRQKLSMDLMLQLGCNLGTIRAPGIGLLYNELVRGVPAIFGHFLTSLPAIHSMVIFVCIKYVPIPVVPQ

NERFLFRRVCPKSFHIFRCIARYGYKDMRKENHQTFEQLLIDSLEKFIRREAQELSLESDEERETDSSDEDEEYSRVLVG

PNGSVYSLGVPLMSNYRSSLGSTTTQASTSGTHFEDHAASDVDPMDQGLESELSFIRKAKESGVVYLMGHGDIRARKDSW

FIKKLAINYFYAFLRKNCRAGIATLSVPHTNLMQVGMTYMV*

>MapolHAK1

MESSSPPRGTTSASEDDQPEGEGTADAVSSQFQKAGPSGRDVLKLAYQSLGVIYGDMGTSPLYVFSNTFEIPPHSTNDIV

GAVSLIFWTLTVIGLIKYVMIVLSADDNGEGGTAALYTLLCRHVNISLLISQHKSDKKLANYKTKKHASHSRLETCMERS

PMLHKLLLVVVMAGSCMVIGDGILTPAISVLSAVEGIQVVATSLHQNVVVLITCLILIGLFVLQRFGTAKVGFLFAPVVL

LWLGLISGIGIYNIVVHQPSIFRAFSPHYAITFLIRNKKNGWIMLGGVVLSITGTEAMFADLGHFSQRSIQIAFAGVVYP

SLVLAYFGQAAYLTKNPENVADTFFKSVPKPIFWPMLVIATLATIVASQAMISGAFSVINQSCNLGCFPRVKVIYTSTTV

QGQIYIPEVNYTLMILCIAVVVGFRKTTAIGNAYGIAVVSVMSITTCLVALVIVVVWHQHFLLGILFLVFFGSIEAVYLS

SVLYKVASGGWVPLVIATFILSIMLIWQFGTLQKHKFDLQHMVHIDQLDELLGDSRITRVPGIGLFYSETDSGIPPTFSH

FISNIPAIHQVLVFICIKYVPVTSVLPGERFYMKPLAVKNFYQCVVRYGYNETPCAGEAFDDLLVGKIAELIKQDKYWQT

AGVSNSSSRGTMSPPVEAKITSSTSEIVHLAKFAAGPPSTRPPRTYIQLSPSSPHSITVKTQAEVINEELKYLYNAKESG

VVYVIGCTEVQAKPGSSWSKRFIIDILYCVLRKNSRSSVIMLNIPTTRLIEVGMIYDI*

>MapolHAK2

MAEEGVAEAGDGPITAESPRISISSARKSSVRKGSSRSGLRRVDSLLVEAGRVKSMGAVHHGTEDLPWSVTLTLAYQTIG

VVYGDLGTSPLYVFASTFPDHTPSENDILGALSFIIYTFTLIPLIKYVFIVLRANDHGNGGTFALYSLIARYAKISVSPN

QVPEDQEVSSYKLAAPTKTMRRSQYLKEALNKKNWLRNVVVTIAILGTCMVIGDGVLTPSISVLSAIQGIKVNKPSLNQN

VVVLVTCVILFALFSIQRFGTDRVGYCFSPIVVIWFISIALIGIYNIIMKDSTVFRAFNPYHMYEYISRNKKQGWISFGG

IVLCVTGTEAMFADLGHFSVKAIQIAFTALVYPCLLIAYIGQAAYLLKHPEAVGETFYNSIPDKVYWPMFVVATGAAIIA

SQAMISATFSIVDQAMTLGCFPRVKVVHTSKKYAGQIYIPELNWLMMVLCIIIAAGFRDTTQIGNAYGVAVVSVFFVTTN

FVTLIAIMIWQVPLWIALPCYFFMGSVELTYFSSILYKVPKGGWVPLIFVACFFSVMYTWHYGRVKKYEYEVKNKVSLDW

VLGLGSNLGITRVPGIGLVYSELAQGVPTIFSHLISNLPAMHQVLVFVCIKNLPVPYVPDEERFLIRRIGPKQFRMFRCA

VRFGYRDLSVGNEIDFEEQLIQNLAIFISTEVQAAQEQPMIADLAQLQRMIDAEGDGGAAPASVALDMTRRLSQGSRDDS

EDSQSQSGIKAMKLPPNVDSGVEEELKVLVESKDAGVVYLLGHTEVRSRRSSSRFRKFLIDDYYHALKRNCRASTVSLAI

PHERLLQVGMVHYI*

>MapolHAK3

MNMSMEAAMKRDEEMGMGTEDRGKMWAIDQRMDQPLGPESGRIRIMNDGKKAVPLAALLRLAFQSLGIVYGDLGTSPLYV

FRSTFPSGLREEHEHEVLGALSLIIFTLTLIPLIKYMFIVLRADDNGEGGTFALYSLLCRYCNISSLPSRHPTDEELTTY

IKTLPLPNSRAESMKTRLEKSSFLQFLLLMLVLLSTCMVIGDGILTPAISVLSAVDGIKVASSKLNQTVVVLISLAILVL

LFSMQRFGTGQIGFLFAPIVMIWFLANGSIGIYNIYQHHHSVFQAFSPHYGIRYLIKNKKEAWSSLGGIVLCITGTEALY

ADLGHFSAQSIQIAFSVLVFPCLLLSYLGQAAYLMHHPEDVKDTFYKSLPHPLYWPMFVLATISASIASQAIISATFSIV

KQSMALGCFPRVKIVHTSHKLMGQVYIPEINWIVMILCLIITAGFKDTEQIGNAYGIAVVGVMLSTTVLMFLVMIMVWHK

NLWVAALFLGVFGTVEAVYYSAVLFKVQQGGWVPLAIAATMLIVMYVWHYGTIKRYEFEIQNKVSVGWVLGLGTGLGLVR

VPGIGLVYTDLAHGVPPLFSHFITNLPAIHSTLVFVCIKYLPVNTVPNEERFHIKRIGPTSFSMFRCAARYGYQDLHKKD

DKFEETLIEKLTHYIKYEALVASTQTNYPGDWTSVSSLTTSSARTTTMESDHSQLPGRTVHFQEEDSPPDSPDGISAPRQ

RGGRPLHPAVIESDDDIGEESVSELETFLKNKEIGVVYLLGNTMVRARTGSGLLKRIAINYIYAFLRRTCRESKVMYNVP

HESCLQVGMVYYV*

>MapolHAK4

MPGMADDGVVGVHDIISSTANTPRVSFAGDNTSVGKSSIRKASSRSGLRRIDSLEAEASRVKSMETHHGNQNLPLSVTLT

LAYQTIGVVYGDLGTSPLYVFASTFPDRVPSEKDILGALSLILYTFTLIPLIKYVFIVLRANDHGNGGTFALYSLVSRFA

RISVSPNQVPEDQEVSSYKLSTPTDSMKRSQKLKEALQNHRWLRTLIVTVAILGTCMVIGDGVLTPSISVLSAIQGIKVN

QPSLDQNVIVVITCVILFALFNIQRFGTDKVGYLFSPVVLVWFISIALIGIYNIIKQDPSVFRALNPYHMYEYMSRNKKQ

GWISLGGIVLCVTGTEAMFADLGHFSVKSIQIAFTTLVYPCLVAAYTGQAAYLLKHPEDVSETFYNSIPEPVYWPMFVVA

TGAAIIASQAMISATFSIVDQAMTLSCFPRVKVVHTSKKYAGQIYIPELNWLMMVLCIIIAAGFRDTTQIGNAYGVAVVS

VFFVTTNFVTLIAIMIWQVPLWIALPCYALEGSVELTYFSSILYKVPKGGWVPLLFVAVFFTVMYTWHYGRVKKYEYEVK

NKVSLDWVLGLGSNLGITRVPGIGLVYSELAQGVPTIFSHLISNLPAMHQILVFVCIKNLPVPNVPDEERFLIRRVGPKN

FRIFRCAVRFGYRDLHGRNDVEFEEQLIHALSNFIKTEVPAGSDAPPLHHQVIDADWAIEGDVPASAIAEAARTLSRGSL

DISEESISQTGIRPMDFRRAVSVEPPVEDELDVLDASKHAGVVYLLGHTDIRSHKKSSKFRKFLIDHYYHALKRNCRAST

ISLNIPHERLLQVGMIHYI*

>MapolHAK5

MDLERGAYGAGQKFQDLQKTSYFAVLGLAYQSFGVVYGDLSTSPLYVYKSAFSGALRKYEEEAVIYGVLSFIFWTLTLVP

LIKYVLIVLSADDNGEGGTFALYSLLCRHAKLSLLPNQQSVDEELSNYGPEQSFVHRPRAAAMQALLEKNKVLRTGLLII

VLLGTCMVIGDGVLTPSISVLSAVNGLQIAAPGIHDNWMVFLACIILVCLFYLQHYGTHKVAFMFAPIVITWLLCISGIG

VYNVIHHQPGVFRALSPYYMWNFFKVCGKEGWVALGGVALCITGTEAMFADLGHFSQLSIKIAFTGVVYPCLILSYMGQA

AYLSKHPEHIGRTFYMSIPKPVYWPVLVVATLAAVVGSQAVISATFSIIKQCQSLGCFPRVKVVHTSNHIHGQIYIPEMN

WLLLILSLAVTVGFKDTTQIGNAYGIAVVSVMLVTTFLMSLVIVIVWRKHILVAAAFFLVFGSIELLYISSSFMKVPQGG

WVPLVLSAVFMLVMYCWHYGTTKKYEFDIQNKVSMKWILTLGPSLGIVRVPGMGLIYTELVTGVPAIFTHFVTNLPAFHK

VLVFVCIKSVPVPHVEVEQRYLVGRIGPKEYRMYRCIVRYGYKDVHEEDHNFEKNLVDSIAQFIQFEGSGDVWFPSSSEN

SAAEAEDTHRTFTSFAAHHGIKVLASDCSDTHSSGSGTTTVRSFQALQDQVDAAARGRKKVLLALQDSADEEGTESIGRK

RSFSNLEEPDDQEETDSLGRKKSFQSSQSTVDGEGTDALGRKKKVVRFDVNPEVDPVVGEELAELYSAREAGVAYVLGHS

YVKAKNASSWFKRFAIDYAYNFLRTNCRGPGIDLSIPHLCLIEVGMIYYV*

>MapolHAK6

MGKTDDGIVEGESSSGSRPGAISKITDAARLSTGGSQKVKSPLALSVHNKSMRHGGSSRGSRRLGLRRVDSLEEEANRVK

AMDSQTHGNQDLPWAVTLSLAYQTIGVVYGDLGTSPLYVFASAFPDKTPSRDDILGALSIVIYTFTLIPLMKYVFIVLRA

NDQGNGGTFALYSLIARFTKIMVSPNQAPEDSEVSSYKLHTASNTLKRSQFLKEAFRKHLWMRNTILTIAILGTSMVIGD

GVLTPSISVLSAIQGIKVQKPDLSQDIVVIITIIILIGLFSIQRYGTDTVGYIFSPVVTVWLISIALIGIYNIIEKDHTV

YKAFNPYHMYDYLARNKKEGWISLGGLVLCVTGTEAMFADLGHFSVAAIQIAFTTLVYPCLLVAYIGQAAYLLKHPEAVG

ETFYNSIPEKVFWPMFVVATFAAIIASQAMISATFSIVTQAMTLGCFPRVKVVHTSKKYVGQIYIPDLNWLMMVLCIIIA

AGFRDTTQIGNAYGVAVVSVFFVTTNLTTLIAIMIWQVPLYFALPAYVTIGSVELLYFSSIMYKVPKGGWVPLVFVAIFF

SIMYTWNYGRRMKYDYEVKNKVSLDWVLGLGSNLGVQRVPGIGLVYTELAQGVPTIFPHLISNLPAMHSILVLVCVKNLP

VPNVLDEERFLIRRIGPKQFRMFRCAVRFGYRDVQERKTYHFEQQLIQNLANFIMTEVTNPPDLTVALGQDVPDLQKGLL

AAEELEGTIRASNIMRSARRISRDVDDEDEDSSSESESLALKAPKKIDAGIEEEIEFIDSSRHAGVVYLLGQTEIRARKS

SSKFRRFLINHYYSALKRNCRKSTLALNIPHERLLQVGMIHYI*

>MapolHAK7

MMPDEDKDKSEDVVLESILATQSRAGGVTPTPDEYSTPNVSGEPSEPVLSEAELSGQPSGNVVIESTTAAQTGAASVTAT

TDDNSAPTGQPDETSISGAKLSGLPSCNVMLDSTIATQITARSVTASTNENPIPIVSGEPSETSVSEAETSGQCSRNAEL

SSPISESTRRKSRRSISSSPDETSLAQTNLHHHRVAAVGFKFVSEEILLANERNASEACCGERHSAGAESDIESRAVSRF

RSLSSRFFKKAFPESPKANTFRETLFLAFRTLGVVYGDLGTSPLYVYPTINVTNPTESDFLGILSLIFWTLTLIGILKYT

LIVLRADDHGEGGTFAVYSLLCQHANIGQDAGDDNQEMSGPRRSQLSEHAASTKQNRTKRFLEGSVAAQRVLLFVVMMGT

CMLVGDGILTPAISVLSAIEGIQTAAPSLNRAVVVVVSAVILVVLFACQRFGTHRVSSVFSPIMALWLITTPIVAAYNIA

IYYPGIFKAFSPAHIYYFFKQNKKNAWLMLGGTILCITGAEAMYADMGHFDKRSIQLAFTTMVYPSVLVTYAGQTAYLIK

HPEDHREGFFKMVPKKVYWPVFVVATLAAIVASQGLISATFSIIKQSTALHYFPRVKLVHTSENKEGQIYSPEVNYVLMV

LCLAVVFGFRKSGDIGNAFGVAVVAVMLITTCLISLVMLVIWQTPVFLVLPFFLLFIVVEGTYMSAVVTKVPQGGWLPFA

VSLIVTLIMFSWNMGCQTKVHYEMRNKITKEKLGELLAHGGSARVPGICFFYTNLLHGVPPIIGHYVRNVRSLHRVLVFT

TIRWMPVRRVLPAERFLVGRVGFSGVYRCVARYGYRDVLQMQNDEFVDQFVATLSEYIMATEQTNHAALPQSLQDNTLSP

LAIASEIADLNQAKADGPVYVLGRSELTVSRKTRMATRIFVGVFYRFLEANCRSEVSTMDIPYAQYLELGMLYDII*

>SphfalHAK1

MEPSASSTTTESELKPVGDGRRDNHVSINTVELSNHTKSLNSTTSKTNGSAKNLTGRFGFPRVDSLDVESSKMPKDFLHG

KNKDKTSIMLALAFQSLGVIYGDVGTSPLYVYSETYVLTHNPAGPSKEDLVGVLSLIIWTLTLIPLIKYVFIVLRAGDRG

NGGTFALYSLVCRHARINTVSNQSPEDRELSTYKLDLPTRSAKRAANLKGIMERSKLLQQILLLMALVGTCAVIGDGVFT

PAISVLSAISGIQVPAPNLPQGVVTGISCGILFVLFMVQRFGTNKVGYTFAPIVLVWLLSIGLTGLYNIFKHDPSVLKAF

NPAYCFTFFTRNKRAGWISLGGIVLCITGTEAMFADLCHFSVRSIQLGFTTFVYPFLLMAYIGQAAYLSKHPEHISQTFY

KSIPSPLFWPMFVVATAAAIIASQAMISATYSIIDQSIALSCFPRLKVIHTSEKYHGQIYVPEINYILMVITIVICIAFK

GSTSLIGNAYGIAVVSVMVVTTTFLTLIMILVWQVNLWIALAFCVFFGSIELVYFSSCLFKVPQGGYTPLIIGAVALFIM

CTWHYARKRGYDFEVKNKISLDWVLALGPNLGGVRVPGVGLIYTELAQGIPAIFSHLISNLPSMHSVLVFVCVKNLPVPN

IPDDERFLLRRVGPQNFHMFRCIVRFGYKDYQEDIKDFEDLLFADLSKFIRTEHIESWQPASDGGLQDDITPDGATAVTV

EPFSEDSTNHGLVFGSQSSQEEYDSKQITPLSTKPSLESINEGLEVDELRYLHKVRRIKVAYILGHIDVRAQKDSNVVRK

VVIDDIYGFLRRNSRSNVMALNIPQESLLRVGMINYI

>SphfalHAK2

MESSSSVELQSKGGVEKKKKLGEAAAAAPGLLSSSVQAAGGGAGAGDGKLDADAEEEEFGFDANGEIASDAVVVGNMNES

TTTTTTKGTPHGTPPFSRRLSRKLTRPDSLDVESMRVRGMEHGGHPALSLGIILQLAYQSIGVVYGDLGTSPLYVYSSTF

QGNMVTSDGLIKTPKDILGALCLIIYTLTLIPLIKYVFIVLQANDNGEGGTFALYSLICRHARIPLAHRQVPADRELSTY

KLEKPTRRNARAARIKGILEKSTFLQNALIITVLCGTCLVIGDGSLTPAISVLSAMQGIEVEVPAVKPMVVVILTIVVLL

IMFSLQQFGTNKVGFMFAPVVLTWFISIGLIGVYNIVSNDPSVFRALNPLYIISYFHRNKVEAWKSLGGIVLCITGTEAM

FADLGHFRVSSIQIAFSGIVFPSLLSAYIGQAAYLLKHNTAEDVSYTFYKSVPKPIFWPMFAVSTGAAIIASQAMISATF

SMIRNAMALGCFPRVTVRHTSKRIHGQIYIPEINWLVMCLSIAIVAIFRSTTQIGNAYGIAVVGVFFISSSLLTLIMIMI

WEKNIFLALSFWCIWGGVEGMYFSSVLSKVPQGGWVPLAIASFFLTVMFSWHYGNRRKSMFEVQNKLSLDWLLGLGSSLG

VARVPGIGLVYTELPVGVPAIFGHFITNLPAIHSIVVFVCVRNLPVSSVPSDERILIRRVGPREFRMYRCAVRYGYTQGT

GDEDEENFEDLLAHSLASFLRTEVMENTPASITSTVSTHHNNIAHHAHFEMAEGDTDATANHSMSSEQKLDGDRLPGSVL

PYTKESSEVASQVDNSYSEDEVTYLYKAKHAGVVYMLGESDVHCSKKASVLKRFIVNGVYSFLRRNCRPNTLYLSIPRMR

LLKVGMTYEI

>SphfalHAK3

MDLESGSSRSSSQRPPQEDVSLRVILLLAYQSFGVVYGDLSTSPLYVYRSTFSGRIKLQQDDAEILGVLSFIFYTLTIVP

VIKYVFIVLSADDNGEGGTFALYSLLCRHAKLSLLPNQQAADEDLSTYKLKPPPETTQGIWLKKLLEKHEKLQTALLIIV

LLGTCMIIGDGVLTPAISVLSAVSGIQVAAPHLHDNVVVAVACVILVGLFSLQHYGTNRVAFMFAPIVIAWLFCISTIGV

YNIIYYDPTIFRALSPFYMYNFFKKCGIDGWISLGGILLCITGTEAMFADLGHFSQLSIKIAFTGVVYPCLLLAYCGQAA

FLSKNRDDISQSFYASIPKPVFWPVFVVATLASIVGSQAVISATFSSVKQCLALGCFPRVKVVHTSQRIHGQIYIPEINW

ILLILCLAITVGFQDTTTIGNAYGLAVITVMLVTTCLTTLVIIIVWRRSIFLALCFLLFFGSIETLYISASIIKVPQGGW

APLALSIVFMSIMYVWHYGTTKKYEFDLQNRVSMKWLLTLGPSLGIVRVPGVGLIYTELVTGIPAIFSHFVTNLPAFHKV

LVFVCIKSVSVPYIPQHERYLIGRVGPKEYRMYRCVIRYGYKDLHRDDHDFENELIINLGEFIQSEGDVPWIPSSSEVSA

DGQVGVMSSANSARIMTTLTSDSDPEESYISMPSHNDLSQSSTSIQVAEISESLEIDQKKRVRFEIPKSPNLSPAVREEL

MELIEAKEAGVAYVLGHSYVKAKRSSSLLKKFAIDIAYTFLRRNCRGPTVALSVPHLCLIEVGMIYYV

>SphfalHAK4

MAGSNGDFEDRGGQGKMRNLDRTLDQPLGLEAERVSSMRNQKVLPAAVILHLAFQSLGIVYGDLGASPLYVFRSTFPQGI

TNKQDVIGALSLIIYTVTFIPLIKYMFIVLKANDNGEGGTFALYSLLCRHCNISSLPNRHPTDEELSTFVVDRQHPKTYL

QKKLEGSHVLQRLLLGLVLVGTSMVIGDGILTPAISVLSAVSGIKEASSSLNDNVVTIVSLVIIVSLFSMQRFGTARVGF

FFAPVFLLWFVCIALIGIYNIAQNDKGIFQAFSPLQIVYFFKRNKRAGWEHLGGVVLCITGTEALFADLGHFTSRSIQIA

FSTVVYPSLLLAYLGQAAFLLNYPEHSDNPFYKSLPVKLYWPMFVLATLAAIIASQAIISATFSIVKQSVALGCFPRVKI

VHTSDQFQGQIYIPEINWLLMILCLVITAGFRDTRQIGNAYGIAVVAVMLVTTYLMTLVMLMVWHKPLIVALIFLLLFGS

VESVYVSSVLFKVKQGGWVPLVVAVAFGSIMYVWNYGTLKRYEYEIQNKVSVGWLLGLGPSLGLVRVPGIGLVYTDLAHG

VTPLFSHFITHLPAIHSTVVFVCIKYLPVSTVPQEERFLTRRIGTKAYSMYRCAARYGYRDLHKKDDDFEYLLFQSLIRF

NEFEALQNSFGADSLAASWTPEERSVSSLPALEMAPVIGNSMLQGDGLENNSAYADSSIQPPSMSEVFSQEISAATEHQG

AHFQTSPITTCEQDIHNELRSLTRAKDAGVTYLIGDTVVKARRDAHYLKKLAINHAYTFLRQTCRESTVALNIPHESLLQ

VGMIYYV

>SphfalHAK5

MAAGNGDLEVEGHGHGEGKMWNMDQTLDQPLGLEAERVSSMRVQKVLPAVMILRLAFQSLGVVYGDLGTSPLYVFRSTFP

QGITNQKDVVGALSLIIYTISLIPLLKYMFIVLRANDNGEGGTFALYSLLCRHCSISSLPNRHPTDEELSTFVVNRHHPK

TYMQRKLEGSPVLQRLLLVLVLIGTSMVIGDGILTPAISVLSAVSGIKEASSSLNDNVVTIISLVIIIGLFSMQRFGTAR

VGFVFAPIFLLWFISIALIGIYNIIQHDKGIFQAFSPLEIVYFFKRNKRAGWEHLGGVVLCITGTEALFADLGHFTYGSI

QIAFSGLVYPSLLLAYLGQGAVLLNHPENADDPFYKSIPVKLYWPMFALATIAAIIASQAIISATFSIVKQSVALGCFPR

VKIVHTSDQFQGQIYIPEINWILMILCLVITAGFRDTRQIGNAYGIAVVAVMLVTTYLMTLVIIMVWHKPLVVGLIFLLV

FGSVESVYVSSVLFKVKQGGWVPLVIGGALGSIMYVWNYGTLKRYQYENQNKVSVGWVLGLGPSLGLVRVPGVGLVYTDL

AHGVPPLFSHFITHLPAIHSTVVFVCIKYLPVNTVPQEERFLVRRIGTKAYSMYRCAARYGYRDLHKKDEHFEQLLFQSL

IRFNKFEALRNSTGDNSLAASWTPEERSVSSVQALEMAPQNPIMDDSMSQGDGLEINSIADSSIQLQSVSEDPFQEIPAT

ERRGVHFPTSPIEEDTQDALTCLMRGRDAGVTYIIGDTIVKARRDAGFLKKLVINHVYTFLRKTCRENTVALNIPHESLL

QAATSVMGCRNDSQVTSHQIPGGRFSSHV

>SphfalHAK6

MAAGNGTPAAIVAAAMMEEGLGMGTVNGHDKGNFWSLDQNMDKPLGLEANRVQSMQYRKVLPLGIILRLAYQSLGVVYGD

VGTSPLYVFQSTFPDGVTDGRDILGALSLIIYTITIITLVKYMFIVLRANNDGEGGTFALYSLLCRHCNISALPNKHPTD

EELTTFVVNCGSIKKTWLQKKLESSKVLQRILFIVVLCGTSMVIGDGILTPAISDVVTLISLVILVLLFSVQRFGTARVG

VMFAPVFLLWFISIAVIGVYNIIKYDKAIFKAFSPLEIIHFFRRNGRAGWIHLGGIVLSITGTEAIFADLGHFTFQSIQI

AFTGLVYPCLLLAYIGQAAFVSRNLESVGDAFYSSIPHRLYWPMFALSTVAAVIGSQAIISATFSIVKQSVALGWFPRVK

IVHTSNQVPGRIYIPEINWMLMVLCLVVTAGFTNTSQIGNAYGIAVVAVMLVTTVLLTVVMIIVWHKPIFVALAFLMVFG

TIESIYVSSALYKVSRGGWVPLVIAVAVGSITYVWHYGTLKRFEHEIQNKVPMGWLLGLGPSLGLVRVPGIGLVYTDLVH

GVPPIFSHFITNLPAIHSTVVFVNLKYLPVSTVPQEERFLIHRIGPKVYSMYRCAARYGYRDVHKKDDRFEQLLIESLIT

FIKFEALQGSSAHESLAASYTPDEESVSSMHQPTGAMNSPAMPGVLNLQGDKLDTSTLEGSSTIYNPALLNPSQEISSIH

SRDVQIPNSQGVDTTSRDETAFILKGKDAGVVYLLGNSVVKARTDSSFVKKLIIDYAYSFLQRICRDSHVTLNIPHESLL

QVGMVYNV

>SphfalHAK7

MSNKLILQLAFQSIGVVYGDLGTSPLYVFSSTFYHGSISNTDDVLGALSLIIYTMILIPLVKYVFIVLQANDNGEGGTFA

LYSLICRHARVSPSHRQQHEWPAAHDSAPTTLTIGSDTRRGIRAAYIKEQIENSSFWQNVLLLTVLIGTCLVIGDGCLTP

AISVLSAIEGIIVEAARIPQYVAILLTVVVLMAIFSVQKFGTGKVGFMFAPVVSIWFLSIAAIGIYNIFKYNATIFRALN

PLYIVAYFQRNKKDAWISLGGIVLCITGTEAMFADLGHFTMKSIQIAFTALVFPSLLAAYMGQAAFLMKNHTDLDAQYTF

YRSIPRAVYWPMFVVATAAAVVASQAMISATFSMIRSAMALGCFPRVTVIHTSRKYHGQIYIPEINWLLMVLSICIVAGF

RSTNDIGNAYGIAVVGVFFISTCLLTLIMVMIWQKHILICLGFFVIWGTVEGVYFSSVLSKVDQGGWVPLAIASSFLLVM

YAWHYGTELKYLFEVQHTIPMDWILGLGSSLGMARVPGIGLVYTQLPQGVPAIFAHFITNLPAIHSILVFVCVRNLPVSS

VAPEDRILIRRVGPRQYRMYRCAVRYGYIDDVGHGCDENFGDLLVESLARFIRTEAGVWSSPAPIGSHSPRLGRRQQQQQ

QDPTTTTTTTPSSPGAKGQQSFSSPAQETIGESTDIIGEEFPVPSSTGVVETGDPLHIAEESEGNPRVVKLYECTRPGTC

SSNVREVDEMGYGARGISSRTLGDDDDSLNAEQELAYLYEARAAAGIVYLLGYSEVHCKQDTSFLKKLLINVYSYLRRNC

RTSELYLRIPHSRLLKVGMTYYV

>SphfalHAK8

MESVVQGSFQAPNGVEHQFDMEEGRVEMAPAGATITSVDEHVTPTPETNFGALFKEEMKPCRKKPKEYSKWGIAVLAYQT

LGVVYGDLGTSPLYVYPTIQIDSPQEEDFLGILSLIFWTLTMIGLVKYVFIVLQANDHGDGGTFAIYSLLCQHANIGQNA

GEKLQKHESDVLLSFHGRSRQSRTKRFLESSVFWQKVLLFVVLLGTSMVIGDGVLTPAISVLSAVVGIRSAQPSFDQSKV

VWVSLVILVGLFLLQRFGTSKVAFIFSPIMVAWFLTTPTIGVYNIVKYHPTVLKAVSPHYIVIFFKRNKVNGWKMLEGAV

LAITGAEAMFCDLSHFNKSSIQLAFSSFVYPSLILTYAGQTAYLIKNPGDISDAFYKSIPHAVYWPMFVVATLAAIVASQ

ALISATFTIVKQSMALGCFPRVQIRHTSKDEEGQVYSPEINYLLMILCIAVVAGFKDGTQIGNAFGVAVIWVMLITTVLM

TLVELIIWRLPWPIVLVFFTVFGTIEGVYMTAVLNKVPQGGWVPFAIAAVFLVIMSSWNYGHQRKIHYEMNNKMSVHSLQ

DLILNSEVQRVKGICFFYSDLVHGVPPIIPHYIRNVQTLHQILIFSTIRYLPIRTVLPEERFLVGRVGIKGAYRCVARYG

YMDVINLKGNEFKDQVLQQLQAYLQSEDQNNLSFVQRSSVRLVNESGRVNHNGVPNQPQLNLEDIRDLNLAAQHESVHVL

GRTMLECGPSTGYLDRIVVNKIYRFLKMICRPAVALRIPSANFLEVGIYYEL

>SphfalHAK9

MATTNGKDLEVGTLHEHDQNSCDDPNFEHQIDKEYGDSMDHPVGNGVMNTNGFGGPERLDVKEAANGKIEVHHKHCQLQR

VSDMQFSTQMVPLHADRQKSTTWGTLFLAYQTLGIVFGGLGTSPLYVWPSINLSDPKEEDFLGVMSLIFWTFTLIALVKY

VFIVIHADDHGEGGTFALYSLLRQHANLGQKSKALSDSVKLKEQISQSSGPVHKKTLALLENFKGAQVTLFVVVMLGTCL

VIGDGILTPAISVLSAMAGIQSEANSISGSVVTWVSAVILVLVFMMQRFGTNRVSFLFSPIMLVWFVATPIVGIYNIVVH

YPSVFKAVSPHYIVDYFVRNQKQGWVALGGVVLCITGAEAMFADLGHFNKRAIQIGFTAVLYPSCLFTYAGQTAYLIRHP

MDHRNAFFKSIPQAVYWPLFILATLAAIVASQSLITATFSIMKQTIALGCFPRVKMVHTSADQEGQVYSPEVNYVLMVLC

VAVVLGFRNAARVGNAFGVAVLGVMFISTLLMTLVMLVIWNLPWPLVLLFLTLFGSIEGVYFTAVLNKLPEGGWVPFGFA

AFFLFISLTWSYGRQKKCKYEMNHMISLNNLGALLSSAGMQRVPGICFFYTDLVDGVPPIISHYVKNVRTLHQVLIFTTF

RFIPVKTVSPEERFLVGQMGFKGVYRCVARYGYLDVIDCEGDKFKNQAIQSLQSYLQSEDHMELSTVQISNGTSSDLQYQ

SMENPVDMYDPEDLVELQSATGHDAVHVVGKITVRTSSSTCWLGRIAINKIYAVLRLICRSAIKELQIPPANYLEVGMLY

DV

>SphfalHAK10

MATNGKDIELGILHEEGQDSCDDGREYECQIDKEYGNGINHPASSCMVSNMTVQQVGDHERLDVGEATSGKVETSNKQHR

LRRAVSDIQFKAHMAPVHPDRKKYTCWGTIGQAYQALGIVFGGLGTSPLYVYPSVNLPNPQEEDFLGLMSLIFWTLTLIA

FVKYVLIVIRADDRGEGGTFALYSLLHRHASVDQKDTAASDSETTHSSGQVHDKVHSLLKNSRTAQIILFIVVMLGTCLV

IGDGILTPAISVLSAMAGIQSEDESLPPAVVTWVSAIILVVVFMLQQFGTHRVSWLFSPILLVWFVTTPLVGIYNIAAHY

PSVFKAVSPHYIIKYFIRNKKQGWVSLGGAVLCITGAEAMFADLGHFNRRAIQVGFIAVLYPSCLLTYAGQTAYLIRHPM

DHQNAFFKSIPQPVYWPMFIVATLSAIVASQSLITATFSITKQTIALGCFPRVKMVHTSADQEGQVYSPEVNYVLMILCV

AVVLGFQNSTTVGNAFGVAVLGVMFISTLLVTVVMLMVWKLPWPLTLLFLSVFGSIEGVYFTAVLNKVPQGGWVPFAFAA

FFLCISLTWSYGRQKKQTYEKNHKISLDSLGALLSSAGMQRVPGICFFYTDLVHGVPPIISHYVKNVRTLHQVLVFTTFR

FIPVRTVLPEERFLVGRVGFKGVYRCVARYGYQDIIDCEGDEFKNQAIQSLRSYLQSEDRMELSTHQISNGTTDLQSPSL

KNLAEMYDAEDLAELESATSHTAVYVVGKITVRTSSHTGWLGHLVIDKVYTLLRLISRSTIKELKIPPANYLEVGMLYNV

>SphfalHAK12

MAKPLGDVEAKSQGDGEGKLWNMDRPLGFEAERISSMRYQKVLPVAVIFSLAFQSLGVVYGDLGTSPLYVFGSTFPHSVM

NRKDLIGALSLVIYTITLIPLVKYIFIVLRANDNGEGGTFALYSLLYEKLSTFVVNHHHHRSYLQKKLEGSQLLQRLLLI

LVLFGTCIVIGDGILTPAISVLSGVSGIKRASSSLNDSGWDMTEFLNLLADMVTIISLVILVGLFSMQQFGTARVGFMFA

PIFLLWFVSIGLIGIYNIINHDKSIFHAFSPLHIVCFWTEVLFTDLGHFTYLSIQSCLVHIAFSGVVYPCLLLAYLGQAA

YLLNHPENAGNPFYKSIPGKLFPLDHSEQYLPLLQAIISATFSIVKQSAALGCFPHVKIVHTSDQFQGQIYIPEINWALM

TLCLVITAGFSDTNQIGNAYGIVVVAVMLVTTYLMTFMVWPQASDGGWVMLVIAGALGSIMYVWNYGTLKRYEYEIQNKV

SVGWLLGLGPSLGSVRVPGIGLVYADLAHGIPPLFSHFITYLPAFHSTVVLICIKYLPVNMVPQEEHLLIGTNAYSMYRC

AARYGYKDLHKKDDNFEHLLFQSLIRFIQFEALQNPSDSMQTLIVRDSRLQDNGLESISTDSSIEVTQEILCIRRPGVHV

SSSPSSNLETQDELTFLMEGRDAGVTYLIENSVVKARRDASWIKKLAIDYVYSFLCRTCRERSVALHIPHESLLQVGMFY

FVWVFLNKWR

>CosubHAK2

MKRSLTDLIRKKEPGRQGFADSILSRVTSKAWENDADIEDPGAAERSGKAKWRGTLILGFQALGVVYGDIGTSPLYVISS

TFLDGAPSEEDIVGVISLIIWSLTALLVIKYAAIVLRADDNGQGGTFALYSLLKRQAELGNSGKLMESDRHLSQYSIGRG

DTRLASRLSRRKRTQSAPPGGLPTVTEYSTKLVDWRQRFIENRHTQNILRVLVVAGVGMIMGDGVLTPAISVVSACEGLQ

QASANITRSMIVIIAIVILAGLFMIQQFGTKFVGYLFSPIILVWFLFNSVVGIYNIAKYRPVIFKAFGPNYWFSFFLRNQ

KGGWQALGGVVLCITGVEALFADLGHFNRPSIQISTFCIVYPALIITYLGQGSYLLAHPDAFDAMFWKSLPQGTFWPMFV

VATLAAIIASQALISAVFQIVSQAIVQGFFPRFHVYHTSREHRGQVYIPLINYLLMALCLIIVGTFQTSTNIGRAYGISV

LADMFLTTHFMTLVLMTIWRLPLPLVVLWYCVFAPIEATYLSSALEKIPTGGWFSVMMSGIYTCIMLLWFWGNSKKKAFY

GRKKLKLHQFLALMGDDGKDEQTSMTIASQKIALKASATKLKRVRGVGLYYGEDIHGVPPVLLQMVSRTPVLYEVNIFVT

NRFVPIPEVLPSERILVEQLGVSGFYHIVARYGYMEEVKQDDAFVRVLLERVLHLLLITLQERASAMPTLHHDLGLPASH

KVPPPSDTNFKARDDSAKHDTPLTAGGAAAEVQLTPVTTTAAQPAAAAADGAPAPTLVEIPGQGRSLSPSDHVPEPFLYR

NLEEVANKLATAPKEVATRYHRAAIVADEIRVVKHAANQHNVVFILGHTHVVLPKRIPFWSVPRRILLELPFKMFADAFS

EPADSIFNIPSAHLLEIGLPYTLDA

>kflHAK1

MPSFVAPENIAIPPVDETETREQNGATAPVTPRSPQWAAEEVLVEGKMHKKHKALSTSAVLLLAWQSLGVVYGDLGTSCL

YVYSNTFHSPPSHQDLLGAACIIFWTLTSIALVKYVMIVLHADDDGEGGTFAMYSLLCRHARISLLETAKLNVTAKPRLT

RAQSSKVSARGVVRGLLEGNKSLQKALMVFVLMGTCMVIGDGILTPAISVLSAVEGIHVGYSGLSRSAIVGITCAILIAL

FLCQHKGTGRVGNWFAPVIGIWLVANLCINLYNISKHHPGMFRCINPWHGLDYFIRNGKTAWVSLGGLVLCITGTEAMFA

DLGHFPRQAIQMGFLLIGYPSLVITYIGQAAYLWQHPENYASTFYSSVPGPIFWPMFVLATLATIVASQAMISGAFSIIK

QSMSLSCFPKVRIIHTSKTMEGQIYIPEINYVFMVLTIAVVVGFQDSTQIGNAYGVSVMSVMLITTFLMSLVMLMVWRLP

PWLPALFFAVFGFIEAIYLSSTLYKIANGAWFPVLLSGLLLVIMYAWYYGSSRKSLFDQKNMVNAQGLTEFLANMPVTRV

PGVGLFYSDTAHGVPPVFTHFITHLPAVPEVLIFMNIRHEPVPRLEDEDRLLVKPFGIKGYYRCVVRYGYQESVRQGEEF

TTQLLNKIIEVTRFELGDSGPVDASPESPRLEASLSSSPLVSSAPVKLAPGKSFIAGPRERAQVEEDLALLEQARSGGIV

YVLGRSVIHVSPSAWVGKRWTLYLYAGLKAISRSTTQNYQIPHSKLIEVGMIYDV

>kflHAK2

MTPESSESASLPILALRSLLSKSKSCMAMDKAGATIKPASEGSSVNGTRPEVEMGPTASVWTGDADLEGQKKRHLAKKAG

LLATIGLAYRATGVVYGDIGTSPLYVYSSTFSDGTPSTDRILGCASLIFWTLTLAVLIKYMCFVMFADDNGEGGTFALYS

LLCRNANITPGGGAAPVAADLQLSRYTSKHSSRQFLTRKTPARVLKNMLERKAWARNALLILVLVMTSMVLGDGVLTPAQ

SVLGAVYGIQVKLPSTSTSVIVGVSCAIVVALFAAQPFGTGRVGFMFAPVVIIWFASNVVIAIYNTATYYPGIYKCLSPH

YAYFFFKDDAHRGWRQLAGVFLAITGAEATFADLGHFSRAGVQLGFVGLAYPSLVITYFGQAAWLIKHPDEVGSTFYASI

PGGDGLFWYMFVVATLAATVASQAMISGAFSIVKQSMALGCFPRLRVFNTSDRVTGQVYIPEVNWVMMILTVIVIVIFKN

TTKLGLAYGVAVSAMMVGTDLLIFIVMLMVWESNVVAAVAFLLFYAFFDGTYLSANLEKVPDGGWYAILVAGVVSALSLI

WYWGTSKKLTYLFANKTRLEDLVTVHRPPPSSDAPSERALAPRDGTLANARTGLPILRAPGIALTYSETLVGAPPLMPSL

IDRWSCLHQHVVLVTVRQVAVPRVDPEERILVQKLPYPGMYRAVARYGYLDRVNHGSAFVLKLTDRLSTLDPDIKRAAAD

ADAAAGAGVGTVLYIFSRQKLTSHKGEDDNVVLRWVRRFMLETVYGTMVKFAHVSHEEWGIPHANKFEGGASAGQWSESR

RFVCALIPTGDSGGPGRLCIRSSR

>kflHAK3

MENRQPKQLVFKGRQETALELAPSNCEADAEPPVLHIAQVASISDEDWLHDHDLEDALVGQKAKASASWGVFFGLTYTSV

GVVYGDLGTSPMYLWQTCFSFPATESDIVGALSLVLWTLTLIVVFKYVVIVLCANDQGEGGTFAVYSLLCRHAKITPFGN

LHDSDKGLSQYSKEGAKERRRLPKKLAAALEGSPLLQNVLLTFVLCGTCALAGDGVLTPPVSVLSAMTGLQVPTVLLNAD

GSTKISNGVVIGATCAVLIGIFLSQQFGTSKIGYVYAPILIVYFVVNASIGVYNISKWEPSIFKAFSPSYGIDYLVRNKF

NGWKSLGGICLAITGVEALFADLGHFNVSSIRASSMFLVYPSLLLIYLGQGAYLSKNPGDVSQAYYKGIPTPVYWPVFVL

AMLASIVASQAIIAGTFSVLAQSARLNCFPRVKVVHTSKVVEGQIYIPEVNWILMVLGLACVLGFSLPQPSDQPVNGEAL

GNAFGIAVMFVMVITTCLVTLVMLLVWNVRPLLAAAFFGFFFVFEMVFLSANVYKVEHGGWFPLAIAVIVFGISYVWHWG

STQRCLHSAKLGSQLEDVIVAEKNEIEEAPQTAPSDSVKVCLKSSGHRIYAARGAALFYCDTLHHLPPIFAFSLEAMPTV

HEITVFLTIRQVPVPHVLQKERLLVKPMAYPGFYWVLARYGYLDQPNHSAAFARQVLKTIAHYLEHGSSCPDLQALDPDG

QDPTWSLSRAASRKLNPRIALAGTQLNGTEQNGMPDLTLTDASKALGLSPPPSEASFENVESKPPLFKPASQKPANTTID

ENGISADEQDLLPDDTGVEISDYARQVKLLKDAQRQRILYLKTRSVVRAAKDSSVLRKFVLESAYNGLLNLSRSPAHILN

IPYSQLLELAMVYEL

>kflHAK4

MARFRGLFERVTRAQWTADADIKVPETHSTKGAGLLWLAFGTIGVVYGDIGTSPLYVYASIFPEGPPSNTEVIGAMSLIF

WTITLLLLVKYALIVLFADDNGNGGTFALYSLLRRQAERGVMGPALPSELELSHYGSGSFAKAKAQSMQRGWRQRVVTNE

TLQKIVRVLVVIGVGAILGDGVLTPAISVVSACEGIQIADPSFTRGAIVGLAMAILAGLFLIQQFGTALMSKVFSPVVML

WLLGNAVIGVVNIAKYDASVFKAISPHYFMRYFIDDGKTAWKSLGGVLLCATGVEALFADLGHFNRQSVQLSCFALLYPC

IILTYFGQTAYLLKHPENVGSTYYKSLPHGTFWPMFILATLAAIVASQALISASFQIVYQAIAQGFFPRFHVVHTSRKIL

GQVYIPLVNYMLMTLTLVVVGIFQKSAKLGQAYGLAVITDMVLTTHFMTLVILTVWRRPLYEGLVFYFFFLLVEGSFLSS

TIEKIPKGGWFSIMMALIYAAIMLWWYWGSSHKRAFFHTKLMPKLDRFFRVKVAEDSKDEQLFIAKSDTRVLRSPGLGLY

YSDSIFGTPPVLVQHLKSFPIIHEVVIFMTNRFVPVPEVLPAERFLIEQLGVGGFYHCIARYGYMDRVQQGTDFAETVLQ

HILILLTDSLKEAVSDNGAYRMRSPTDTWGSLDFSHHGNQNSLRSIRETAPVTHPPPPPADVSIMIPPEVERRKATPSST

PSSSAYGDLEMQQLDAVANGSQINDEQDFTSENENTVGSAKDLSLPANPEVRWGAADFSLGRVAGEALRKLDEATKEWLD

EVVDKLADAERVPSRHKRAAVLAKEIRLVRKARDSQHVVYMLGRATPQIAEKPTNIFKRLFLEIPYLILVNNFHSDAAKT

YNIPQDKLYEVGMQYKI

>kflHAK5

MEVQQNPIFEDAPMEEPWSGVEGSQGTVVREDGPQTEAERPLEESTRMDVLEAPAADHEWAPPSSLDPAARLPPNLHNRA

AAISDDDWLHDRDLADALDSHRSKSSSSWLMFAGLAYKSIGIVYGDLGTSPMYLWQTCFTKTATEEDIIGALSLVLWTLS

LMIFKYVVVVLPANDMGEGGTFAIYSLLCRYTKITIFGLKHDSDKALSVFHQDPKRPKKKRSHPGRAVGKALEKSPALQK

MLLVFVLCGTCALVGDGVLTPAVTVLSAMTGLQVPKELLREDGSTIIPNGAVIAATCVIMAIIFGCQRLGTNRISFIFAP

ILISFFVLNAGIGMYNIHTWNPAIFKAFSPHHGIMYLVRNKKEGWISLGGICLCITGVEGLFADLGHFNVQAIRASTLTV

VYPSLLLIYLGQGAYLSRNPGDTPQAYFKAIPEPVFWPAFVVATLATMVASQAIISGVFSIISQSMSLQCFPKMKVIHTS

KTVQGQIYIPNVNWALMTLGIACVLGFSLPQPSDMPLNGDALGHAFGIAVMCVMIITTCLVTLVMLVIWRTHVVVIAIFF

GWFMFMESIYLSSNLFKIAHGGYVPLTIASVVLLISYSWHWGSTKRYLVGCGMGAQLEDVLVPDESAYAPTFSSDDEDIP

SGPKEPLHYRLKSSGQKVIVTKGAALFYCDTIYRLPPHFAFSLKAMPAVHEVMVFMSLREGPSIAPPTSTKGASIVWIGY

NSLNTTTSANLFETYNLLSVDRD

>ColorbHAK1

MGTLHQTITQKYNTSKGNPFSTTLSLAWSSIGIIYGDIGTSNLYVYSTVFAEFRKRSVLLNDRKDPCTAYKPGTCDYHSV

PDASPEDVVGSVSCIFWTLTLLALFKYVFVIMYADDNGEGGTFALYSNLCRHAHISLLGSSQDVSLSKKLSTAYSSQKKQ

KSFLTEFLEKSLSAQRFLLIITLLGTCCVLADGVLTPAISVLSAVSGLTEAPGLEHLGQRTVIAITVTILIVLFASQRFG

TSLVGVFFAPITALWLVCNALIGIYNISKYKHDIFRAVSPHYSIRYFVRNKKDAWTSLGGLVLCVTGTEAMFADLGHFSR

LSIQLAFSSFVYPLLLLSYFGQAAYLHEHVDVIEHAYYKSLPKPLFWPMLVLATLAAIVASQALISGAFSLVQQSMTVSC

LPGMRVLHTSQHHIGQIYIPAVNYFLMVLTVLVVISFKNVEAIGNAYGVAVVSVMLMTSFMMALLITIVWKYPLWLACLF

FGVFGSIECMYLSALAVKVPKGGWFPVAVAIILLGMMYTWQYGTSLKVEFDKKHMMEVQQLEVLSRNDQLGRYSGMGLFY

TEISNGVPPVLSHFLTNLPAIPQVVVFLTVRRMPVPRLMEEDRLAVVTVGLKNFYRVVAYYGYTEDIDHGEEFVDKLCDD

IKAYICKENNIPILQNENSMTHTSATSSILSSSKYQTRRNTVAGRYLTPSTNDDDSTLSDGVQLNFSSPNLNDIGKLPET

ESTCPIGLPSILNLNHATSAVARLGSQQAPSNPNDPNSTIPPPRAMPLHGTKSLALKAWEPKTGNTDRENTIKALRAAMP

PTAVEGEGREG

>ColorbHAK2

MADDHHHNGLWHHGPSPHTPLLQSPVATPVAPDDGLLASMRRDGSVRGGGGGGTRSRPRIHIPASPRILASSIDEEDGPD

DGFEEGGRKYHHHKPEGILATFALAYGSIGVVFGDLGTSCLYTVSSIFNSFPKEPNREDILGAISLIFWTITLIAVVKYV

FIVLFADDNGEGGTFALYSLLCRHANASLVLKPLKKKEEKDNDKKDLTRKQSMRSLSRNFLETRKPLRKALLIVVLLATS

MVLGDGVLTPAISVLSAVQGVQVAGPKLSTDQVLTITCAILLALFLFQRFGTARVGFIFAPITILWLIACASIGVWNLYQ

FGFGWGFNGMGILQAISPHYGFNLIFKHGTAGFTCLCGVILCVTGTEALFADIGHYSRSAIQLAFAGFIYPCLLTTYFGQ

GAYLCSHLGDVGDTFWKATPHKVFWPVLVLATMASVVASQALISGAFSLVKQSMSLNCFPRVKVIHTSDSMVGQIYIPEV

NYILMILTIAVVLGFQDSTKLGNAYGLAVASVMILTTSLMSVVMLIVWDKGIFLTLLFFITFGTIEGAFLAACLPQIPNG

AWFPLALAAVFLVVMYAWQFGATKKENYDTTHKVRLSQIMEFISRYEVARVPGIALHYSETVSGAPPALSHSLTTLRSIH

KVLVFLTVKPVPVPWVSDEDRFEIKRTAMPGAYRCLVYYGYMEEINHGSDFIDDLVYNIADFVREEGYPILKKPLQPGEE

PTHERLQAADVLEAEVEHLWQNRRDGLVYVMGRADLNPAKGASWFRRLMLEWYRLLKAFSFAPDRECHIPHNRLL

>ColorbHAK3

MADQEHPYRKSSPAQGSSSSQTPLLKSPLATPISSASSDDILLASMRKDGSVRAGGNIRSAGSRPRLPRLPVSPRNMVGR

TSAADEEEAYFEPREYESHKPTGVLATIALAYGSIGVVFGDLGTSCLYTVAGIFAKFDTTPKEEDILGAISLIFWTITLI

AVVKYVFIVLFADDNGEGGTFALYSLLCRHANVSLLGNPSTSKEVAKRLRRKNSVRSISRNVLERSKPLRKTLLIVVLLA

TSMVLGDGVLTPAISVLSAVQGVQVAGPKLSTDMVLTITCIILAVLFLFQRFGTARVGFIFAPITILWLISCCSIGAYNI

YHYGFGFGVHGMGIFQAISPHYGFNLIFNHGQAGFTCLCGVILCVTGTEALFADIGHYSRSAIQLAFAGFIYPCLLTTYF

GQGAYLCRNPEHVSDTFWKSTPHTVFWPVLVLATMASVVASQALISGAFSLVKQSMSLSCFPRVKVIHTSESVVGQIYIP

EVNYIIMTLTIAVVLGFQDATKLGNAYGLAVASVMLLTTSLTALVMLIVWNKGIFLTLLFLITFGTIEGAFLAACLPEIP

EGAWFPLALAVVFLVVMYAWQFGATKKENYDILHKVRLSQIMEFISRYEVARVPGIALHYAETVSGVPPALAHSLTTLRS

FHRVLVFITVKHVPVPSVCKEDRFEIKRTAMPGAYRCLVYYGYKEQIDHGIEFVDDLVFHIVEHVKEEGYPILKKALKPG

EAPSPEMIEATNYLNLEVENLWQNRRDGLVYVMGRADLEPAVGAGWFRRLMLEAFRLLKAFSFSPDRMCQIPHNRLLEVG

MVYDV

>ColorbHAK4

MDNTKLWVNDDLREKEEKHLAKKAGWMGLLMLSWSSMGVIYGDIGTSPLYVFSSIFIDHDPERLTDHEILGAVCLIVWTI

TIIAILKYCLIVLLADDNGEGGTFALYSLLCRHTNITPANGGGIEASDLEVRMYSSSANILEKKTVVGGILRRWLSNSQW

AQTCLLVLVLIMTSMVLGDGVLTPAQSVLGAISGLKVKVDSISTGNVVLISVVIIIFIFSSQQFGTSKVGSSFAPIVMFW

FMGNLIISIHNIAKFYPSVFKALNPYYGYRFFEIRGYEGWRNLSGIFLSVTGAEAMYADLGHFSRASVQISFIAVAYPAL

VMTYLGQAAWLLGDLNRAQVSTTFYSSIPFGDAFFWIMFISATSAAIVASQALISGSFSIVQQSVALGCFPRVRTIHTSK

EVTGQIYIAPVNYVMMLLTIIVIAIFKDTVELGNAYGVAVSSMMVGTTVLMFITMLVVWEFSLFFSIVFLLVFGFIDCCF

LSANLFKVPLGGWYTLVIGVVVASMSYLWHWGVVTKRQALFADKTKLDSLLAVPEDDTSGSLISAITQEPLSRCPGIALM

YSDMVFGAPPIMRQLIARWGTVHTITVLTTIRQIAVPSVDPDERLLFRPLQFPGIFRVVARYGYMDLVDNGPTFVESIAR

KIQEVNPSLLADFTDPGMLQDKTVYILSRISTNPKANSNFLRRSLLNCYSLMARFAVQAWEEWEIPKPALFEVGMIADI

>ColorbHAK5

MDATHGGLWVNDIDLQDKKISHLAKKAGWMGTLMLARSSMGVIYGDIGTSPLYVYSSIFLDHDPSRITNDEILGAACLIV

WTITIIAIFKYCVIVLLADDNGEGGTFALYSLLCRHTSTTPANGGGIEASDMEVRMYSSSTNVLDVKTSVVGGALRRWFG

SSKGGQTCLLVLVLIMTSMVLGDGVLTPAQSVLGAITGLKVKVNDFSTSHVVAVSVVIIILIFCSQRFGTAKVGSSFAPI

VIIWFLGNIIIASHNISKFYPSIFKAINPYYGYRFFEIRGYEGWRNLSGIFLSVTGTEAIYADLGHFSRASVRISFFAVA

YPALVLTYLGQAAWLLGDANRAEVSSTFYMSIPFGDGFFWAMFVSATAAAVVASQALISGSFSIIKQSVALGCFPRVQTI

HTSKEVTGQIYIPSVNYVMMVLTVAVVAIFKNTVELGNAYGVAVSSMMVGTTVLIFFTMLIVWDLNIVFALAFLLFFGFI

DLAFLSANLFKVPYGGWYTLAIGAVVASISHLWHWGVVTKRNALFDSKINLNALLAKRDSNGGDGALLSALTREPLARSS

GMALMYSDMVFGAPPIMRHLVMRWGTMHKITVLITIRQIAVPSVDPDERLLFMPLEFPGLFRVVARYGYMDLVDNGPAFV

ASIARKVQEAHPSLLADMTDAGMLEDKTVYILSRISTSPKPDSNIFRRYLLNGYALMARFAAQPWEDWEIPKPALFEVGM

VADI

>ColorbHAK6

MDVTVTESDKVGENEPAASNHSSLEIDSAKQHGDDDSSLASEGNSVSRRKRGLLKMILGRVNAHDWEEDKDLNLEGHTHR

RGQKGYWRTTFFLAYQALGIIYGDIGTSPLYVFAGVFEKEAHGDGPASDDVLGATSLIIYALTLIIAVKYALIVLRADDH

GNGGTFALYSLLKRSGDFRTFGRATNVDKIGRKLSEPSLITTTPRDWRGHLLQSKTAQRIIRTLVVIGVGAIMGDGVLTP

AITVVSACEGIMVPVPSFSRNAVIGLALAIISGLFLIQRFGTEKVGITFSPIIVIWFLFNTVIGIINILRFETSVFRALS

PAYIVKFFMRNKMHGWRMLGGVLLAFTGAEALYADLGHFNRDAVMISCLGLVYPSLVITYLGQAAYLLHHPENVGRTFWK

SLPNEVFWPMFASATLAAIVASQALISAVFQIVYQAIRQDFFPRFQVIHTSEEVIGQVYVPVINYMLMALTLIVVSTFKS

SQKLGQAYGLAVIIDMLLTTHFITLVMICVWHVPLYFGIPFYMFYATVEGVFLSSAAEKIPNGGWFSLLMASFYAGIMLL

WFAGQKGKREYLRKRAVLFQDLLVAGKESGPGLQEKVYLQEDEVEVMRCPGIGIYYNESVTTVPPVLIEQLRAYHTAHQI

CIFMTNRHVSIPHVDPRDRVLINQLGVTGFYHAVARYGYMDRVEQGPHYVHELLVRVQHLLLRALFHCLATSEGLRRAVL

TERQTAVLGLTSNAGIKDTAKALETLDTIEAHMNDLAPQISADDTMHLGNNMQHTMTAVSGKELNNLLNLGRVSPMMREV

LLVVAEQLVAEKGSPSTVQGKPNVSKMDRALNLAYEIKVVQTAQRNEDVHYVFARSYVVPSTKGTSWMKKYLLEMPFTVM

VNNFAEDPYRLFNVPVERVIEMGLGYEV

>ColorbHAK7

MQRSRLNQVSFIAGSGESGKKDQSPRAGLSAELRAAGFRSTSAVSSPGVSEAEVKKAEAQEHLEDVKHRIEAEGGSKQFA

LLTLQALAVICANHGAAPLILFRGIFAEYEEPPSTDELLGAFSFVFWTYTLFGLIKNCHINCTADDYQEGGAFAAYALIC

RHANINGFFAGVLSPDETLRVLPKSKLDREKKSRSKDYVRKALEGSPLLQNLLLFLVIIGVGIGVTDCILTPVACAFTAV

EVIQEKFSNVGHTEAKVITCAILAITFASQKYGGSKLGNAFWPVILLWFILNAAIGISNMHKHYPGVLKAIEPHYAVDFL

SSTGPKTVGVVVGTFLVINGVEVVYEDLGHFTKDATKASFTLIAYPCLVLTYAGQTAFMIKHPHHIADVYFHSAPEYLTW

LLFALGIVIGVAAQQAVLTGFSSNMKQAWKMGALFNINVKHTSPTVHGQIYVPEMNNILFVLCLGLTIVHTDLLQLFYSY

NLPVVLLMLVTTLLLFVVMLVVWGTNPVFAFAFLIFYGGGELVFTYCLMKPLATTSSIYVYLCIFLVVLLFGLMYFGNSR

VLHFKMQNRVRVDQLGSFVETLGVQRTRGVGLFFTELVCIPPIFNHFVHNLPSIHEVMVFVAIREVARPSIDPRHRVKIH

RFEIPGFYLAVVRMGYWESPEHHDAVTIAAQVLDEIEACVVDRTGSRISLGNTGYDTPVSTERRTLGLSPSFGAEEAQSS

VAAINWSPIPTLPGPSHLPEAKLDEAIVAREEEILSYEDESEDEEEMDDAGREGGTEVSEGIRRTASDGALSELGWGKFS

RSFKGWQNFTSSALHLMAKPRDGGNQRNSGSGILVQLASFNAGRPWSTSDISRTQQEPLIRKLTSAFMRRGSLQSDSVRR

PPQKALKRMSTPPRMQNDSNTEAAAAAPGVRRRPSRTSTPPPLQRPHVSPTTSEPAPLSGSDSPFDGIPFRFADSPEASD

GEGILEAVECSLGEIVSSSSGGQTTPERSNSPPPP

>CrHAK2

MKPATSAPAEERDEEGLGGITFTKKKETIAVTAALAWGSLGCIYGDIGTSPLYVYSTIFATSEPSQADILGAISLIFWTL

TLIVLVKYVGVVLLADDEGEGGTFSLYSLLCRKIGIRPHDVMFRGESRMMRHLGSSSTGGSQLRRTSGSGTRGAGLRASG

DGTLRRLSTTPQDTARGPDTTLTLATSASRVAGTVAPHPHPQQPRRVWWRRWAASGTAVRAALRRNRAAQLGLWGMTMAA

TGMVLGDGVLTPAISVMSAVSGLKEATDAVTQQTVVGVSIAVLVLLFSVQRCGTSKVSSTFAPIVALWLCSNAGVAAYNL

ALHGGGALAGLSPHHIPLFFARRGVEAWRMLGSVMLCVTGAEALYADLGHFTHRSVLAGFCLFVYPCLVLTYVGQGAYLM

SRPEDVTDTFWKCVPRPFFYPMLVLATLASVVASQALITGCFSIISNAIKLGAFPKLSVLHTSEHVRGQVYVAEINWTLM

LLCIGVVAGFQDTVALGLAYGLAVSSVFVLTTLLILVVMVAVWEVSLALAAPFALVFLIIELAFLSANMAKVPEGAWFSL

AVSAGGIYVMTIWWVGSTRRALLLAASAGRNRLSELFVMMPLQPQQSHMAAPAAQVPQPQQVRREAPRAIAESLEDGQQE

AGQEEGQEMQGAAHSSTGADAAARTTTAATPQPAAAPGASSGGGLLHPEREIVTASNTPSSGTHGSATPADGGAIGPLPS

RRRTAATSAFAAAAGISGRSDGNAAVPTSNLHKSDSLSRGSPAQLLAAVSARLSMWRPVQLALRLPPQPLLGADGSAAVG

SQGQGLGLGADGAGAEQQQQQQLWPLSRQPGIGLYYSETPVGLPHVLIHFLRNVQSVHDVSVFLTVRVVPLPHVQPVERL

LVRQLAPFPNFYQVVARYGYMDRVDHGAAFIRQVVTGIVRVLRGGPAVAADVTTVAATGGGNGADTATAAAAAAAEDGIA

GSGSGSSGGGAGGFRFVTRNRRSNGHRGSRLSAAASRRGVNGVSAPAGEEQEQDGGQAGQQPQQPQGGWSLYGMGLSRRG

GMVLQPGEVDVSSSSSSDRGYNSAEEEGSDPDDEDEVEAVPGVPAAVAHSSNVSGGHGGTAGGNTGGTGGVELSEVVVVA

AHTAEVRADHAPAPQPPAALRSPSTGAPPPVSLPAAPAALQASRSRPRLARAARGVSSQLPSGGRVVVDEAAVAHVLEAA

RHGVVYYLGAVRVRPEPGSPLLAQLLFGATYRLLLGLSRSEVEDWRLPYEHVVELGMVLRIG

>CrHAK1

MKLAASAPEERDEEGLGGITFTKKKETIAVTAALAWGSLGCIYGDIGTSPLYVYSTIFSSSEPSQADILGAISLIFWTLT

LIVLVKYVGVVLLADDEGEGGTFSLYSLLCRKIGIRPHDVMFRGESRMMRHLGSSSTGGSQLRRTSGSGTRGAGLRPSGD

GTLRRLSTTPQDAAAARSPDTTLTLATSASRVAATAAPHPHPQQPRRVWWRRWAASGTAVRATLRRNRAAQMGLWVTTMA

ATGMVLGDGVLTPAVSVMSAVSGLKEATDAVTQQTVVGVSIAVLVLLFSVQRCGTSKVSSTFAPIVALWLCANAGVAAYN

LALHGGGALAGLSPHHIPLFFARRGVEAWRMLGSVMLCVTGAEALYADLGHFTHRSVLASFCLFVYPCLVLTYVGQGAYL

MSRPEDVSDTFWKCVPRPFFYPMLVLATLASVVASQALITGCFSIISNAIKLGAFPKLSVLHTSEHVRGQVYVAEINWIL

MLLCIGVVAGFQDTVALGLAYGLAVSSVFVLTTLLILVVMVAVWEVSLALVAPFALVFLAIELAFLSSNLAKVPEGAWFS

LAVSAGGIYVMTIWWVGSTRRALLLAASAGRNRLSELFVMMPLQPQQSHMAAPAAQVPQPQQVRREAPRAIAESLEDGHQ

QEAEQQEMQGAAHSPAGADAAAACTTTAATPQPAAAPGASSGGGLLHPEREIVTASNTPSSGTHGSATPADGGAIGHLPS

RRRTAATATSAFAAAAGISGRGDGNAAVPTSHPHKSDSLSRGSPAQLLAAVSARLSMWRPVQLALRLPPQPLLGADGSAA

VGSQGLGLGADGAGAEQQQQQQLWPLSRQPGIGLYYSETPVGLPHVLIHFLRNVQSVHDVSVFLTVRVVPLPHVQPVERL

LVRQLAPFPNFYQVVARYGYMDRVDHGAAFIRQVVSGIVRVLRGGPAVAADVSTVVAATGGGTGADTAAAAAAAAGDGIA

GSGSGSSGGGAGGFRFVTRNRRSNGHRGSRLSAAASRRGVGGVSAPAGEEQERQGGQAGQQPEQPQGGWSLYGMGLSRQG

GMVLQPGEVDFISSSSSSSDRGYNSAVEEDSDTEDEDEVEAVPGVSAAVAHSSNVSGGGVGTAGGNTGGTGGVELSEVVV

VAAHTAQVRADHAPAPQPPAALPSPSTGAPPPVSLPAAPAALQASRSRPRLARAALGVTSQLPSGGRVVVDEAAVAHVLE

AARHGVVYYLGAVRVRPEPGSPLLAQLLFGATYRLLLGLSRSEVEDWRLPYEHVVELGMVLRIG

>CrHAK3

MKPAASEPEERDEEGLGGITFTKKKETIAVTAALAWGSLGCIYGDIGTSPLYVYSTIFSSSEPSQADILGAISLIFWTLT

LIVLVKYVGVVLLADDEGEGGTFSLYSLLCRKIGIRPHDVMFRGESRMMRNLGSSSTGGSQARTLPRASGSGTRVAGLGV

NGDGTLRRLSATPEDAARGPDTTLTLATSASRRFAGPAAPHPQQPRRVWWRRWAASGTAVRAALRRNRAAQMGLWGMTMA

ATGMVLGDGVLTPAISVMSAVSGLKEATDAVTQQTVVGVSIAVLVLLFSVQRCGTSKVSSTFAPIVALWLCANAGVAAYN

LALHGGGALAGLSPHHIPLFFARRGVEAWRMLGSVMLCVTGAEALYADLGHFTHRSVLVGFCLFVYPCLVLTYVGQGAYL

MSRPEDVSDTFWKCVPRPFFYPMLVLATLASVVASQALITGCFSIISNAIKLGAFPKLSVLHTSEHVRGQVYVAEINWTL

MLLCVGVVAGFKDTVALGLAYGLAVSSVFVLTTLLILVVMVAVWEVSLALVAPFALVFLIIELAFLSSNMAKVPEGAWFS

LAVSAGGIYVMTIWWVGSTRRALLLAASAGRNRLSELFVMMPLQPQQSHMAAPAAQVPQPQQVRREAPRAIAESLEDGHQ

QEAEQQEMQGAARSSTGADAAAARTVTAATPQPAAAPGASSGGGLLHPEREIVTASNTPSSGTHGSATPADGGAIGHLPS

RRRTAATATSALAAAAGISGRSDGNAAVPTSSHPHKSDSLSRGSPAQLLAAVSARLSMWRPVQLALRLPPQPLLGADGSA

AVGSQGQGLGLGADGAGAEQQQQQQLWPLSRQPGIGLYYSETPVGLPHVLIHFLRNVQSVHDVSVFLTVRVVPLPHVQPV

ERLLVRQLAPFPNFYQVVARYGYMDRVDHGAAFIRQVVSGIVRVLRGGPAVAADVTTVAATGGGTGADTAAAAAPAAGDG

IAGSGSGSSGGGAGGFRFVTRNRRSNGHRGSQLSSRRGVDVVSASAAEEQERQGGQAGQQPQQPEQPQGGWSLYGMGLSR

RGGMVLQPGEVDFVSSSSSSSDRGYNSAVEEDSDSEDEDEVEAVLGVSAAVAHSSNVSGGGGGTAGGNTGGNGGVELSEV

VVVAAHTAQVRADHAPAPQPPAALLSPSTGAPPPVSLPAAPAALQASRSRPRLARAARGVTSQLPSGGRVVVDEAAVAHV

LEAARHGVVYYLGAVRVRPEPGSPLLAQLLFGATYRLLLGLSRSEVEDWRLPYEHVVELGMVLRIG

>CrHAK4

MKTEPEERDEEGLGGITFTKKKETIAMTAALAWGSLGCIYGDIGTSPLYVYSTIFASSEPSQADILGAISLIFWTLTLIV

LVKYVGVVLLADDEGEGGTFSLYSLLCRKIGIRPHDVMFRGESRMMRHLGSSSTGGRQLRRTSGSGTRGTGGLRASGDGT

LRRLSTKPQDAARSPDTLTLATSASRVAATAAPHPHPQQPRRVWWRRWAASGTAVRAALRRNRAAQLGLWGMTMAATGMV

LGDGVLTPAVSVMSAVSGLKEATDAVTQQTVVGVSIAVLVLLFSVQRCGTSKVSSTFAPIVALWLCSNAGVAIYNLALHG

GGALAGLSPHHIPLFFARRGVEAWRMLGSVMLCVTGAEALYADLGHFTHRSVLASFSLFVYPCLVLTYVGQGAYLMSRPE

DVTDTFWKCVPRPFFYPMLILATLASVVASQALITGCFSIISNAIKLGAFPKLSVLHTSEHVRGQVYVAEINWTLMLLCI

GVVAGFQDTVALGLAYGLAVSSVFVLTTLLILVVMVAVWEVSLALVAPFALVFLVIELAFLSANLAKVPEGAWFSLAVSA

GGIYVMTIWWVGSTRRALLLAASAGRNRLSELFVMMPLQPQQSHLTAPAAQQVLQPQQQQQQQQVRLGAPRAIAESLEDA

QQEAGQQQMQGAAHSSATPDAAARTTTAAMPQPAAAPGASSGLSHPEREIVTASNTPSSGTHGSATPDGGAIGYMPSRRR

TTAAATSAFAAAAGISGRSSGHAAVPTTNLHKSDSLSRGSPAQLLAAVSARLSMWRPVQLALRLPPQPLLGADGSAAVAG

QGQGLGSLGGDGAGAEQQQQQLWPLSRQPGIGLYYSETPVGLPHVLIHFLRNVQSVHDVSVFLTVRVVPLPHVQPVERLL

VRQLAPFPNFYQVVARYGYMDRVDHGPAFIRQVVSGIVRVLRGGPAVAADVVTVAASGGGTAGADTAAATVAAAGDGIAG

SGSGSSGGGAGGFRFVTRNRRSNGHRGSRPSAAASRRGVNGVSAPAGGEQEREGRGQAGQQQPQQPQLPHGGWSLYGMGL

SRRGGMVLQPGEVDFVGSSRSGSSSDQSYNSAEEDSDEDEDEVEAVPGVPTGAAHSSNVSGGHGGTAGGNTGATGGVELS

EVVVVAAHTAEVRADHAPAPQPPAALRSPSTGAPPPASLPAAPVALKASRSRPWLSSQLSSGGRVHVDEAAVAHVLEAAR

HGVVYYLGAVRVRPEPGSPLVAQLLFGATYRLLLGLSRSEVEDWRLPYEHVVELGMVLRIG

>CrHAK5

MPAQPASAAAAAKQPEQQQQPTQTHRAGQQQQKQHHPHMISPHPGPGGPQGGHGGGHGAALGWGAFAGLAWASVGVIFGD

IATSPLYVFTSVFTELRSTAGGGPGMPDRADVLGAACLVLWVLTAQAAKYCGVVLAADDRGQGGTFALYSLLCRHMRIKP

HGARGPPPPPPPAAAAAVAANGSAAAAAEAAEQKQQRQDQWRRRRHMRWWQWGCADAEAVRGFFRRSRGAQLALWGVSVV

ATSMLIGDGVLTPSISVLSAVSGLRVAVPALSQEVVVGVTVALLVGLFTAQQAGTGQLGVVFAPVVALWLAVNAGLALYG

MATLPGGWGVVAALNPAHGVSLFARHGAAAWRCLGGVMLAVTGTEALFADLGHFSAGSVRAAFLGLAYPCLLLTYLGQAA

HLLHRPSDWTDVFWKAAPQQLLVPLLVLATAATLVASQALISGVFSIVRQAMLLGAFPTVRVVHTGGRRVEAATQVYVPL

ANAALLLLCVGCVSGFRDTVALGKAYGLAVMSDMLTTTALVTLVMLAVWETSLALVALWAVLLLVLEGALWSANLVKIPE

GGWFTLAVAAAVASVMLVWWAGSRRVAERMAAAAAGARLRAVPRRRRRQGPPGLGPPQEGAGGGGGGGDGKLGLKGGLGG

GGGDHEGGPAARPLLLPPPPPLLPPYGHILRVSSDRQRPEPSGGGGARGVVAGEESPRLGQQRQQGPDWDSNDTAASSDS

HPLMHAAAAAAATAAFGDTGGGGGSPVGQLTPPRPLPRQQPLQPLLLQPAAVVDSQLQSTPTYLPAPLPPPATATAVPAA

TAPRAATAAGAGATASGRLLGCSSYSPPAMSQPPRGRQEHRLLAARSAAPAAAVAAAAAAAAAAAGGGSGGWAAVRRRFM

APTRLSPPPPPPPPQQQQQPPFQSQAQAHAAAARAAAAFLLSPPATAAIKAQLQQQSQQQRERAGEDAAEGAQPPVPLLQ

PPLLGRSVSLAVAAARSLAAASTGGLGGGGGIRRRSVNLQQQQQQQAGGGRGGGGGGGGGGGGGGGGGGGAGDGDSDGGG

GLGGSSSRALGGPPSGGGCVALAAPQPQPQSPDRRLLLRLGSRAEQQAAAAAAAAAAAAAVGDLGLGLGRTPDMPPSLPP

LTERQQQQQLGYPATAAPDPAPASNGAATASGSDYDYGYDLVLELPSAHPHTQHQQQRLQQQQQQSAAAAVPAAYAAAAA

AAAGKPPPPPPPPAEGGVSAALSPPPPPPPPLSVRLTVLEGVGIYYMDELTPQQQPEGAAALPPVLVHFLRNVQAIQAVG

VFLSVRQLPVPSVPRSRRLEVLLPHSGAAGSGADMGVGSCLPPNFYRVISRYGYLDQVDHGPAFLSALCGALLQQLLARG

LQAAEEEEEGEAEEEEEAGEEEAGEEEEEGEAAAPAAVRLGGSGRGRDGDSDVGSGDGSGGEDGEDEDDDEGEREAAAAD

AAEGGAGRWLFCPQAHPHPHPRYHTPHYPLHGGPQRQEVGQEEEELEVDAADEAAAMMAAGDGGGGGGSSSGEHGEGNEA

AAAAAAARPTRSRGPLQRRGHGRIHGGGSSSSSMWPMRIASATAAAAAAPTSPATAAVASAREGQQQAPQPQPLQQPQPP

PPAHDRCRRRRGHVRPPQSGQQWGVAEVEAAVSEFLRARLHGVVYFTSRPQLRTGGAAAIRAPPRPLLLHPTPPPRPTPP

PRPPPPPPPGSCAATAAPSCPSPPAPMGPRRLLQRLRAGLVWWLAAVAYDDSERWRLPGEALVEVGMAIEVD

>CeuHAK1

MKSSSFKGTKEEAKREEVWARDTDLVAKMSAVQAERSTTRSILFLAYGSLGVIYGDIGTSLIYVFASVFSTLAIGGGDYV

ADYAASVHDDVFGAMSLIFWTITLIAVFKYVLVVLRANDTGEGGTVALYAQLCRTMGFSPFGTLRKDDHEQVLRMKSAIE

GPVKRPALGEHRHRELAKQQSIPKGKKRSQPPCFLPNVPPFGYWAVDPAPIIQWFRRHRGLQVLLMFFASVATGLVIGDA

ILMPAFSVNSAISGIRDNASTNISNDSIVGISISIIFVLFILQHIGTRKIAWMFSPIIIVWLCANFAIGVFNMHFYGGTI

WGALNPKWIGIFFQNHGVSAWEALGGVMLCVSGTETMFAAMGHFSQPAIAVAFGLFAYPCLIVAYLGQGAYLLVYPGDVS

ITFWASLPNSVYWPMVALATLAASVASQAVITGTFSILRQSMSLGIFPKLHVIHTDSFVEGQIFIPVINYLLMIICIAVI

AGFGGDNVQLGHAYGVAVMMVMLITTLLVSLIMIVKWRVSIFLVVPFFILFFCIEGIFLSANLFKVPAGGWFTLVVAFVV

ASIMFIWWAGSSARRKVLLHTSKQQAANNLFIQHNPSSESRHNLMTFAQRGVVAQQEADAVTLTAAGYAVGDGPSFALQH

SIESKYSFPILSQSAPKSSGHMKNAGPEGTLAKGPKPSLSRFSDVSGANKTLRPPHSNGNHVSWQQQQHVPGTGATAPPG

SGEDVDPEAPKTVFATAAAASILHGAGASVDSNGIHITLSPSTGLPGIQKSVSPSVTISTGESPTAQSSPRQSMHRVQSQ

LALHNRPERPLAKLMGVGIYYSESPVGVPPVMSHFLKNIDALHGVAILLTVRFLPMPTIKNVERLLVRAVSGVPNFYQVV

ARYGYQDHVDHRSQFVSQVISTIMHKLELKAGLKYQGLEDDFIAELEGDHVDDHDHDTAHNADMVFGLNPVHEDILDEEN

LARQSIAAEVVGASMLVSQFSKNALDRIRLAAAVSDAPQSAKDALEAAETLIHSASEQAVYYLGRAFSRAAPGSNFLHNF

TIGGLYRFFEDLSYNDSEAWNIPLEEMVELGMALEI

>CeuHAK2

MSDGAKLPIIRKSASAIQRARFYNDEDIKHKIDEIEENKHSWRSTLFLAYGTLGVIYGDVGTSPLYVYSSTFTDFIPEQD

DILGAISIIFWSLTLVALIKYVFIVLQANDQGEGGTFALYTRLCRSLGISASGSVTKKEEHEEAIQTAAAVGTLSRATRK

NSLNRNTIGSTLHSAMIRKGSGLQDNEEDGEDERLSTGNKTSARVPPIKYSSTSSLSARQSPSASSFIKRLNHLAHSLPF

PREAVMHFFQASSRVRLTFMSISIIATCMVIGDGVLTPAISVVSAVSGLQQQVTISQGGIVGISSGILISLFIAQSMGTE

GVAMFFSPVVAVWFVCISSIGIYNMSLYGANVWSGLNPYWIYVYFNNHGYKAWKSLGGTMLCMTGTEAMFADLGHFSIPS

IGISFSFLVYPSVMLSYLGQGAYLQVYPENVSTAFWSAVPNQFQWPMLVIATLAAVVASQAMITGSFSILRQAMTLGVFP

KLKVVHTGEHVEGQVYIGVVNYTLMLLCVAVVAGFNADSTSLGNAYGIAVSTVMLITTNMITLCMIVDWKLNACLITFFW

CLYSLIEGAYLSANLLKVPEGGWFTLAITAGVALISFGWLSGQTAKKEALQSAQQQVSLNDIMSVPDGSDVADLGTLTLL

PSSSEHHLGATSIGTQGGHVSFSTGVNISSPSVPLSVFASKAAQNLATFSTRAASGGVAAAAAISRQHQALSTFSTGHAL

AIKSSRKLGSDLLPTLSSSSTSSAIRRVHQEQAVLPGSSSAGVTGHKSRFGPSLGTGSLGSRVGSSFKAKGESDLTVPGH

PPLKTVSEGDDEVTRQSIMRREESLLRERKKATGTDASGEAGINNNKPSHPGPLGESRESGGTAAPIRYSTRAGPVAEAR

VPGTALPGTGSTRSEDEAPAEDLEAVMSGSAADMGSHMHTSDVAEHQTLLARLRGIGLYYTDSSHGIPPVMWAFLRNVEA

VHEFAILIQNRFLPIPYLTDDERLAAVPVRGIPNFFRVVARYGYMDKVDHGPEFVQSVIDYISGQLECVVDPSKIIRTQK

SEALVSVALAGVPEDVPMIPIINETSTQHVLKTLSNAAMQPSLSSSASMQGRRASVHMVLDSQDPLKRAAAAASLEVLRC

AAEEGAVYYLGRTNVQMKEMPKGISKMLHDVWYGDFYRFMALNCWTQTEAWNIPNKSLVELGITVDI

>CeuHAK3

MSSNPVYSERGRFYEDADIQLKIKDANKKKLSWRSTIFLAYSTLGIVYGDIGTSPLYVFSSIFSSFSPVDNDILGATSMI

FWSITLVVLFKYVFIVLRADDQGEGGTFALYTRLCRSLGINAQGTVHKHEEHQEVLQAASVMGSLDRKSRKTLLSQTLED

KDATHVAVSDRTLQQRIPSGLPALSTPLVGKKGSASSNQVLQFLSNACHWMPFPHEPVQRFFQSSAVARFSILSVSILAT

SMIIADGVLTPATSVTSAVSGLTIQVAISQNAITGISCAIIIILFLAQSFGTQKVSFTFSPVVVVWFMSIGGIGIYNMTL

YGTYIWNALNPYWIYVFFSSYGYNAWVALGGVMLAVTGTEALFADLGHFSRPAIGIGFSCIAYPSLILAYLGQGAYLIQN

PGNVSSAFWSSVPTQYAWPMLIIATGAATVASQALITGSFSIIRQAISMGIFPKVKVLHTGKEVEGQVYIGFVNYVLMIL

CVAMVAGFNGNGTAITNAYGICISIVMFITTNLITLLMVVDWHLSSVLVTLFYLLFGLIEGAFLTANLTKVPQGGWFTLA

VGAGVALVKFGWLSGQIAKKEAIQEIFNTLKLSDLFSAKSESNRGNEPFISGGDVPLNAFSTPLLKPGPEVRTLVTMSRQ

RALKTTSTGGPLMTISRSGRLGSDLLSTTSVKLRQASALRKGQQVALAGSSTTRFVPSLASQSVGNRVGCAIAMGAVDST

HAADSPANMSTVLENSSDHKGSKHTALLPTSSAGHHLNSDAVVMEVLHDSVADLDNLINSKDLAGCQVLSKLRGIGLYYT

DQTHGIPPVMWTFLRNVEAMHEFVILLQNRFLPIPFLAEEERLAAVPVLGVPNFYRVVARYGYMDKVDHGSEFAQSVVDF

ISGQLDGIANPAAHAPNLPPLSTASMMGRRTVVQARLADEADAADRQAAAEELVALRHAAKEGVVYYLGRTNVQMKDLSN

QFTQALHDLWYGDFYRFMVNNSWTESEAWNIPGESMVELGINVKI

>GpHAK1

MASTALELMDDADLTKRAPPRRAAWSTTLALAFGALGVIYGDIGTSPLYVYSTIFSKLDPSKNDVLGAVSLIFWTITLIV

LVKYVGVVLLADDDGEGGTFSLYAFLCRRIGIRPHSGTAKEDSRIQRGLSTARGTTLIARSSLHRQGPAPWWRQLSANGS

TVRGALRRSRAGQLSLWGMTVVATGMVLGDGVLTPAISVMSAVSGLKEATPAVTQHAVVGISIAILVLLYSVQQLGTHRV

SFTFAPVVALWLTANLGVAIYNISRYGGAVFAGLSPHHIVLFFSRHGTEAWRMLGGVMLCVTGAEAMYADLGHFSHGSIV

LSFSFFVYPCLVLTYLGQGAFLMARPEDVGETFWKSVPRPFFYPMLVLATLASVVASQALITGSFSIVANAIKLGAFPKL

HVQHTSASVRGQIYVGEVNWALMLLCIACVAGFQDTVALGNAYGLTVSSVFLATTALMVLVMVAVWELSLLAVLPFAAVF

LLLEGAFVSANLEKIPEGAWFTLVVSGGVSYIMIICESSDSATQQQQPQQQLLQTSSSGGNAAVNVAAGPSAHAAASASG

PASVGLEATAAAGARPAPNTDATGGAAAVLATAAAGPPTLMAVRAMTLARLSSWRPTELAMRQPSGELRLLTRQPGVGLY

YSESPVGLPSVLLHFLRNVHSVHDVSVFVTVRIMPLPHVQPRERLLVRQMTPIPNFYQVVARYGYLDKVDHGPSFVTCVV

DAIVRQLRRGGQTRHQAQRWEDVRVQVGVDHDVDEASDDEEAGQLPPLPVLPPDSGSTTAAARGTAAAAAATEINASVVS

APSPVAAPVLSPSGGSADGEDSLRRAGGAVGRSRQPPLPAAAHSAGAVDQAAVAHILEACNQGVVYYLGVTRVRPDPSSR

VLHQLLFGAVYRLLLGLSHSEVEEWQLPEESVVELGVTLRI

>PaHAK1

MELGAQIASKEAENLEMEEMANGVENEDGGILRGNKLHADSHGSRFKRVDSLDLESSRVSGMGTHASNLVTVTSILHLAF

QSIGVVYGDIGTSPLYVFASTFTDGISHPDDVLGALSLIVYSLTLLPLLKYVFIVLWANDNGDGGTFALYSLICRHAKVS

VVPNQQAEDRELSSYKLAVPSKELKRALKIKDALEKSDAAKTILLILALLGTSMVIGDGVLTPCISVISAVQGIRNVDNS

ISQEMVAMISVAIIVILFSVQRFGTQKVGYSFAPAILIWYSFIAFIGIYNIAKYDTTVFRAFYPKYIFEYFRRNSKQAWI

SLGGIVLCITGTEAMFADLGHFSVRSIQVAFTGIVYPALICAYVGQAAYLRKFPENVLDTFYKSTPSAVYWPMFVVAIVA

SIIASQAMISATFSIIKQSMSLGCFPRVTVVHTSSKHEGQVYIPEINYILMLACIIVTASFKDTTKIGNAYGIAVVGVMI

VTSSFLTLVMLMIWQTHLLLVVIFVTVFGAIEFTYFSAVLYKFPQGGYLPLAFAAVLLFVMYVWHYVHVKRYAYEVEQKV

SSEYILSLGSSLDVTKVPGIGLLYTELAQGVPAIFAHFMRNLPAMHSVLVFVCVKYLPVNTVAAGERFLFRRIGPKEHKM

YRCMARYGYRDTRTGNVEFENQLLESLKEFIQFDYTGTYDNNKQPQNEGNSFDSGETIVNIGAATENEVMSACTMVNVEE

ELDFLENAKKSGVVYLLGDSEVIANKDSSMFKRFIVNYAYNILRRNCRKGQAALEIPHKNLLQVGMTYYI

>PaHAK2

MAATAVCAIKRYHPQLQDLWHLTTPPYWKVKKMEGAQMASREEENTNLPQMASKEEENMELSQMASKETEEMVGVHNGGG

NKLQTNSHGERLKRIDSLDLESSRVSGMASHGSNLLTMASILQLAFQSIGVVYGDIGTSPLYVFASTFTDGIRHPDDVLG

ALSLIIYSLTLLPLLKYVFIVLWANDNGDGGTFALYSLICRHAKVSVVPNQQAEDTELSTYKLAIPSKELKRALKIKESL

EKSNAAKTILLILALLGTSMVIGDGVLTPCISVISAVQGIKTVDKSLSQEVVAMISVAILVILFSVQRFGTQKVGYSFAP

AILIWYSFITVIGIYNIAKHDTSVFRAFYPKYIFDYFRRNSKQGWISLGGIVLCITGTEAMFADLGHFSVRSIQIAFTGI

VYPALICAYVGQAAYLMKFPEDVADTFYKSTPNAVYWPMFVVAIIAAVIASQAMISATFSIIKQSMALDCFPRVTVIHTS

SKYEGQVYIPEINFILMLACVVVTAAYKDTTKIGNAYGIAVVGVMIVTSAFLTLVMLIIWQTHLFLVVIFVIVFGAIELV

FFSAVLYKFPQGGYLPLAFAAVLLLVMYVWHYVHVKRYAYEVEQKVSTEYMLSLGSSLGVTKVPGIGLLYTELAQGVPAI

FAHFVRNLPAIHSVLVFVCVKYLPVNTVPAGERFLFRRIGPKEQKIYRCIARYGYRDTRTGNVEFENQLLEFLKDFILSD

NIYTYDMNNQPESEGRNCDRGESIVDIGAVSENAVMDACTMESVEEELDFLENAKKSGVVYLLGDSEVIASKDSSLFKRF

IVNYAYDILRRNCRKGQAALEIPHRNLLQVGMTYYI

>PaHAK3

MASEESEATIVGVENEGRLLKKGRVDSFDVETSRVSKMNHGGQILTMAATLQLAYQSIGVVYGDIGTSPLYVYSSTFADG

FIHHPDDIVGAFSLIIYTLTLLPLCKYVLTVLWANDNGDGGTFALYSVICRHAKVSLIPNHQAEDRELSTYKLATPSKQL

KRALKIKEALEQSYPAKIALLLLTLMGTSMVIGDAILTPSISVISAVEGIQNISQSINQNVVVGISVAILVVLFSIQRFG

TDKVGSAFAPAILVWFLFILLIGIYNLAKHDTTVLRAFYPKYIIEYFRRNHKRAWISLGGIVLCITGNEAMFADLGHFSV

RSIQISFTGLIYPSLICAYGGQAAYLSKFPDDVQKAFYKSIPTPVYWPMFVVAVLAAIIASQAMISATFSILKQAMYLGC

FPTIRVIHTSAKYAGQVYIPEINYFLMVACVLVTVSFKNTIMLGNAYGIAVVGDMIVTSTMLTVIMLMIWQTNIYLVAMY

VLVIGSTEWIYYSAALYKLPKGGYLPLAFAAFFLSIMYIWHYVHVRRYAYEMEHKVSTEHINSLFSSLSVSRVPGIGLLF

SELPHGIPPIFSHFITNLPAIHSVVAFVCVKYLPVNTVPAEERFRFRRVGPEEYRMYRCVALYGYMDVRVGNVEFETELL

ESLKEFIQSEYMSRLPEIEIRVSQRFDSSQRFENRLTANSIWPGESEDDAMATSQSRQLMQAELQSQQDIEFLQEARKSG

VVYLVGDSQVNVKNGSPLIKRIVVNYVYRFLRRNCRQGRTSLDIPHKHILRVGMDYEI

>PaHAK4

MDLGRRMASKEAEDVEMEEMVDGVENEEGGVLRGNKLQTNSNGSKLKRVDSLDMESNRVSGMASHGSNLLTMASILQLAF

QSIGVVYGDIGTSPLYVFSSTFTDGIHHPDDILGALSLIIYSLTLLPLLKYVFIVLWANDNGDGGTFALYSLICRHAKLS

VVPNQQVEDAELSTYKLAIPSRELKRALKIKEALEKSNAAKTILLVLALLGTSMVIGDGVLTPCISEVVAMISVAILVIL

FSVQRFGTQKVGYSFAPAILIWYSFIACIGIYNIARHDTSVFQAFHPKYIFDYFRRNLKQGTEAMFADLGHFSVHSIQIA

FTGVVYPSLICAYVGQAAYLRQFPGDVADTFYKSTPNALYWPMFVVAIVASVIASQAMISATFSIIKQSMSLGCFPRVTV

VHTSSKYEGQVYIPEINYILMLACVIVTASFKDTTRIGNAYGIAVVGVMIVTSSFVTLVMLMIWQTHLFLVVIFIIVFGA

IEFTYFSAVLYKFPEGGYLPLAFAALLLLVMYVWHYVHVKRYAYEVEQKVSTEYILSLGSSLGVTKVPGIGLLYTELTQG

VPLIFAHFMRNLPAIHSVLVFVCVKYLPVNTIPAEERFLFRRIGPKEHKMYRCIARYGYRDTRTGNVEFENQLLEFLKEF

IVSDYTITYDTNNQPESEGSSCEFAESIVDIEAASETAVTDLCTMESVFEELDFIEIAKKWGVVYLLGDSEVIASKDSSL

FKRFIVVRLLQAKIHRCSKDS

>PaHAK5

MDLEAGAYAHVVQKESWKTVLTLSYQSLGVVYGDLSISPLYVYRNTFAGDIEHSDTNEEIYGVLSFVFWTLTLIPLFKYV

FIVLKADDNGEGGTFALYSLLCRHAKISLLPNCQVADEELSTYNFEDFPQTKTGSRVKSMLEKCRLFHTTLLIVALIGTC

MVIGDGVLTPAISVFSAVSGLELSMSHHQYAVVPIACLILVGLFALQHYGTHRVGFMFAPIILTWLLCISAIGLYNIIHW

NPSVYHALSPYYMYNFLKKTQRGGWMSLGGILLCITGSEAMFADLGHFSQLSIKIAFTSIVYPALILAYMGQAAYISQHH

AITGAYQIGFYVSVPEHLRWPVLAIAILASVVGSQAVITGTFSIIKQSLALGCFPRVKVVHTSDKIYGQIYIPEINWILM

ILCLAVTIGFRDTKHLGNASGLAVITVMLVTTCLMSLVIILCWHKSALLAFFFILCFGSIEALYFSASLIKFREGAWVPL

VLAFIFMFVMYVWHYGTIKKYEFDLQNKVSMKWLLGLGPSLGIVRVPGIGLIHTELVTGVPGIFTHFVTNLPAFHQVLVF

VCIKSVPVPYVRPEERFLIGRIGPKEYRLYRCIVRYGYRDAHIDDHEFENQLVFSIGEFVRSERQSLSANVDFSQDGKMT

VIGTPRREDMTNDETDECMQSASASNPRSPIIQSINDIIEVNSPKPVRRKKVRFLLPNSPEMDPTVRDELQELSDAKEAG

AAFILGHSYVKAKRGSSFLKKFVINVAYDFLRKNCRGPSAALNIPHISLLEVGMVYQV

>PaHAK6

MEVAGESEHAVADSEAVVTIQKVEEGMMDKDKPDIYGTILQSYEVPPPKPKIFGRRETLLLAYQTLGIVYGDIGTSPLYV

FSSIGLENPQEKEILGCLSLIFWTLTMIALVKYVMIVLRADDHGEGGTFAVYSLLAQHVNLEKSTGKQFTRLASDSKLKF

FSKQNGGKVINSKTKELLDNSATAQRILLIVVMTGTCMVIGDGALTPAISVMSAVQGIQSKNPNMSQGIVVFLSFVILLA

LFLFQRFGTSKVSFLFSPIMIIWFVTNIMIGLYNIVKYYPGAFKAFNPYYIVYYFHKFHKQGWEMLGGVVLCITGAEAMF

ADLGHFNRRSIQLAFCVLVYPSLLITYTGQAAYLIKNPGDIGAAFYKSIPQAVYWPMFVVSTLAAIVASQALITATFSII

KQSMALSCFPRVKIVHTSKNYEGQIYSPEVNYVLMIICLAIVVGFRDGTQIGNAYGVAVVGVMFITTCIVTLVMLVVWNM

HVFLILPFTVFFGLLEGIYLSSVLNKVPQGGWCPFLIAGVFLTIMFSWNYGRQKKYKYLAQRKLSTEGFHGLVSSVNSRV

PGICFFCSDLIYGLPPIIGHYVKNVGSLHEVLIIVTIRIIPVKTVLLKERFMVGRLEPKGVYRCVAQYGYQDVPSMEGFE

FINQVVESIKDYLKSRETYSFSNQDTRCASRNTVAESNYSEHEELQQLELAKNTGVVYVLGKTTLRTNKSTGPFEGLLID

KLYRFLQNNCRSTMSTYNIPPAQLLQVGMVHEI

>PaHAK12

MDAEAGTHQKEAKAQSWRTVLTLAYQSLGVVYGDLSISPLYVYKSTFAEDIQHSQTNEEIFGVLSFVFWTLTLVPLFKYV

FIVLRADDNGEGGTFALYSLLCRHANISLLPNRQVVDEEVSTYKLEYPPETKSGSRVKKILENHKNLHTALLIVVLLGTW

LVSLDAEEITLSSSEITCHGWTIDTKYYTADVCVWMAHLCEKTSEHARSLSKHCDALVMVFDLSNPSSFEVLQDWVSEVD

LHKFEILLCIGNKADLLPSHPSHAEYRRKLQKCGESSSDPHPEFWNYGIDRSEGCGLLNEDEETSEEIQSALGLYNIFHW

NRHVYRALSPYYMYKFLKKTRKAGWMSLGGILLCITGSEAMLVSEYFRPLYNIDIEQLCLLLYAGSEAMFADLGHFSHLA

IKIAFTSVVYPALILAYMGQAAYLSKHHHIGNNYAIGFYASVPEPVRWPVLGIAILASVVGSQAIITGTFSIINQSLALG

CFPRVKVVHTSDKIHGRIYIPEINWILMILCLAVTIGFQDTKRLGNASGLAVITVMLVTTCLTSLVIIICWHKSIVLAVC

FLLFFGSIEALYFSASLIKFLEGAWVPMLLALIFMTIMYVWHYGTIKKYGFDLQNKVSIKWLLGLGPSLGIVRVPGIGLM

YTELVTGIPANFTHFVTNLPAFHQVLIFICIKSVPVPYVPPEERFLIGRVGPKEYRLYRCIVRYGYRDVHGDTDHFEDQL

ILNLGEFIRSEAKHSCSSDSYSADGRMAVIGTPVHGMESILSYETAEESIQSVNLSYLGSQTIQSIQDLIETDSPHLARR

KKVRFLLPSSPDIDPDVKDELQELFQAREAGIAFILGHSYVKAKNDSSFLKKLAINAGYNFLRKNCRGPSVALRIPHISL

LEVGMVYLV

>PpHAK1

METSAVELQMSTDAVHQRETGERPPLEQTAQDEEISREALHADEAAYREKEGPFRRLSRKLTRPDSLDVESMRVKEMDHA

APVASFSFILKLAYQSIGVVYGDLGTSPLYVYSSTFTSGIKTNDDILGVLCLIIYTIIATPLVKYIFIVLRANDNGEGGT

FALYSLICRHVKLSGAHAQQPTDLNISSYKLETPSTKMARATRIKEALEKSRAWQNVLLLIVLLGPCLVIGDGSLTPAIS

VLSAIQGISVNVSGLSPNVSVIITVVVLAALFSLQRFGTHRVAFLFGPAMLAWFFSIGIIGLYNIFRWDPSVFKALNPWY

GLNYFIRNKVDAWASLGGIVLCITGSEAMFADLGHFTVKSMQVAFTFLVFPSLLCAYIGQASFLMKNQLDDDVAYTFYRS

VPKPIYWPMFGVATCAAIIASQAMISATYSMIRNAMSLGCFPRVTIVHTSKKVHGQIYIPEINWIIMVLSITIVGGFRST

TQIGHAYGIAVVGVFFISTCLLTLIMLMIWQTNIFLCALFFTVFFIIEGIYFSAVLSKVTQGGWVPLVIAACFLTIMYSW

NFGTRMKRLYEVSHKISLDWVLSLGHSLGISRVPGVGLVYTELPQGVPAIFRHFISNLPAIHSTLVFVCIRHISVSTVPE

DERILIRRLGPRNYRMFRCAVRYGYTDHVDGAESDGQTFENMLLASLERFIRTEAAEVTPESGLASSHAASPSHHKLDRP

CESSVSNDSCGSDIGAKTVDELEADQEAYTNEEVLFLQKAREAGVVYVLGDSDIHAKSDSWFPKRIIINKIYKFLRRNCR

NNTLYLSIPKDRLLKVGMEYYV

>PpHAK2

MSTTTVSEDAEDGRGGRNGQQANQGRLWDMDQRIDQPLGAEADHVRSMYRDQTMPPSVVLCLAFQSLGVVYGDLGTSPLY

VFKSTFANGGVRNEDDIIGALSLIIYTLTIIPLIKYVFIVLRANDNGEGGSFALYSLLCRYCNISALPNQHPSDAELTTY

VVDNARRKTWIQRKLESSVLAQQVLLVIVLFGTCMVIGDGILTPSISVLSAVVGIKAASSSLDTNLVTGISCVILVILFS

VQRFGTAKISVLFAPIFLVWFLSLACIGCYNIIKWEKSIFLAFNPLQIVHFFRRNGRQGWEHLGGIVLCMTGTEALFADL

GHFSCRSIQIVFTSLVYPCLFLTYLGQAAYLVEHMEDVNDPFYSSLPSSIYWPIFVLATISAMIASQAMISATFSIVKQA

TALGCFPRVKVVHTSNNVAGQVYIPEINWILMVLCLCVTAGFRDTDQIGNAYGIAVVMVMIVTTLLMTLVIIIIWRKHFL

LALLFLVVFASIEGIYVSAVLFKTTQGGWVPLVISVVFGTVMGTWHYGTLKRYQYEMQHKVSVGWLLGLGPSLGLVRVPG

IGLMYTDLAHGVPPLFSHFITNLPAIHSTVVFVCVKYLPVNTVPQDERFLIRRIGSRAYSMYRCAARYGYIDLHKKDDNF

EQLLIQSLISFVEIESMRESSGRESMAASWTPDQQPMEEATVPTTSTITPNRLQLQRMLRLHSLMGGGNSVGDGYSTQYS

QTASNSVEMSANQECSIPNLSVNGSNSSSSPHPQDEVAFLNACKDAGVVYILGNNIVKARKDAGFFKKLVINYMYTFLRR

ISRDSSVVLNIPHECLLHVGMVYYV

>PpHAK3

MASVSFVKPFSCRWVYKRMSTGFVAVSLTTVLRLAYQSLGVVYGDLGTSPLYVFKSTFANVGVSNKSDIIGALSLIIYTL

TIIPLIKYVLIVLRANDNGEGGSFALYSILCRYCNISSLPNQHPSDVELTTYLVDNVNRKTWMQRKLENSITAQKVLLAI

VIFGTCMVIGDGILTPSISVLSAVVGIKAASSNLDTNLVTVISCLILVILFSLQRFGTDRISFLFAPIFLTWFLSLALIG

CYNIIKWEKSIFLALNPLEIVYFFRRNGRQGWEHLGGIVLCMTGTEAMFADLGHFSFRSIQIAFTSLVYPCLILTYLGQS

AYLVEHMEHVNDPFYASLPRRIYWPIFVLATISAMIASQAIITATFSIVKQSAALGCFPRVKVVHTSNNIVGQVYIPEIN

WILMVLCLSVTAGFRDTDEIGNAYGIAVVMVMIVTTLLMTLVIVIIWRKHFLLALLFLIVFASIEGVYISAVLFKTTQGG

WVPLVIAAVFGTVMYTWHYGTSKRYEYEMQHKVSVGWLLGLGPSLGLVRVPGIGLMYTDLAHGVPPLFSHFITNLPAIHS

TVVFVCVKYLPVNTVPQAERFLVRRIGTRAYSMYRCAARYGYKDIHKKDDDFEQLLIRSLIKFVEIESKRETSDLESMAA

SWTPEEQQSVASLPAMPTESSNRLNLLRLLRLHGLMGEGNSIDEGCCTEYPVSDINLATTSTYQEGSIQTQSVNGTSSDS

QDEVAFLNSCKESGVVYILGNNVVKARKDASLFKKVVINYIYTFLRRISRDSHVVLNIPHECLLHVGMVYYV

>PpHAK4

MSTTMISDDLEGGRSDDIQLQRAHRQGRLWDMDQRIDQPLGVEADHVKSMYTNKAVTLGAIMHLAYQSLGVVYGDLGTSP

LYVFKSTFANVAVTEKQDIIGALSLIIYTLTIIPLIKYVFIVLRANDNGEGGSFALYSLLCRYCNISLLPNQHPTDVELT

TYLVDHANQKTYLQRKLEGSPSLQKVLLLIVLLGTCMVIGDGILTPSISVLSSVVGIRAASSSLDTTLVTVISLVILVIL

FSLQRYGTATVSVVFAPIFMSWFIVLALLGCYNIIKWDKSVFQAFSPHEIIRFFTRNGSVGWENLGGIVLCMTGTEALFA

DLGHFSFRSIQMAFTSLVYPCLILTYLGQAAYLVGHTENVNDPFYSSLPPPLYWPIFVLATVSAMIASQAIISATFSIVK

QSVALGCFPRVKIVHTSNDIAGRVYIPEINWILMGLCLVITAGFRDTNEIGNAYGIAVVVVMIITTILMTLVMIIVWRKH

VLLALLFFTVFMAIEVVYLSAVLFKITQGGWVPLAIAVVFGTIMYTWHYGTLKRYQYEMQHKVSVGWLLGLGPSLGLVRV

PGIGLMYTDLAHGVPPLFSHFITNLPAIHSTVVFVCIKYLPVNTVPQEERFLIRRIGTRAHSMYRCAARYGYKDIQKKDD

NFEQLLIHYLTKFIEIENFREQCDLQSMAASWTPEEESVRSMPTMNSPNSSRLQRALRSNGITRAENSVGNGHLSQSCTS

LAEMPTNQSVDDNSQIQLSISGSNSDIQDEVAFLNSCKEAGVVYILGNNIVKARKDSGLFKKLIVNFIYTFLRRISRDSR

VVLNIPHECLLQVGMVYYV

>PpHAK5

MLTMLGVVLVVARVCFDLKRILGARILGRGSYSTSSAVLYPSTGDCSYGNSGNEVRWTKTCSHVDLPRIAQKASLAFVMK

LAFQSIGVVYGDLGTSPLYVYSSTFTHGIKKNHDILGVLSLIIYTLITIPLIKYVFIVLRANDNGEGGTFAMYSLICRHA

KITLDHNRHPTDRNISSYVLLKPSSRMTRAMRVKEELENSRVWQNILLVVVLLGPCLVIGDGSLTPAISDLSVIITIVVL

IGLFSLQRFGTHKVAFLFGPVMLCWFFSIGAIGLINIVRWDPSVFRAFNPYYAVSYFIRNKQQAWASLGGVVLCITGSEA

MFADLGHFTVKSMQIAFSFFVFPALLCAYIGQAAFLMKNQSMDDVTYTFYRSIPKPVYWPMFAVATCAAIIASQAMISAT

YSMIRNAMALGCFPRVTIIHTSMKVHGQIYIPEINWMLMVLSIVIVGGFRSTSEIGHAYGIAVVGVFFISTCLLTLIMIM

IWQTNIFLCLLFFVVFVIIEGTYFSAVLSKVTQGGWVPLVIAACFLTVMYSWHFGTRMKRLYEISQKLSGDWVLSVDHSL

EISRVPGVGLVYTELPQRVPAIFDHIIRILPAIHSTLVFVCIRHIAVSAVPDDERILFRRLGPRNYRMFRCAVRYGYTDL

HSESDGESFEAMLLASLERFIRTEAVEQAPDFIVGDSPGVTSPGQYSVGCDCESTSVLFDRTDQSDINFQMSQEWKRPYS

AEDLVTGHDNSTAEELALLQKGREAGVAYLLGDIDLHAKSDSGWYKRVIVNHIYSFLRRNCRRNELYLSIPKARLLKVCM

EYYI

>PpHAK6

MLTLPCAHLGHPQVRKSAAAVPGVGFEIRAKISRITTAHLAFQSIGVIYGDIGTSPLYVYASTHVLNKTDDAGNFIPALN

DDILGVLSLIIYTFTLIPLIKYCFIVLQANDNGNGGTFALYSLICRYAKINLATNQAPEDRVLSTYQLDLPTQNAKRAAK

IKEYLERSRFWKNLLLTVALVGTCCVIGDGVLTPSISVLSAVSGLKVNTPTISNDVVVEVSVASLVVLFAIQRFGTHKVG

NSFAPCICLWFACIALIGIYNIIKFDPSIFKAFNPYYINSFFKRNKRDGWVSLGGVVLAITGSEAMFADLAHFSVASIQI

SCTIVAYPCLILAYIGQAAWLMKHQDMVSTTFYSSIPKPVYWPMFVVATAAAVIASQAMILGVFSIVVQSMALGCFPRCK

IVHTSPKYEGQIYIPEINWALMVMCIIVTAALQDTAKIGNAYGVTVVAVIFMTTFFVSFIMLMIWQKNLWLTLAFFDFFG

AIELVYFSSVMYKIPQYGWIPIAFVTGLISIMYTWYYTRKEAFKYEVNNKLSMNWLLGLGSNLGIARVPGISLIYTELPQ

GVPGIFGHLISNLPAMHSTLILVCIKNLPMPTVPAEERILLRRVGPPAYRMYRCAVRYGYKDDDGRGAELEDELMSSLEE

FLRAEAAGALQLELASNPANEDCRALEDYQAGGSLVTGAHDKGRKTDHDIEIDSRAQRKIEGLQQARQNGVIYILGHTNL

RCKSESNFLRKFIIDDYYGFLRRNCRSIIDTFDIPHTNLLQVGMVHYI

>PpHAK7

MSVVSDSNAAQCSCSCRNLQTPLLQPSIAVVLTLAYQSLGVVYGDLSVSPLYVFQSTFLGDLRNSVTDEYIYGVLSLIFW

TLTLIPLIKYVIIVLSADDNGEGGTFALYSLLCRHAKLSSILNQQSADMELSIYRLVEPPETPRGRTVRKLLEKHRILRT

GLLIIVLLGTCMVIGDGVLTPSISVLSAISGISVAAPQLHQNIVILVSCIILVLLFSLQHIGTRRISFLFAPIVLTWLFC

NGGIGLYNLIAYNPSIVRALSPYYTFKFFKVSGRDGWISLGGVLLCVTGSEAMYADLGHFCRRSIKAVFILIVYPSLLLG

YMGQAAYLSKNLDDLDSAFFRSVPKPVFWPVFITATLASVVGSQAVISATFSIVRQCQALGCFPWVKVVHTSNSIYGQVY

IPEVNWIMLILSLSITVGFKSTIEIGNAYGIAVIVVMLVTTFLTSLVIVVVWQRSIFVACIFLLIYGSVESLYLSSALFK

VPQGGWVPLVLVGILMCIMYMWHYGTTKKYKFDLQNKVSMKWLLTLGPSLGVVRVPGIGLIFTELVTGVPAIFSHFVTNL

PAFHQVLVFVCMKSVAVPFVPPNERYLVGRVGPRDYRMYRCVVRYGYKDSTGNDDSEFENQLVFNLAEFIQTENSAPWIP

SSSEMSLDGRMTVMGTVTGSTASKASLSFPVSETRSERVTRIMQTFCGDSFQSFKTVSSPCATLDWQANFEIPLFVEHEN

EIDSEMRKELIDLIEAKEAGVAYLMGHSFVKAKKSSSWLKKFAINFVYTFLRRNCREPAMAFHIPHISLLEVGMVYYV

>PpHAK8

MVAVLMLAYQSFGVVYGDLCVSPLYVFRSTFSGDLRSHMTEGEISGVLSLIFWTLTLVPVIKYAIIILNADDNGEGGTFA

LYSLLCRHAKLSLILNRQNADSELLTHNLEQPPETPRGQTICRLLEKHVFLRNGLIIVVLLGSCMVIGNGILTPSIAVLS

ATSGISVAAPQLPQSVAVLVSCGILVLLFGLQHLGTHRISFMFAPIVLMWLFCNCAVGIYNLVTYNPLIIHALSPYYIYH

FFKVSGRDGWISLGGVLLCITGSEAMYADLGHFSKRSIKTAFTCIVYPCLVLGYMGQAAYLSKNLADVDHGFFHSIPGPV

FWPVFIVAMLVSIVGSQGAVTATFSIIKQCQSLGFVPWVKVVHTSRTIHGQIYIPEINWIMFVISLSITVGFQSPVEIGN

AYGISVICVMLATTLLMTFVIYVVWQHSIFTAGIFFLVFTLVEAVYLSSALFKVKQGGWVALVLAGVIMSIMYVWHYGTI

KNYEFDLQNKVSMKWLLTLGPGLGIVRVPGIGLIYTELLTGVPAIFSHFVTNLPAFHQILVFVCIKSAPVPFVPPDERYL

VGRVGCRDYRMYRYVVRSGYKDTYTNDESEFESQLIYNLAEFIRTESAAPWAQSRGEMPQDSRMTVMGALGSTRSVAETA

WSTDMPANAADSLESSDGITSPKRHVHFNLPKPSDNHVNAEVRKELADLADAKEAGIAYVMGHSYVRAKPSSSWLKKFAI

NFVYSFLRRNCREPAVALNIPHTSLIQVGMVYYV

>PpHAK9

MDTEVDSDERPPESRGARMHKSSLAAVLMLSYQSFGVVYGDLCVSPLYVFRSTFSEDPHSHITEAEIHGVLSLIFWTLTL

VAVIKYVIIVLSADDNGEGGTFALYSLLCRHAKLSLILNQQTADSELSTYKLEQPPETPRGEKVRKLLENNVFLKNGLLI

VVLLGTCMVIGDGILTSSIAVMSATSGITVAAPQLSENVAVLVSCCILVLLFGLQHLGTHRISFLFAPIVLLWLLCNCTI

GVYNLITYNPSIVRGLSPYYIYHFFKVSGKNGWISLGGVLLCITGSEAMYADLGHFSRNSIKVAFTCIIYPSLLLGYLGQ

AAYLSKNINDVDHGFYRTIPEPIFWPVFVTATLASIVGSQASITATFSIIKQCQALGFFPWVKVVHTSSTMHGQIYIPEV

NWIMFAISLSVTVGFQNTIAIGNAYGIAVIAVMLVTTFLTTLVILIVWQRSAFLAWGFFLLFGSVELIYLSAALYKVKQG

GWVSLVLAGSMMCIMYVWHYGTVKKYEYDLQNKVCMKWLLGLGPSLGIVRVPGIGLIYTELVTGVPAIFSHFVTNLPAFH

QVLVFVCIKSVPVPYVPAHERYLIGRVGSRDFRMYRCVVRSGYKDTYGSGDEDEFENELLYNLSEFIQTEGSAPWIASSN

EMSLDGRMTAMGALGASFAASNTGLSLPLSETQTERENTYNFNFNADSLESWEGVNSPPVVRKRHVHFNIAKSDTDMEAD

SEVRKELMDLIDAKEAGVAYVMGHPYVKAKPSSSWLKKFIIDCFYSFLRRNCRQPTTALHIPHMSLIEALLLLDCANITF

EHKMIMQGFSLLLTWRGKAPKLAKEEGRTDCSGLSREE

>PpHAK10

MDPESRIPQYTQKASLAAIITLAYQSFGVVYGDLSVSSLYVFRATFSDLQRADDLELYEVYGVVSFIFWTLTLIPVIKYS

FLVLNADDNGEGGTFALYALLCRHLKLSLILNQQAADEKLSLYKLEHEQTAESPRGVYFRRLLEKHKSLQTGLLIVVLLG

TCMVIGDGALTPALSVLSAIDGIRVAAPSLHKDVTVVLSCTILVLLFGLQHIGTRRVSFLFAPIILAWLFCNAGVGLYNL

IVWNPSIWRAISPYYIYYFFKTDGKEGWISLGGVLLCITGAEAMYADLGHFSRTSIKLAFTGVVYPSLLIGYIGQAAYLS

KHLDEYEHAFFRSVPAPAFWPVFIIATLASIVGSQAVISATFSIINQCMALGCFPRVKVVHTSNNIHGQIYIPEINWMLL

LLCLALTIGFQDVIDIGNAYGIAVITVMLVTTCLMTLVILIVWQLNIFLAFCFFAVFGSVELLYLSTAYYKVPQGGWVPL

LIAAVYMAIMYVWHYGTTKKYENDFQNKVSMRWLLELGPRLGIVRVPGIGLIYTDLVSGVPAIFSHFVANLPAFHEVLVF

VCMKSAPVPYVSPHERYLVGRIGPKDYHMYRCVVRYGYKEVRGDENDFETQLVANLAEFIQTEEAISSNEESFEGHMTVM

GTTLGLLLNPPRKDDIQLPRMSEESCTSIVMSNSGDSLQPTDWLTTPPGVILKRRVRFDIPMSESTDDVDSEVCKELAVL

STAKDAGIAYMMSHSYVKAKKSSSLLKRFTINYAYTFLRKNSRDPAIVFNIPHASLIEVGMFYYV

>PpHAK11

MQYTERASIAVLLTLAYQSFGVVYGDLSVSPLYVFRATFGDTLRNDVEEREIMGVLCFIFWTLTLIPVIKYSFIVLSAHD

NGEGGTFALYALLCRHLKLSLILNQQAADEELSSYKLEQPTTSPRGVWFRHLLEKHKFLQNGLLIVVLLGTCMVIGDGAL

TPALSVLSAISGIRVAAPHLHENVTVAVACCILVLLFGLQHMGTRRVSRLFAPIILAWLLCNASIGMYNLITWNPSILKA

LSPYYMYYFFKMDGKEGWIALGGVLLCITGAEAMYADLGHFSRKSVKLAFVGVVYPSLLIGYIGQAAYLSKHLNEVDHAF

FKSVPRPVFWPVFVVATLASIVGSQAVISATFSIINQCMALGCFPRVKVVHTSNQVYGQVYIPEINWIMFILCLTLTISF

QNTIDIGNAYGIAVIIVMLVTTFLMTLVIITVWQCSIFWALCFFAVFGCIELLYLSTAFFKVPKGGWVPLVLAGVFMSIM

YVWHYGTTKKYEYDFQNKVSMKWLLNLGPSLGIVRVPGIGLIYTDLVSGVPAIFSHFVTNLPAFHEVLVFVCMKSAPVPY

VSQHERYLIGRIGPKNYHMYRCIVRYGYKDVRRDEDDFENQLIANLAEFIQREEATSSNEHSFEGDRHLAVMGTTPGLLF

NSLSDRREESIKSSVISNNGDSLQLQEWLSSSPRPIHKRRVHFDIPMSEAHHSTDVRKELSVLAKAREAGLAYMMSHSYV

KAKKSSNFLKKCAIDYMYTFLRKNSRDPAVVLNIPHTSLIEVGMFYYV

>PpHAK12

MSATDSETVRSSAFKPQNASLKVILLLAYQSFGVIYGDLSTSPLYVYRSTFAGKLRMHENDDEILGVLSFIIYTLTIIPV

IKYVFIVLAADDNGEGGTFALYSLLCRHAKLSLLPNQQAADEELSTYKLEAPQESNRDIWMKKILEKHQKLRTVLLIVVL

LGTCMVIGDGVLTPAISVLSAVSGIQVAAPDLHDHVIILVSCIILVGLFALQHYGTHRVAFIFAPVVIAWLFCISSIGVY

NVVTYNPHIWRALSPYYMYNFFKKCGKDGWVSLGGIVLCITGTEAMFADLGHFTPLSIKIAFGGVVYPCLLLAYMGQAAF

LSKHRDDISRSFYKSIPKPVFWPVFCVATLAAIVGSQAVISATFSVVKQCLSLGFFPRVKVVHTSKQIYGQVYIPEINWM

LLVLCLAVTVGFRDTITIGNAYGLAVMTVMLVTTCLMTLVILIVWRRSIVLAACFLLFFGSIEAVYISAMYIKVPEGGWV

PLLLSLVFMSIMLVWHYGTTKKYEFDLQNKVSMKWILTLGPSLGIVRVPGIGLIYTELVTGVPAIFSHFVTNLPAFHQVL

VFVCIKSVPVPYVPQHERHLIGRIGPKGYRMYRCVVRYGYKDVHKDDNDFENQLIVNLAEFIRTEAEVTYLPSSSEVTAE

VVADERMTVMGNTPSSRILNVFGTGSDFEQSSVSVPTRVSPLGSFQITEENEAIRAGCKKRVRFEISKSPDLDPAVRQEL

QELIEAKEAGVAYVLGHSYVKAKKSSSIIKRFAIDVAYTFLRRNCRRSAVALSIPHISLVEVGMIYYV

>PpHAK13

MAETNTGLQSDWCFVHRPIPILILSRKLGSSQQRSKRSSSGGGGGSRAVDAPNYDLEMGAFREGERDWKFVKNDGETAFD

LKVNPLNENEAREETSVVERIPSKVGLNSKQFRLRRAVSDVRFKAKLAPVHPDKKSYSKWETIVLAYQTLGVVFGGLGTS

PLYVWPTIQISNPGETEFLGVLSLIFWTLTLVALIKYGLIVINADDHGEGGTFAVYSILCRNANFGQNIADPSVYILAGA

NMNKETRAKESESTLAKKLRHFIERHERAKFVLFVMVMLGTGLVIGDGILTPAISVLSAMAGIQSEDPSINTSVVTWTSA

AILVALFLVQRFGTNRMGFLLSPIMLIWFLVTPVIGVYNVVQYYPSIFKAISPRYIIEFFRVNQKEGWIALGGVVLCITG

AEASYADLGHFNKRSIQIAFYCLVYPSAILTYAGENAYLIAHPGDHKNAFFKSVPKAVYWPVFIVATLAAIVASQSLITG

TFSLIKQCTSLGCFPRVKMVHTSADQEGQVYSPEINWMLMVLCIAVVVGFQDAGTLGNAFGVAVVGVMLITTILVTLVMI

LVWKLPWPVALLFLTVFGFIEGVYFTAVCVKVPHGGWVPFAIASMFLAISFCWNYGRRTKHSYEVSHKTSLDNLGASIFS

MGTQRVPGICFFYSDLALGVPPIITHYMKNVRTLHQVLVFTTIKFLPVRTVAPEDRFYVGRVGFKGVYRCVACYGYQDVI

DCKDGAFKDHALRSLQLYLENEERNEPDANGMDGRTPSFQRTIAAHNLEDLMELNKAREVDAVHVVGKITVRTTESTGWF

GRLVINKGYSILRILCRSVVKELQIPPANYLEVGMLYEI

>PpHAK14

MIGILCELAVEKHSASSSSLKEQSAKIVNYRLSSVLTLNSSYIYGWLGRGELCEIRDSFFELFSQSREKGTDGKVKPNYH

LELTHAYIGANGKARKDIEQSGLGILERLNLTTEGSKALIKKSCEGLLEQSLHLIEQNMNPIITEKIEAEMLKGTLISLT

GNCQTGHRVAMPHVLKVAHQMINRCEVLECNSTKMALSPEVTETGSVESSSPSSITIASRAHKDFDRVSHPKKSQYATEY

FLHSPVGERFFQAPIDVPRKTHTKWQTLAFAFRTLGIVYGDLGTSPLYVYPSIISAETPNEDDYLGILSCIFWTLTLIGV

IKYTLIVLWADDHGEGGTFAMYSLICQHTDVGGQTRKINKEHSKVREWLERNKCAQEVLLFVVMLGTCMLIGDGILTPAI

SVLSAIGGIRAEVPSISQNVVVWVSAVVLVVLFCCQSYGTNKVAFLFSPIMATWLLTTPMVGMYNIIIHYPTVFKAFSPA

FIYRFFQKNGKQGWHMLGGIVLCITGCEAMFADLGHFNRLSIQLAFSFLVYPSVLLTYAGQTAYLINHPENYLEGFFKMV

PEPVFWPMFVVATLAAIVASQGLITATFSVIKQSVALDYFPPVKIVHTSEYSEGQVYSPEVNYGLMVLCLAVVFGFRSAT

TIGNAFGVAVVCVMIITTCLMAIVMLVIWNTNWLFVSVFFTVFIFVEGCYFSVVITKIPQGGWLPFIISILFTLIMTSWN

YGRRKKFEYEMKNKLSKKTLGELLSRIGDHRVPGVCFFYTDIFHGIPPIVKHYVQNVRTLHQVIIFTTVRHIPIKTVLAA

ERFLVGRIGFTGVYHCVARYGYMDLLSSETTYFLDQVTQCLTKHIGSALDFSDNPDEVGQEDERAREIQMIVNAKAAGAV

YVLGRSEFKVDNNTSYLDRIVAGIFYPFLNSICRSPVSSLHIPPANFLELGMYYDLL

>PpHAK15

MDLECVSFKESSFRRVQFMSKASMVALLTLAYQSFGVVYGDLSVSPLYVFRATFGSRRRGDVEEREIMGVLCFIFWTLTL

VPVVKYSFIVFCAHDNGEGGTFALYALLCRHLKLSLILNQHAADEELSTYQLEQPITSSKGIWFRQLLDRHKFLRNGLLI

VVLLGTCMVIGDGALTPALSVLSAISGIRVAAPHLHENVTVAVACCILVLLFGLQHIGTRRVSCLFAPIILAWLFCNASI

GLYNLITWNPSILKALSPYYMYHFFKVDGKEGWIALGGILLCITGAEAMYADLGHFSPKSVKLTFVGVVYPSLLIGYVGQ

AAYLSKHLDQVDHAFFKSVPNPVFWPVFVIATLASIVGSQGVISATFSIINQCMALGCFPRVKVVHTSNHIYGQIYIPEI

NWIMLILCLGLTIGFQNTVGIGNAYGIAVITVMLVTTCLMTLVIITVWQRSIFLALCFFGLFGSIELLYLSTAFFKVPKG

GWVPLVLAGILMLIMYVWHYGTTKKYEFDFQNKVSMKWLLNLGPSLGIVRVPGIGLIYTDLVSGVPAIFSQFVTNLPAFH

EVLVFVCMKSAPVPYVSQHERYLVGRIGPKDYRMYRCVVRYGYRDVRRDEDDFENQLIANLVEFIRTEEAMSSNAQSFEG

DQHLTVMGTTPALLSNGHSVKEMETDKSVAISNNESLQSMEWISPPTSLIPTRRVHFDIPVSETVDSDDVRKELSALAKA

KEAGVAYVMSHSYVKAKMSSNFLKKFAMDYMYTFLRKNSRDPAMILNIPHTSLIEVGMFYHV

>PpHAK16

MVTVPRSDMATTDAETGSSNYGAQEKLPMKALLILAYQSFGVVYGDLTTSPLYVYRNTFSSFGLKIHESGEEILGVLSFI

FWTLTIIPFIKYVFIVLCASDNGEGGTFALYSLLCRHANLSLLPYQQDADMQLSTYKVETSREVKQGLRAKEFFEKHPRF

RTGLLVVVLLGTCMFIADGVFTPAISVLSAVTGIKVAIPSLHEDIVIAVSCCILIGLFALQHFGTHQVAFLFAPIVIAWL

FCIASVGLYNIIIYNPRGIWAALSPVYMYKFLKLAGRDGWTSLGGIVLCMTGTEAMFADLGHFNQMSIKIAFTTVVYPCL

LLGYIGQAAYLYKNPGDVSESFYKSIPRPVFWPVFVVATLAAVVGSQAVITATFSIIKQCQSLGCFPRVKLIYTSKRIHG

QIYIPEINWILFILCLAVTVGFRDTITIGNAYGLAVITVMLVTTCLMALVILVVWRRNIIEALGFLVIFGVIELFYISAC

IMKVPQGGWVPLVLTVVFMSIMYIWNYGTIKKYEYDLQNKVNMETLLKIGGNLGLVRVPGVGLVYTKLVTAVPPIFSHFF

TNLPALHDVLVLVSIKSVQVPYIPSNERCLVGRIGPKRLRMYRCVVRYGYKDIHKDDHKFEDKLLQSLGEYILMEDDAEE

EGNGFDDGADGKMHLPGIQSSSLVHAVNNDGASKERFGKGKESSGNSKVSSSENPSRTNGKKRVRFETPARKELNPAVRQ

EYEKLKEAREKGVVYILGHSHVQASSASSMIKKFSINIVYTFLRRICRGPGVVLHIPQENSIQIGVVYRV

>PpHAK17

MVGIVIMQDNVPIKAILFLAYQSFGVVYGDLSTSPLYVYRSTFSGRLGLYESDDEIIGVLSFIFYTLTIIPLLKYVFIVL

NASDNGEGGTFALYSLLCRHAKLSLLPNQQDDDQNLSTYKVETPEQTKVGLRVKNLFEKHPHLRKGLLIVVLLGTCMVIA

DGVFTPAISVLSAVTGIRVAAPDLPESVVTAVSCGILLGLFVLQHFGTRRVAFLFAPIVIAWLLCISIIGVYNIIVYNPR

GIWAALSPVSMYKFLKSAGKDGWISLGGVVLCITGTEAMFADLGHFNELSIKIAFTTVVYPALILGYFGQAAYLSKNRND

VSESFYKSIPTPVFWPVFVIATLAAIVGSQAVISATFSIVKQCVSLCCFPRVKVIHTSKEIHGQIYIPEVNWILFLLCLS

ITVGFRDTTTIGNAYGLAVMTVMLVTTCLMALVILIVWGRSIILALAFLIFFGSIEAMYISVTILKVPQGGWVPLVISFV

FVIIMYIWNYGTSKKYQYDLENKVAMQTLMQIGPPMGGVRVPGIGLYYTELVTGVPPILAHFFTNLPALHEFLVLVTIKH

VPVPYIPSQERYLVGRIGSKDLRLYRCIVRYGYKDTHKDDNTFEDKLIKKLGEFILAEDDAGSESFSSDDRAGGVMQSSG

ISRSLCLVHAVNNESVIMERLEERDRPLEMSQIACREPPSRRGGKKRVRFESPERQQPHPAVVREFEMLREHKERGVVYI

LGHSYVEATGASSLVKKFAINFVYTFLRRICRGPSVVLHIPQASSIEIGVVYRV

>PpHAK18

MAAVDAEYGRLFLHGNQENIPLKAIILLAYQSFGVVYGDLSTSPLYVYRSTFAGRLRLHESDDEILGILSFIFYTLTIIP

LIKYVFIVLNASDNGEGGTFALYSLLCRHGKLSLLSNQQDDDQNLSTYKVVTPKQTQLGLRVMNLFEKHPHLRKGLLIVV

LLGTCMVIADGVFTPAISVLSAVTGIKVAAPDLPEGVVTAVSCGILFCLFVLQHFGTRRVAFLFAPIVIAWLICISIIGV

YNIVVHNPRGIWSALSPIYMYKFLKITGKDGWVSLGGVVLCITGTEAMFADLGHFNQVSIKIAFTTAVYPALLLGYFGQA

AYLSKNRNDVSESFYKSIPTPVFWPVFLIATLAAIVGSQAVISATFSIVKQCVSLYCFPRVKVIHTSREIHGQIYIPEIN

WILFLLCLAITVGFRDTTTIGNAYGLAVMSVMLVTTCLMALVILLVWGRSIFIALGFLIFFGSIELMYISASIMKVPQGG

WVPLVISFVFLIIMYIWNYGTSKKYQYDFENKVAMHTLMNIGQTMGGVRVPGIGLYYTELVTGVPPILAHLFTNLPALHE

FLVLVSIKHVPVPYIPLQERYLVGRIGSKDLRLYRCVVRYGYKDIHKDDDGFEDKLIEKLGAFIVAEDDVESETCSSDER

DDGMMQASGMYRSSSLVHAVDNELTVKERFGRKKQPLAKSELSCNEPTPQVNGKKRVRFQSPDFKQPDPAILRELELLRE

HKERGVVYILGHSYVEATNASSILKKFAINVVYTFLRRICRGPSVILHIPQASSIEIGVVYRV

>SmHAK1

MHIHSLYLLEFWQWKTITLAYQTLGVVYGDLGTSPLYVYPTMNFKSPQGEDFLGTFSIIFWTLTLIGLAKYVFIVLHADD

HGEGGTFALYSLLCQHLKTTIKGHKYGRMLSDSKLSHFSKTNEAESPTAFARFIERRKSAQRLLLFVAMLGTCMLIGDGI

LTPAVSVLSAMAGIATATPKINQSTVVWLSAAVLVALFLFQRFGTKCVSFVFSPIMAIWLVTTPLIGLYNIAIHYPQIYK

ALSPHYIVTFFQRNGKDGWLALGGTVLCITGAEAMFADLGHFTKSSIQVAFAFMIYPSVLLTYAGQSAYLVRHPGDHREA

FYKFLPRSIYWPMFVVSTLAAIVASQGLISAAFSIIKQSIALDYFPPVTMVHTSQDKEGQVYSPQVNYCLMVLCLAVLFG

FQGGPEIGNAFGVAVIWVMLITSSLITLVMLIIWRTPVILALVYITVYGTLEGVYLSSVLTKLREGGWFPFGIAVILALA

MFSWHHGREKISDYEMINKVTVDSVDELFSRAAKQRVPGLCLFYTDLVHGVPPIMRHYVNNVRSLHQVIIFTTIRYVPVR

TVLPDERFILERCGYAGVYRCVAQYGYMDVLEGEEFVSDVIEALAIFVASREDHEELDSDGDGAALVSDLWRAKAAGAVH

VMAKADFRMSEERSGWFERLVLDGIYRFLRNNCQMPVAALKIAPQNVIEIGMMYEI

>SmHAK2

MAEAEDSPGKGYMWNLDRIDQPFGEEADRVHNLHTAKKLSTTTTLRLAFLSLGVVYGDLGTSPLYVFSNIFPDGIKDRND

LLGTLSLIIYTITLIALVKYVFFALRANDNGEGGTFALYSLICRHAKVNTIPNQHHTDRALTTYSFRPMSKKSTAYKLKN

ALETSLFLQKILLVLVLLGTSMVIGDGMLSPSISVLSAVQGIRLSHLELPKGCVLILSLLILVALFSMQRFGTAKVGFMF

APIIFIWFISIGTIGIYNIFVHYPPVFKALSPVYIFRYFRAQGVTAWISLGGVVLSVTGAEALFADLGHFTAQSIQLAFT

IIVFPCLIAAYMGQAAYLMKYPRDVDEPFYNSIPNRPFIYWPMFVVATAAAIIASQATISATFSIVKQAVALGCFPRVKI

VHTSHRFLGQVYVPEVNWTLMVACLLITAGFRETQQIGNAYGVAVVLVMVVTTFLLAMVMILIWHSNLYLAFSFLAVFGS

LELLYFSSVLFKVTSGGWVPLAIGSVLMAVMYFWHYGSCERHKFELQNKVSLGWILQLGPSLGMVRLPGIGLFYTELAHG

VPSIFSHFLTHFPAVHSILTFVCVKYLPVSTVAKEERFLLRRIGPKQFRMYRCVVRYGYKDLHKKDDHFDELLIRALAAF

IRYESLMESVDEQSEETVTSNGSLESCGAAPPLQAQVDGHTITGSEICLTASSVSSIQRQTPRSLREEEDECAFLIKCKE

DGIVHIMGSTVMRARQGSGFFKRQAINSGYSFLRKLCRDTSVIYHVPHESLLHVGMVYNI

>SmHAK3

MWKLDQTLDQPLGEEAGRGLSTATTLWLAFLSLGVVYGDLGTSPLYVFSSIFQDTSTVENTDDILGTLSLIIYTITLIPL

IKYVFIALQSSDYGEGGTFALYSLICRHVKANTIQNQHHTDLKLTTYSRRPVAPNSLAFKVRTLLENNSHLQKILLVLVL

LGTSMVIGDGILSPAISVLSSVKGIQAAHHSLPEEAVLVLSLLILVILFCMQRFGTGKVAFIFAPIIFLWFLSIGLIGLY

NIFRHDPSIFRALSPLTFIRYFHRSEVNGWVALGGIMLSITGAEALFADLGHFSALSIQLAFTFLVFPCLLAAYMGQAAF

LMHHPDRVADAFYSSVPGPLYWPMFVIATAAAIIASQATISATFSIVKQAVALGCFPRVKIVHTSQRFMGQIYVPEVNWI

LMALCLLITAGFRKTTQIGNAYGVAVIGVMLVTTLLMALLMVVIWQTNIFLVLLFLAVFGTVELVYISAVLFKVPNGGWV

PLAIGAVLLLVMYTWHYGSTQRYETEKRNKVSVGWILGLGPSLGLVRLPGIGLFYTELAHGVPSIFSHFLTHFPAIHSIL

VFVCVKYLPVSTVPKEERFLVRRIGPKNYRMFRCAVRYGYKDLHKRDDHFDDLLIQTLAAFVKYESLLESVDGQDDGNSE

LSQVVYAASSSQSQQEQQEHDHQETRNTVAGFMSNSLTSVNSSTQLTSYSSDGDDRDDRRQDELKFLHSAKEQGIVHILG

NTLIRCNEGSGLLRRATINYCYGFLRRICRDNSVIYHIPHESLLHVGMVYNV

>SmHAK4

MDGLFVCSWQSYRRKSLKAVLLLAYQSFGVVYGDLSTSPLYVYRSTFSGKLQLHEDDTEVLGVLSFILYTLTLIPLLKYV

LIVLRADDNGEGGTFALYSLLCRHAKLSLLPNQQAVDEELSTYKLQNVRESYRGARMKGLLERHKSLRIALLLVVLLGTC

MVIGDGVLTPAISVLSSVYGIKVAVDDLNKHVVELIACLILVGLFALQHHGTHKVAFMFAPIVLAWLFSIGAIGIYNIAR

WNPHVVRALSPYYMYKYFKRTGFDGWISMGGVLLCITGTEAMFADLGHFSELSIQIAFGCVVYPCLVCAYMGQAAYLSRN

HSDIEGSFYKSIPKPVYWPVVVIATLASVVGSQAVISATFSIIKQCMSLGCFPRVKVVHTSKDIYGQIYIPEVNWILLIL

CLAVTLGFRSTIFIGHAYGLAVITVMFVTTFLMSLVIVIVWKRSIILAIIFFMFFGTIELMYVSSAMLKVHEGGWVPLAL

SVFFVAVMYTWHYGTAKKYDFDLQNKVSMKWLLTLGPSLGIVRVPGIGLIYSELVTGVPAIFSHFVTNLPAFHQVLIFVC

IKSVPVPYVRPEERYLIGRIGPKEYRMFRCIVRYGYKDVHKDDNDFENQLIFNVGEFIQTEASSTWAPSSSDHSSVDGRM

TMMGLPLQSSIKMVTSGLEDSDKQSIRSLSLGTPEIEALQPRRVRFELPRSPELDPDIRAELTELFDAKNSGVAYMLGHS

YVKAKRSSSFMKKFVIDVCYNFLRKNCRGPAVALDIPHICLIEVGMIYYV

>SmHAK5

MVGSGSPEIQSAGISSSDDDEDSTAGVDYLRRRLKRLISRHDSLEEEAAYFPWMHSHNQSSSGLLLFKLAFQSIGVVYGD

LGTSPLYVFSSTFTGGHIPNPEKDIVGALSLILYTLLLIPLCKYVLVVLRANDNGEGGTFALYSLISRYAKISVVHPTDR

QLSTYKLQVPSKELERALWIKEKLENSGLLKNLLLLITLIGTCMIIGDGTLTPAISVLSAISGLKVAVPAMDQNVVVIVS

IVVLVILFSLQRFGTSKVAFLFAPALLLWFLTIGVIGLYNLSRGDMRVFQALNPWHIYLYFKRNGKVAWISLGGIVLCIT

GTEAMFADLGHFSVKSIQIAFTTVVLPCLLLAYGGQASYLIRNPEHVGEAFYKSIPGPIFWPVFVIATMAAVIASQAMIS

ASFSVMKMAESMGCFPRVHILHTSKRFPGQIYIPEINWLIMILTVALTAGFKDTTQLGNAYGIAVVATMCVTTSLVTLIM

LMIWQINVLVALGFFLLFGTIELAYLSSVLFKVTEGGWVPLVLAAGLLFVMYIWHYGTKMKHKYEVRHKLPMDWISQLGS

NLGTVRVAGLGLVYNELVHGVPGIFHRFITYLPAIHSVLVFVCIRYVPVATVPREERIVVRRIGPKSYHMYRCIVRYGYR

DMRTETAWLFEQLLVECLENFIRREAREEALERAENAAAAANNESLCTPLLLRRVESGEFEEDLMVADNDDEAGSSVSED

DSLALLRKCRETGIVYLLGHGDVRARKDSFFLKKLVINYFYAFLRRNCKQRAETLNIPPGQLLRIGMTYFV

>SmHAK6

MWSLDQIDQPLGDDAARVQSMQAPPKRVSTAATLRLAFLSLGVVYGDIGTSPLYVFSNIFPDGIQHREDVLGALSLIVYT

ITLIALVKYVFIALRSGDNGEGGTFALYSLICRHVKVNTISNQHPTDRELTTYSFRAVPEKSHAHKVKVALEKSQTLQKI

LLVLVLLGTSMVIGDGMLSPAISVLSSVRGLRVAHLSVSDDAILVLALVILVGLFCMQRIGTARVGFMFAPIIFVWFLAI

GALGVYNIVVHDPSIFKALNPHYIIRYFGRQKTRGWESLGGVFLAITGAEALFADLGHFSASSIQLAFTGMVFPCLLAAY

MGQAAYLMKLPDDVNDAFYKSIPKTPAVYWPVFVIATASAVIASQATISATFSIIKQAVALGCFPRVKIVHTSYKYLGQV

YIPEVNWLLMVACLVITAGFRETMQIANAYGIAVVGVMLVTTLLMALVMLIIWQRNLLLVLAFLVVFGSLESTYISAVLV

KVEKGGWVPLAIGAFLLIVMYTWHYGTTERHSFELQNKVSLGWILRLGPGLGMVRLPGIGLFYTELAHGVPSIFSHFLTH

FPAIHSILMFVCVKYLPVSTVPKAERFHIRRIGPREFRMYRCAVRYGYKDLHKKDDEFDELLFQALRSFVRYESMVGSVE

NSDDSIESSRVISAEPTRSNIDSEDEGDFLGRARQDGIVHIMGNTVMRAREASSFWKRVAINFGYSFLRRICRGSSVVYH

IPHESLLHVGVVYDV

>SmHAK7

MEEHQDGKGTMWELDQTLDQPLGDEATQVRGFQNSKEHPLAVTIRLAFISLGVVYGDLATSPLYVFPSVFPDGIVDRRDV

LGAVCLIVYSFTLIPLIKYVFIVLRANDNGEGKFRSIVLVLGGTFALYSLICRHAKVNTIPNQHPTDQYLTTYSRRPVPE

NSRASTIKKLLEGRNSLQKLLLVLVLLGTSMVIGDGVLTPAISVLSSVSGIKVAHPSFHQGHVVILALIILVLLFSMQHV

GTDKVGVMFGPVILVWLLSIGAVGVYNIAIHKPDIFRALSPVAGFDFLRRTKSKGWARLGGIVLSITGAEAMFADLGHFS

TVSIRLAFTSLVFPCLLAAYLGQASFLLKFPDKVDQTFYRSIPDPVYWPMFVIATVAAIVASQATISATFSIVKQSVALG

CFPRVKIIHTSNRILGQIYVPEVNWILMLLCLAITAGFRETTQIGNAYGIAVMAVMLVTTLLMTLIMLFIWQTNLSLVLL

FLVTFGSVETIYFSAVLFKIAKGGWVPLAIAAALMLIFYAWHYGTVKRYQFEIQNKVPLAWILGLGPSLGLVRVPGVGFV

YTDLAHGVPSMFSHFITHLPAIHSVLVFVCVKYLPVNTVLEDERFLFRRIGPPDYWMYRCTVRYGYRDLHRRDEQFEERL

IGALADFIRKDDDNNRVVLSQRRIEHQVVEDQLKFLVAAKESGVVHILGNTVVKARKGSGLAKRIAINHVYSFLRKVCRE

TSVIYHIPHETMLNVGMIYDV

>SmHAK8

MEESNPTGDEAPPSRSGDRTLSRYDSLERIASKVSGLANVKVMTTALLLRLAFQSIGVVYGDLGTSPLYVFSSTFPNGID

PQHVEANVLGVLSLIIYTLTLSPLLKYVLVVLQASDNNEGGTFAVYTLLCRSINVGVFGRKAHPDDRALSGYDVVPRITG

RFREGIRNFMEGRKAVHMILLLVTLLGTCMVIGDGTLTPAISVISAVQGIQVQVSSLGQNIIVAISVVILVLLFNLQRFG

TDKVGFMFAPVLTVWFVAIGVIGLYNIGAHDLSVLRAFNPKFILDYFLLRKLDGFISLGGVVLCITGTEAMFADVGHFSA

RSIQIAFVPFVYPTLLLAYCGQAAYLMKHPEDVANAFYKSVPAAVYWPMFVVAVLSAIIASQAMISAVFQIIKQSQAMSC

FPRVKVVHTSKRFPGQVYIPEMNWFLMLACVVITIIFKNTTTIGNAYGICVVSVMSVTTFLTAIIMLLVWKTNILLILAY

FAIYAMLELTYFSSVLVKFTEGGWLPMLFAAIFMSIMFTWFFGSSRRNKYELENKMSVEWITGLVTNNSILRVRGVGLIY

TRLSQGVPAMLSHYVSNVPAIHSVLVFVTIKNLPVSSVVSEERFLFKRVGSKELRIYRCIARYGYRDHHRGDNEFENSLF

QSLERFIRLDEAPSSTPAAAEANHREESDGTTRIEVFPVVGGDLNGGGSAVEQEIISSPQEEDEEEIEFLRNSRRAGVVY

VLGHTEVVARKDSSVMTKFFINTLYAILRKNFRESRLILEVPHERLLKIGNVAYI

>SmHAK9

MEGTDMEESHGPPPRISSSKKLGRMDSLERDAGKVTGMENHGRKILTRAVILRLAFQTIGVVYGDIGTSPLYVFSSTFPG

GISRDHLKTNVLGVLSLIIYTLTLSPLIKYVFVVLRANDNGEGGAFALYSLICRNANVDVMGKRHPEDKNLSAYKLDLPN

QGRIRRGIWIKNFLEGHKAVHVVLLMITFFGTCMVIGDGTLTPSISVLSAVQGIQVQVPNLSQSVIVVVSIVILICLFSV

QRFGTDKVGFMFAPVLTIWFAMIAMIGLYNLIHHDHGVLAAFNPKYIFDYFKTNKREGFISLGGVVLCITGTEAMFADLG

HFSVPSIQIAFTTYVYPSLLLAYIGQAAYLMEHPEDVGRAFYKSVPKPLYWPMFVVAVLAAIIASQAMISAVFQIIKQAE

ALGCFPRIKVVHTSKNFVGQVYIPEMNWFLMCACVLITAAFRDTTTIGNAYGICVVMDMAVTTTFTTIIMVLIWKTQLFL

ALLYLLVYWSVEFTYFSAVVYKFKDGGWLPLLFAALFLTVMVIWFSGNSKRYKYELDNKISMDWITGLGSNLGVSRVRGV

GLVYTELAQGIPSIFSHYITNLPAMHSVIMFVTIKNLPVSNVLSEERFLFRRVGSKEFRMYRCIARYGYKDCHRGDTQFE

EDLFKSLAEFISIEDDGKQMEARHLGEADTDSCSVAIYPVSLQLSPPQAPEESAIAIPGSGVVEELGFLEESRKAGVVYL

LGDNDVRAREDSSFINKFVVDYGYAFLRKNFRESTLILNIPHTRLLKVGMVYFI

>SmHAK10

MSTAGLDPESATSAPGASAGGTTLQTNYKQAVKGNSLRALLCLTYQSFGVVYGDLSVSPLYVYRSTFSGKLRLNENDEEI

LGVLSFIIYTLTLLPFIKYVLIVMNADDNGEGGTFALYSLLCRHAKLSLLPNQQPADEDLSTYKLEGGRTNRKSGGVPFK

AFLERHRHLRISLLAIVLLGTCMVIGDGVLTPPISGPAFFFPLFPESTLRKPSTMNHFGTHRVAFIFAPIVIAWLFCIAA

IGVYNIAAWNPGIFRALSPYYMYNFLRKTGVEGWTSLGGILLCITGTEAMFADLGHFSKLSIKIAFTCVVYPCLVLAYMG

QAAYLSKNHDDILKSFYKSIPKTVYWPVFVIATLASIVGSQAVISATFSIIKQCLSLGCFPRVKVVHTSKDIYGQIYIPE

VNWMLLLLCLAVTLGFRNTILIGHAYGLAVVTVMFVTTFLMSLVIVMVWRKSIFLAAAFLLFFGSIEAFYISAALIKVRE

GGWVPLVLAVIFMAVMYIWHYGTSKKYEFDLQNKVSMKWLLTLGPSLGIVRVPGIGLIYTELVTGVPAIFSHFVTNLPAF

HQVLVFVCIKSVPVPHVPPQERYLIGRIGPKEYRMYRCILRYGYKDLHQDDQDFENMLIVNIGEFIQMEDAHPWIPSSTE

VSVDGRMTVVGTPSRAAMRLVTSGLEDVEPPPQQSVSFRMDRPSGKELLEEQELEEAELPRLDNKKRVRFELPKAAVEMD

PSVKAELLELIEAKEAGVAYVLGHSYVKAKKASSFVKKFAIDVVYNFLRKNCRNSTVALSIPHICLIEVGMTYYV

>PtHAK1

MGVVGLCMLQLCDIWKWKKMELGAQIASKEAEDVQIEVMVNGVENEEGDVLKGNKLHGNFQCSKFKRVDSLDLESSTVSG

MATHGSNLVTVASILQLAFQSIGVVYGDIGTSPLYVFASTFTDGIRHPDDVLGALSLIIYSLTLLPLLKYVFIVLWANDN

GDGGTFALYSLICRHAKVSVVPNQQAEDRELSSYKLAVPSRELKRALKIKEALEKSNAAKTILLILALLGTSMVIGDGVL

TPCISGIIPPPNPISPILGKILTIKTIILLIGFVLSAVQGIKQVDKSISQEAVAMISVAILVMLFSVQRFGTRKVGYSFA

PAILIWYSSIAFIGIYNIVKYDTTVFRAFYPKYIFEYFRRDSQRAWISLGGIVLCVTGTEAMFADLGHFSVRSIQIAFTG

IVYPALICAYVGQAAYLKKFPENVSDTFYKSTPSAIYWPMFAVAIVASIIASQAMISATFSIIKQSMSLGCFPRVTVVHT

SSKHEGQVYIPEINYILMLACVIVTISFQDTTKIGNAYGIAVVGVMIVTSSFLTLVMLMIWQTHLLLIVIFVTVFGAIEF

TYFSAVLYKFPQGGYLPTAFAAVLLFVMYVWHYVHVKRYAYEVEHKVSTEHMLSLGSSLDVTTVPGIGLLYTELAQGVPA

IFEHFMRNLPARHSVLVFVCVKYLPVNTIPAEERFVFRRIGPKGHKIYRCVARYGYRDTRTGNVEFENQLLESLREFIQF

DYICRNDMNKQSQHEGNSSNSGESIVNIDAASETEDMKACSMVSVKEELEFLENAKESGVVYLLGDSEVIANKDSSLFKR

FIVNFAYNILRRNCRKGQAALEIPHKNLLQVGMTYYI

>PtHAK2

MDTLQVLTTAATLQLAYQSIGVVYGDIGTSPLYVYSSTFSDGVIHHPDDIIGAFSLIIYTLTLLPLCKYVLTVLWANDNG

DGGTFALYSVICRHAKVSLIPNHQAEDRELSTYKLAAPSKQLKRASKIKEALENSNVAKIILLLLTLMGTSMVMGDAILT

PSISVISAVQGIQTIKESISQDVVVGISVAILVTLFSVQRFGTDKVGSVFAPAILIWFLFILFIGLYNLAMHDTSVLRAF

YPKYIIDYFSRNHKRAWNSLGGIVLCITGNEAMFADLGHFSVRSIQISFTLLVYPSLICAYGGQAAYLSRFPNHVSSAFY

KSIPGPLYWPMFVIAVIAAIIASQAMISATFSILKQAMYLGCFPSVKVVHTSAKYAGQVYIPEINYLLMTACVLVTVSFK

NTIMLGNAYGIAVVGDMIVTSTLLTLIMLMVWQTPIYFVAMYVLLIGTTEWIYYSAALYKFPKGGYLPLAFAAALLFTMY

VWHYVYVRRYAYEMEHKVSKEQIDSLFSSLSVARVPGIGLLFSELAHGIPLIFSHFITNLPAVHSVVVFVCVKYLPVNRV

PAEERFRFRRVDPKEYKMYRCVAMYGYTDVRIGNVEFESDLLKSLKEFVQSEYMYRLNEIEIRESQTFELTAKSVGSEDE

GEVDAMVSSQSRRFVEVAEFESQQDIEFLQEARKAGVVYLVGDSQVNAKTGSRLLKRIVVNTVYRFMRRNCRQERTSLDI

PHKSVLRVGMDYEI

>PtHAK3

MTKLQDPSLGHTLGLAFQTLGVVYGDLGTSPLYVFTSVFSTAPIKGDEDVLGALSLVMYTIALIPLAKYVFIVLKANDNG

EGGTFSLYSLICRYAKVNLLPNQQPSDEKISSFKLKLPTPELERSLRIKESLERSTKLKTLLLLLVLMGTSMVIGDGILT

PAISVMSAVSGLQDEISSFTEDTVVIVSIVFLVILFSIQQFGTSKVGMTFAPALLIWFFSLGSIGLYNIISHDVTVLRAF

NPVYIYYFFKRNTIKAWMSLGGCVLCITGAEAMFADLGHFSVRSIQIAFTFVVFPCLILAYMGQAAYLMKHPAVVEKVFY

ASIPDGLFWPVFVIATVAAMIASQAMISATFSCIKMSMALGCFPRLKIIHTSRRFMGQIYIPVMNWFLMIMCVLVCACFR

STTEISNAYGIAEVGVMMVTTSLVTLVMVLIWQKNIFLAIGFPLLFGSIELTYFSAVLMKVGEGGWLPLVFATCFLLVMY

IWNYGSVLKYQSEVRKKVSMDLMLELGSNLGTIRVPGIGLVYSELVRGVPAVFGQFLTSLPAIHSIIVFVCIKYVPVPVV

PQDERFLFRRICPRDYNMFRCVARYGYKDIRKEHYEAFEQLLIESLEKFLRKEALELALENESLIVDSDNESVDSKGAGP

SNGFADSLRTPLMEDQRHAQYGAPSRRLSGASTSSGLPTSVMSADEDPSLEYDLSSLREAKESGVVYLVGHGDVKARKDS

WFLKKLVINYFYAFMRANCRAGPANLSVPHTNLLQVSMTYTV

>PtHAK4

MNLQRTVMDVEVETAALRSSGRDKVSYRTALALAYQSFGVVYGDLSTSPLYVYTSTFSGKLKLREHDEEILGVLSFIFWT

FTLVPLCKYIFFVLTADDNGEGGTFALYSLLCRHANLSLLPNQQPLDAQLSTYNVTRPRETGPSCAIKSFFEKHQRQRIW

LLLVVLLGTCMVIGDGVLTPTISVLSAVQGIKVKVAHFDDRYIVLITCVILVGLFALQHLGTRNVGFMFAPIIITWLLCI

SSIGIYNIIRWNPKVFRAISPYYMFNFLRKTREVGWESLGGIVLCITGAEAMFADLGHFSQLSIKIAFTAVVYPCLILAY

MGEAAFLSQHKEDLQSSFYKSIPEAVFWPVFIVATLAAIVGSQAVISATFSIINQCSALCCFPRVKVVHTSKQIYGQIYI

PEINWILMCLCLALTIGFRNTTTIGHAYGLAVTTVMFVTTCLMVLVIIIVWEKSIYAAGSFFVIFGSIELLYISACLVKV

PEGGWVPLVLSFIFMAIMYVWHYGTSKKYEFNLQNKVSMERILSLGPGLGIARVPGIGFIYTELVTGIPAIFSHFVTNLP

AFHQVLVFVCIKSVQVPHVPLDERFLVGRVAPKEYRMFRCIVRYGYKDIQQENYDFENNLILNIAEFIQKESEQICGTHS

NGSLDCNERMSVISTPAESLMKMVVSDRQEEDSPRPHSRSPVSTPAIDTGSRKKKKVRFEVPESPAMDLAVKSELKELFE

AKEAGVAYIIGHSYVKAKASSFMLKKFAIDIAYSFLRTNCRDPSSLLSIPHISLIEVGMIYYV

>PtHAK5

MENRNIPLCTNRIDWYYPQMDLEVGAYAHVAQKESWKTILTLAYQSLGVVYGDLSISPLYVYRNTFAGDIEHSDTNEEIY

GVLSFVFWTLTLIPLFKYVFIVLKADDNGEGGTFALYSLLCRHAKISLLPNCQVADEELSTYNIDDFPQTKTGSRVKSVL

EKCRFFHTMLLIVALIGTCMVIGDGVLTPAISVFSAVSGLELSMSHHEYAVVPIACLILVGLFALQHYGTHRVGFMFAPI

VLTWLLCISAIGLYNIIHWNPHVYQALSPYYMYNFLKKTQRGGWMSLGGILLCITGSEAMFADLGHFSQLSIKIAFTSLV

YPALILAYMGQAAYISTHHDFTGAYQIGFYVSVPEHLRWPVLAIAILASVVGSQAVITGTFSIIKQSLALGCFPRVKVVH

TSDKIYGQIYIPEINWILMSLCLAVTIGFRDTKHMGNASGLAVITVMLVTTCLMSLVIILCWHKNALLAFLFILCFGSIE

ALYFSASLIKFLEGAWVPLVLAFIFMFVMYVWHYGTIKKYEFDLQNKVSMKWLLGLGPSLGIVRVPGIGLIHTELVTGVP

AIFTHFVTNLPAFHQVLVFVCIKSVPVPYVRPEERYLIGRIGPKEYRLYRCIVRYGYRDAHIDDHEFENQLVFSIGEFIR

SERQNLSPNVDSSQDGRMTVIGTPRREDMISDETDECMQSASASNPRSPIIQSIHDIIEVNSPKLVRRKKVRFLLPNSPE

MDRTVRDELQELSDAKEAGTAFIIGHSYVKAKRGSSFLRKFVINVAYEFLRKNCRGPSAALNIPHISLLEVGMVYQV

>PtHAK6

MDPEAGVYNKVRLLLTLMNKYQKPSCKTILMLAYQSLGVVYGDLSTSPLYVYKSTFSGKLRLHENDDEILGVLSFIFWTF

TFIPLFKYIFIVLNADDNGEGGTFALYSLLCRHAKMSLLPNQQAADEQVSTYKVERPCETPLSSAIKAFFEKHHRFRFVL

LVVVLLGTCMVIGDGVLTPAISGTKNLVTVTLSKDESNIANQELEVLSAVSGVKVKIHNLHENYVVAIACAILVGLFALQ

HYGTHRVAFLFAPVVIAWLICISGVGIYNIIHWNPKVFRALSPYYMYNFLRKTNVEGWVSLGGVVLCITGAEAMFADLGH

FSQLSIKIAFTGLVYPCLILAYMGEAAFLSKHKWDLQRNFYKAIPGKPQMLISFIFDALCLKFFLDDLVRFLAWPPPWLK

FVLLSEAVFWPVFIVATLASVVGSQAVISATFSIISQCSALSCFPRVKIIHTSNQIFGQIYIPEVNWILMCLCLAVTIGF

RDTNLIGHAYGLAVVTVMFVTTCLMSLVIIIVWQKNILVALSFLVIFGSIELLYISTCLIKVPEGGWVPLALSLIFMAIM

YVWHYGTLKKYEFDSQNKVSMKRILSLGPSLGMVRVPGIGLIYTDLVTGVPAIFGHFVTNLPAFHRVLVFVCIKSVQVPY

VPSEERYLIGRIGPKEYRMFRCIVRYGYKDVHQENYDFENQIVQNISEFIQKDNEEDSLDYNTEMCSTARMAVFSTSTDS

VMKIVLSDQENELSPVHKLHVRSSEIDSFTPRKKEVRFELLKSPDIDPRVRQELIDLVEAREAGVAYMLGHSHVKAKKSS

SILKKFTIDIAYNFLRKNCRGPDVAFNIPHISLIEVGMIYYV

>PtHAK7

MEDAGESEHAPADSKANYGGALEVVSIQNEKGMIDKGKPDNYRTMMQSYEIPFHKPKIFGKRETLFLAYQTLGVVYGDIG

TSPLYVFSSVGVENPKEREVLGILSVIFWTLTMIALVKYVMIVLRADDHGEGGTFAVYSLLAQHVNLEKSTGKQFRRLAS

DTRLKFFSTENGGKAINSKTKELLEKSATAQRILLIVVMMGTCMVIGDGALTPAISVMSAVQGIQARNNNMSQGVVVLVS

FIILLLLFLFQKLGTSKVSFLFSPIMIAWFVTNTMIGLYNIVKYYPGAFKAFNPYYIYYYFHEYHKQGWVMLGGVVLCIT

GAEAMFADLGHFNRKSIQLAFCVLVYPSLLITYTGQAAYLIKNPGDIGSTFYKAIPQSVYWPMFIVATLAAIVASQALIT

ATFSIIKQSMALSCFPRVKIVHTSQNYEGQIYSPEVNYVLMVICLAIVVGFRDGTQIGNAYGVAVVGVMFVTTCLVTLVM

LVVWNMHVLLILPFTLFFAIFEGIYLSSVLNKVPQGGWCPFLIAAIFLTIMFSWNYGRQKKYKYLARRKLSIEGLNGLVA

GVNSRVPGICFFCSDLISGLPPIIEHYVKNVGTLHEVLVIITIRMVPVKTVLLSERFMVGKLEPKGVYKCVAQYGYKDVP

NMEGSEFINQVVESLKDYLKLRGTHSSSHDISYASRNTVAESNYSVDEELQQLELANNTGVVYVLGKITLKTNKSTGPFE

GLLIDKLYRFLQNNCRSTLSTYNIPPAHLLQVGMVQEI

>PrpHAK1

MDLERVKCWDTSKKDSWKNILLLAYQSLGVVYGDLSISPLYVYKSAFAEDIQHSETNEEIYGVLSFVFWTLTLVPLFKYV

FIVLRADDNGEGGTFALYSLICRHAKVSLLPNRQLADEALSTYKLEHPPEKEKSSRLKVVLEKCKALHTALLILVLLGTC

MVIGDGVLTPAISVFSAVSGLELSMSKEHHQYAVVPITCFILLCLFALQHYGTHRVGFFFAPVVLAWLLCISALGLYNIF

QWNRYIYQALSPYYMFKFLRKTRISGWMSLGGILLCITGSEAMFADLGHFSYSAIQVAFTFLVYPALILAYMGQAAYLSQ

HHHTSHRISFYVSVPESVRWPVLVLAILASVVGSQAIISGTFSIINQSQSLGCFPRVKVVHTSDKVHGQIYIPEINWMLM

ILCIAVTIGFRDTKHLGNASGLAVMAVMLVTTCLTSLVIILCWRKPPILALCFLIFFGSIELLYFSASLTKFREGAWLPI

LLALFLMTIMFVWHYATIKKYEFDLHNKVSLDWLLALGPSLGIARVPGIGLVFTDLTSGIPANFSRFVTNLPAFHRVLVF

VCVKSVPVPYVLPAERYLVGRVGPAAHRSYRCIVRYGYRDVHQDVDSFESELVDRLADFIRYDWCRTQRTSSCTEDDASR

STDMSDSRLAVIGTVAFSGAPAYEIEETQPASVSVGFPTVESITDVIEMEPVERRVRFVIDDDSQADSRTENAMQIREEL

EDLYAAQQAGTAFILGHSHVKAKQGSSIMKRLAINFGYNFLRKNCRGADVALKVPPVSLLEVGMVYVV

>PrpHAK2

MASRVEIDEDSDTNKGSMWVLDQKLDQPMDEEAGRLRNMYREKKFSALLLMRLAFQSLGVVFGDLGTSPLYVFYNTFPHG

ISDPEDVVGALSLIIYSLTLIPLLKYVFVVCRANDSGQGGTFALYSLLCRHANVKTIPNQHRTDEELTTYSRSTFHEQSY

AAKTKKWLEGHASRKNALLLLVLVGTCMVIGDGILTPAISVLSAAGGIKVSSPGMKNDYVILVAVVILVGLFSVQHYGTD

KVGWLFAPIVLLWFLLIGGIGIFNIWKHDSSVLRAFSPVYIYRYFKRNGRDGWTSLGGIMLSITGTEALFADLAHFPVSA

VQIAFTTVVFPCLLLAYSGQAAYLMKNHDNKTVLQAFYLSIPEKIYWPVFIVATLAAVVASQATISATFSIIKQALALGC

FPRVKVVHTSKKFLGQIYIPDINWILMILCIAVTAGFKNQSQIGNAYGTAVVVVMLATTLLMTLIMILVWRCHWILVLIF

TGLSLVVECTYFSAVLFKVDQGGWVPLVIAAAFLLIMYVWHYGTLKRYEFEMHSKVSMAWLLGLGPSLGLVRVPGIGLVY

TELASGVPHIFSHFITNLPAIHSVVVFVCVKYLPVYTVPEEERFLVKRIGPKSFHMFRCVARYGYKDLHKKDDDFEKKLF

DNLFMFVRLESMMEGCSDSDEYSIYGQQTERSMEGLINNNGNTIGSTADLTISSVDSIVPAKSPLHANNTMSSSSQQSMQ

NEIDELEFLNNCRDAGVVHILGNTVVRARRDSRFYKKIAVDYIYAFLRKVCREHSVIFNVPHESLLNVGQIFYV

>PrpHAK3

MKEEDGIVERSERLVVRSGSGGGSDSRWVDGSEVDSESPPFSMLSENIGREGYGSLRRRLAKKPKRVDSFDVEAMEIAGG

GSHHSKDASVWSTLALAFQTLGVVYGDMGTSPLYVFADVFSRVKIESDVDVLGALSIVIYTIALIPLAKYVFVVLKANDN

GEGGTFALYSLICRYAKVNLLPNRQPADEHISSFRLKLPTPELKRALRIKETLERRSFLKTLLLLFVLMGTSMVIGDGIL

TPAISVMSAVSGLQGEVPGFGTTAVVVVSIIILVVLFNIQRFGTGKVGVMFSPILALWFFSLGSIGIYNLVKYDITVLKA

FNPAYIYFFFKKNDKEAWLALGGCVLCITGAEAMFADLGHFSVRAIQIAFSFVVFPCLLLAYLGQAAYLMKYPDSASRIF

YNSVPGILFWPVFVVATLAAMIASQAMISATFSCVKQSMALGCFPRLKIVHTSRRRMGQIYIPVINWFLMIMCIVVVSIF

QSTTEIANAYGIAEVGVMMVSTTLVTLVMLLIWQTNLFLALCFPLVFGSVEFIYLCAVLSKIFEGGWLPLVFAICFLCVM

YTWNYGSVLKYRSEVREKISMDFMTDLGSTLGTVRVPGIGLLYSELVQGIPSIFVQFLLSLPAIHSTIVFVCIKYVPVPV

VPQEERFLFRRVCPKDYHMFRCIARYGYKDIRKEDQYAFEQLLVESLEKFLRREAQDLALESNLNDSDVDDVSPRSWDSG

VPGGDEIEELKIPLMHNGRLQDVGTSTSEETTAGTALPSSVMPSDEDPSLEYELSALREAIDSGFTYLLAHGDVRAKKNS

FFFKKLVINYFYAFLRKNCRAGAANMSVPHMNIIQVGMTYMV

>PrpHAK4

MEPESGISTPSRNPPPQLSWVNLSRNLILAYQSLGVVYGDLSTSPLYVYTSTFIGKLQNHNNEEVIFGAFSLIFWTLTLI

PLLKYVFILLSADDNGEGGTFALYSLLCRHAKFSLLPNQQAADEELTAYKYGPSSQVVASSPLKRFLEKHKRLRTALLVV

VLLGACMVIGDGVLTPAISVLSSVSGLQVTEEKLTDGELLLLACVILVGLFALQHCGTHRVAFLFAPIVIIWLISIFAIG

LYNTIHWNPAIVRALSPHYIVKFFRETGKDGWISLGGILLSITGTEAMFADLGHFTALSIRLAFAFIIYPCLVVQYMGQA

AFLSKHPNCIRYSFYDSIPEPVFWPVFVVATLATIVGSQAVITATFSIIKQCHALGCFPRVKVVHTSKHIYGQIYIPEIN

WILMILTLAITIGFQDTTLIGNAYGLACMMVMFITTFLMALVIVFVWQKSVVLAAVFLIFFWFIEGVYLSAALIKVPQGG

WVPFVLSFIFMIVMYVWHYGTRKKYNYDLHNKVSLKWLLGLGPSLGIVRVPGIGLIYSELATGVPAIFSHFVTNLPAFHK

VLVFVCVKSVPVPSVSPEERFLIGRICPRPYRMYRCIVRYGYKDIQRDDGDFENQLIQSIAEFIQMEAVEPQFSSSESAS

FDGRMAVISTRTGQSSSSLIANEQEVFGVSDSIQSSKSLTLQSIGSVYDDENPQIRRRQVRFQLPSNPGMDPAVREELMD

LIQAKEAGVAYIMGHSYVKARRSSSYLKKLVIDMGYSFLRKNCRGPAVALNIPHISLIEVGMIYYV

>PrpHAK5

MARRMSADENTDNKGSVWALDQQLDQPMDEEAKRLRNMYKEKKYSTLMLLQLAFQSLGVVYGDLGTSPLYVFYNTFPDGI

GDPEDLIGALSVIIYSLTLIPLLKYVFIVCRANDNGQGGTFALYSLLCRHAKIKAIPNQDQTDEALTTYSRSTFGEQSFA

ARTKRWLEGHALMQSTLLILVLVGSCMVIGDGILTPAISVLSAVGGINVGHPKISNGVVVLVAVVILVLLFTMQRHGTDK

VGWLFAPVVLLWFLVIGGIGMFNIWKYDRSILKAFSPVYVYRFFKRGGKDGWTSLGGIMLSITGTEALFADLSHFPVPSI

QIAFTSVVFPCLLLAYCGQAAYLMKNSNNVIGAFYHSIPDSIYWPVFIVATAAAVVASQATITATFSLIKQALALGCFPR

VKVVHTSRKYRHQIYIPEINWIVMILCIAVTAGFKNQNQIGNASGTAVCIVMLVTTLLMILVMILVWRCHWILVLIFTGL

SLVVEGTYFSAVLLKVNQGGWVPLVIAAAFFVIMYVWHYGTVKRFEIEMHSKVSMAWILGLGPSLGLVRVPGIGLVYSEL

ANGVPRIFSHFITNLPAIHSVVIFVCVKYLPVCTVPEEERFLVKRIGPKNFHMFRCVARYGYKDDHKKDDDFEKKLFQSL

FMFVRLESLMEASSDSDVSSLLEQQTKQSEDGLFCNSSNILHSDVDLSIASVDAIVPDDSPLHSNNMTSFVPASSKVETD

EIEFLNNCRDAGVVHMLGNTVVKARRESKFWKKIAIDYLYAFLRKICRENSVMFNVPHESLLNVGQVFYV

>PrpHAK6

MDPETGIYKNHVKKESWRTILTLAYQSLGVVYGDLSTSPLYVYKSTFAEDIEHSETNEEIFGVLSFVFWTLTLVPLLKYV

FIVLKADDNGEGGTFALYSLLCRHARVSSLPNCQLADEELSDYKKENTGSSLQSSFGTRLKSTLERHRVLQRFLLVLALI

GTCMVIGDGVLTPSISVFSAVSGLEFSMSEKHHKYVEVPIACIILIVLFALQHYGTHRVGFLFAPIVLTWLLCISAIGLY

NILHFNPSVYKALSPHYMYKFLKKTQKGGWMSLGGILLCITGSEAMFADLGHFSQLSIKIAFTSLVYPALVLAYMGQAAY

ISSHHTVGSNPKIGFYVSVPEKLRWPVLVIAVLAAIVGSQAIITGTFSIIKQCSSLDCFPRVKIVHTSSKIHGQIYIPEI

NWILMVLCLAVTIGFRDTKRMGNASGFAVISVMLVTTCLMSLVIVLCWQQSVFVAICFVIFFGTIEALYFSASLVKFTEG

AWVPVALALSFFIVMYVWHYGSIKRYEFELQNKVSINWLLSLGPSLGIVRVRGIGLIHTDLVSGIPAIFSHFVTNLPAFH

QVLVFLCIKSVPVPHVRPEERFLVGRIGPKEYRLYRCIVRYGYRDVHKDDMEFENDLMCSIAEFIRSGSPESNVTSEDLG

KEDDKMTVVGTRSTHADGIQMSEHSTEMNESVQREIRSPPPTQRRKKVRFIVPESPKINMGTREELQELMEAREAGIAYI

LGNTYMNAKKGSSWMRRFAIDYGYEFLRRNSRTSSYALSVPHASTLEVGMIYHV

>PrpHAK7

MAEEEGLERGEINGGLASMDSIDSRWVFQDEDDSEVDDEEAEDDVRHRTVVDSEDDEDDDNAEQRLIRTGPRIDSFDVEA

LEVPGALRNEYEDFSLGRKIIIAFQTLGVVFGDVGTSPLYAFSVMFKKAPINGNEDVIGAMSLVLYTLILIPLLKYVLVV

LWANDDGEGGTFALYSLICRHAKVSLLPNQLPSDARISSFRLKVPSPELERSLKIKERLEASLTLKKLLLTLVLAGTAMV

IADGVVTPAMSVVSAVSGLKIGVDAIKQDQVVMISVTFLVILFSVQKFGTSKVGLAVGPALFIWFCSLGGIGIYNLVKYD

SSVLKAFNPVHIYYFFKRNSTKAWYSLGGCLLCATGSEAMFADLCYFSVRSVQLTFVLLVLPCLMLGYLGQAAYLMENPD

GAEQAFFSSIPNAVFWPVFLIANIAALIASRAMTTATFSCIKQSMALGCFPRLKIIHTSRKFMGQIYIPVVNWFLLVVCL

VSICTISSIDEIGNAYGIAELGVMMMTTILVTIVMLLIWQINIIIVLSFIVIFLGLELTFFSSVLWSVGDGSWIILVFAI

IMFFIMFIWNYGSKLKYETEVKQKLSMDLMRELGCNLGTIRAPGIGLLYNELVKGIPAIFGHFLTTLPAIHSMIIFVCIK

YVPVPVVPQSERFLFRRVCPKNYHIFRCIARYGYKDVRKESHQTFEQLLIESLEKFIRREAQERSLESDGDDGDIDSEDV

TSCSRVLIAPNGSVYSLGVPLLAEYKESSEPISEASTSEEVKPGPPADQTAYDAEQSIERELSFIRKAKESGVVYLLGHG

DIRARKDSWFIKKLIINYFYAFLRKNCRRGIANLSVPHSHLMQVGMTYMV

>PrpHAK8

MTENEDPEKKGGVWALDQQLDQPMDEEANKFSNMSEDKKCSSITVLQLAFQSLGVVYGDLGTSPLYVFYNTFPYGIDEPD

DLLGALSVVIYSLTLIALVKYVLIVCRANDNGQGGTFALYSLLCRHAKIKTVPNQDQSDEELTTYSRSTFGEQSFAASTK

RWLEGHALIQNVLLILVLIGSCMVIGDGILTPAISVLSAVGGINCCRFHTSNDIVVVVAVVILVILFIMQQFGTQKFGWL

FAPIVLLWFLLIGGIGIFNIWKYDTSILRAFSPVYVIAFFRKGGTHGWASLGGIMLCINGTETLFADVSHFPVLSVQIAF

TLVAFPCLLLAYSGQAAYLMKYPHHVFGAFYYSIPESIYWPVFSVATAAAIVASQATITATFSLIKQALALGCFPRVKVV

YTSRSHHQIYIPDINWILMILCISVTAGFKNQSQIGNAAGTAVVIVMLVTTFLMILVMILVWRCHWTLVLFFACLTLVVE

GTYFSAVLLKVNQGGWVPLVIAVAFFIVMYGWHYGTVIRFDIEMHSKVSMAWILGLGPSLGLVRVPGIGLVYSEIATGVP

HIFSHFITNLPAIHSVVVFVCVKYLPVCTVPEEERFLVKRIGPNNFHMFRCVVRYGYKDDHKKDDDFEEKLFDNLFTFVR

LESLMEGSPGSDVSSILNQQTIQSRDAMWNNNNNRSIPYSSADVSITSVDSIELADSPIYVSQPLTCSIPTSSQLVEIDE

IEFLNRCRDAGVVHILGNTVVRARRDSNCFKKMAVDYLYAFLRKICRGNSVLFNVPHESLLSVGQIFYV

>PrpHAK9

MAEKVVSLREGEAELTNQHLKERKASWAKLRRVDSLSLEAGRVSTTRSHGSQVSWQRTLSLAFQSIGIVYGDIGTSPLYV

FSSTFTDGIDNVDDILGVLSLIIYTIALVPMLKYVFIVLWANDNGDGGTFALYSLMCRYAKVSLIPNNQPEDRELSNYKL

ETPSNELKRAQTIKKKLENSKMAQYVLFLVTIMGTSMVIGDGVLTPCISVLSAVSGIKSLGTDAVVGISIVILIVLFAAQ

RFGTDKVGYTFAPIILLWFLLISGIGLYNLFKHDVGVLRAFNPKYIIDFFKRNGKKGWVSLGGVFLCITGTEAMFADLGH

FNVKAIQISFSCITFPALITAYSGQAAFLRKFPGKVEHTFYDSIPDPLYWPTFVIAVAAAIIASQAMISGAFSIISQSLS

LGCFPRVKVVHTSAKYEGQVYIPEINYLLMVACVIVTAAFKTTGKIGNAYGIAVVSVMVITTCLLTLIMLVIWKTSIWLI

ALFFVVFISIEVVYSSAVLYKFAQGGFLPLVFASLLMAIMGIWHYVHKQKYMFELNNKVSTEYMKQLACNPNINRVPGIG

LLYSELVQGIPPIFSHFVNNVPSIHSVIVIVSIKPIPFSKVALEERFLFRQLEPREYRMFRCVARYGYNDRVEEPEEFER

QLVENLKEFIRHEVHEHFVLDGGVTEKTEEARKDGESFNGEESAQQVNPPRVSTGSIHRIVSAPIKGAEEEIQFVQGAMK

DGIVYLLGETEKFVVNYAYDFLRKNFRQGEQVMAIPRTRLLKVGMTYEI

>PrpHAK10

MNPSEELMEQGISPRSMQKVSYTTVLTLAYQSLGVVYGDLSTSPLYVYKTTFSGKLSLQEDDEEIFGVLSFIFWTFTLIA

LCKYVFIVMLADDNGEGGTFALYSLLCRHARLSILPNQEITDEKLSEYVTEGTTDTWQSSALKLFFNKHPGFRKGLLVFV

LFGTCMAIGDGVLTPAISVLSAVSGIKLKFTELHDNYVVIISCVLLVGLFSLQHHGTHRVAFMFAPIVTAWLLCISGIGI

YNIFHWNRRIFHALSPVYMLKFLRSTGVEGWVSLAGVVLSITGVEAMFANLGHFSSLSIKMAFTLLVYPSLVISYMGEAA

FLSKHHEDIQRSFYKAIPEAVFWPVFIIATFASVVGSQAVISATFSIISQCCALKCFPRVKIVHTSSKIYGQIYIPEVNW

MLMCLCLAVTIGLRDTNMMGHAYGLAVTAVMFVTTCLMALVMIIVWKKRILNAVAFLMLFGSIELIYIAASISKIPEGGW

IPVVLSLIFMGVTYIWNYGTMMKHQFDVENKVSINRIVCLGPSLGMVRVPGIGLVYTNLVTGIPPVFGHFVTNLPAFHKV

LIFVSVKSVQVPHISGKERLLISRVGPKECGMFRCIVRYGYKDLQQETCNFENILVSSILQFVETEEECPLEPVTGCSRE

FADAEARHAPDHTLVDSNDEENMETSLSKDESLRILEAKESGVTYILGHSHAKAKKSSSFFKKVAIDIVYAFLRKNCREP

DVVLNVPHTSLLEVGMIYYV

>PrpHAK12

MPDEEVILENATDHQEVPDHHQNQELKSKKLSWQKLRRYDSLDLESRSFTAHHGHASKGAEWSVILHLAFQSIGIVYGDI

GTSPLYVYSSTFTKGINHDDDILGVLSLILYTLTLIPLIKYVFVVLRANDNGDGGTFALYSLLCRYAKVGLTPSQQAEDR

DVSNFELELPSKRLKRASRLKSKLENSPFAKVFLLFATMLGTSMVIGDGVLTPCISVLSAVGGIKEATSAMTEDRIVWIS

IAILICLFMVQRFGTDKVGYTFAPIICVWFTLIGGIGVYNFIKFDPTVVKALNPQYIVDYFRRNKKDAWISLGGIVLAIT

GTEALFADVGHFTVRSIQISMCAVTYPALILAYTGQASFLRNHHHLVYETFFKSIPGPLYWPMFVVAVLASIIASQAMIS

GTFSIIQQSLSLGCFPRVKIVHTSARYAGQVYIPEVNYLLMLACVGVTLGFRTTAKIGNAYGIAVVFVMTLTSSFLVLIM

IMIWKTNIFLVISYVLVIGSVELMYLSSVLYKFDQGGYLPLAFAMVLMIIMFVWNDVHRRKYYYELDHKISPVQLKEIAV

SANFCRMPGLAMFYSELVQGIPPIFNHYAANVPALHSVLVFVSIKSLPISKVPLEERFLFRRVEPKELNVFRCVARYGYT

DVRNEHEPFEGLLVEKLKEFIKDSFWISQRNMDDNNGEKFDIKEEEFDDGLANGENGNEDVKQVDDQEKQQDLLDEDIEA

IDKAWRWGVVHLIGENEVTAAKGAGIVKRILIDYAYNFLKRNLRQSDKVFDIPHKRMLKVGMTYEL

>PrpHAK13

MGEKAETDDKMRRTDSLNLEAGKVSTAHDHGSKLSWKRTMSLAIQSVGVVYGDIGTSPLYVFSSTFPKGINHKDDILGVL

SLIIYTILLVPLVKYVLVVLWANDNGEGGTFALYSLICRYAKVSLIPNTQPEDRELSNYKLELPSNELKRAQAIKKKLER

TKSAKYALFVITIMGTSMVIGDGILTPCISVLSAVSGIKSLGTDAVVGISVVILVLLFAVQQFGTDKVGFTFGPIILLWF

VFISCIGLYNLITYDVTVLRAFNPAYIYHYFHRNGKEAWISLGGVVLCITGTEAMFADLGHFSVRAIQISFTCFTFPTIL

FAYFGQAAYLTKYPEKVTDTFYASIPSPMYWPTFVVAVLAAIIASQALITGTFSIISQSLSMGCFPRVKIVHTSAKNEGQ

VYIPEINYILMIFCVIITAAFKTTEKIGNAYGIAVVSVMVITTCMLTLIMLVIWKISIILIAIFFVIFIAIEGVYLSAVL

FKFSEGGYLPLCFAAVLMMIMAIWHYVHKQCYTYEANNKVSSEYMKQLSSNPNINRVPGIGLLYSELVQGIPPIFSHFVS

NIPSVHSVVVVVTIKPLPVSKVLLEERFLFRQLEPKDYRMFRCVARYGYNDRVEEPAEFERQLVENLKEFICHQHLMPSE

DQGVSGRQKAEEETQQSESVMAIPRTRLVRVGMTYDI

>PrpHAK14

MDPQLGSANQFKETWKHTLLLSFQSLGVIYGQLSTAPLYVFGTMNAEDIKSEETVYELFSCIFWTINIISLLKYAFIVLR

ADDNGEGGTFALYSLLCRHAKVGLLPNDTSANEVMHYETGSPFKIKVESRARRAIEKHKSSHYLMLFLALFGSCMTIGVG

VLTPALSVYSVSSGVQRSMSDMAHLYVPVPTASAILVCLFTLQHYGTHKIGFIFAPIIVIWLIFIGGGGIYNIFHWNKQI

IHAVSPMYMYRFVKNIEIKSWRSLGSIVLCVAGSEAMFADLGHFRKKSIKITFVCLIYPVLVLCYAGQAAYISKNLHAAD

FNHLSESIPPLSLLASVVGSQATITASFSIINQCLALGCFPRVKVIHTSDKIHGQVYIPDINWLLMVLSLAVTIGFHDIM

RIGSATGLAVISGMLVTTCLMSLVIALYWEKNLFESVCCLIFFGSIEVMYVSACMLNFHKGAWYLVVLLALSLTVMLSWH

YGTKKKLEFDLQNKVSAEWLTDISPGLGVTRVPGIGFIYTDIVTGIPAFFSHFITNLPAFHQVLIFVSFKSLPMPYVPAS

RRYLIGRVGPKDLKIYRCVVRYGYCDPIRDTDNFEEQIISSIGEFITMEENEFESLNSSEGRMVVVGKPPADGSALIPLN

ETNSDEESVSLVSNIETQLAPMVADAVESGLGSVMRKKVRFMLPAKSPKMRASVRDELQELIDARESGTAYFLGQVHLAV

RDGSDVLKRLLIMTYAFCDKNCREPPVALNIPHAALVEVGMVCCI

>ZmHAK1

MSLEVDNPAGGAGGGGESANRLSLKRHDSLFGDAEKVSGGKYHGSEGSWARTLHLAFQSVGIIYGDIGTSPLYVYSSTFP

DGIKYNDDLLGVLSLIIYTLIIIPMLKYVFVVLYANDNGDGGTFALYSLISRYAKIRLIPNQQAEDAMVSNYSIEAPTSQ

LKRAQWFKQKLESSRAAKILLFTLTILGTSMVMGDGTLTPSISVLSAVSGIKEKAPNLTQTQVVWISVAILFLLFSVQRF

GTDKVGYTFAPVISVWFVLIAGIGLYNLVVHDVGVLRAFNPWYIVQYFKRNGKEGWVSLGGVILCVTGTEGMFADLGHFN

IRAVQISFNGILFPSVVLCYIGQAAYLRKFPENVGDTFYKSIPAPLFWPTFIIAILAAIIASQAMLSGAFAILSKALSLG

CLPRVQVIHTSKKYEGQVYIPEVNFMMGLASIIVTIAFRTTTSIGNAYGICVVTTFSITTHLMTVVMLVIWKKHIVYVLL

FYVVFGLTEMIYLSSILSKFIQGGYLPFCFALVLMTLMATWHYVHVKRYWYELDHIVPTNQMTALLEKNDVRRIPGVGLL

YTELVQGIPPVFPRLIKKIPSVHSIFLFMSIKHLPIPHVLPAERFLFRQVGPREQRIFRCVARYGYSDALEEPKDFASFL

ADRLKMFIQEEVAFAQNDAENDDEAATDHQAPPRPPRRSTGSVVHSEEAIQSRGSTHSGRITFHASQTAEEEKQLIDREV

ERGVVYLMGEANVSAGPNSSVLKKIVVNYIYTFLRKNLTEGHKALAIPKDQLLKVGITYEI

>ZmHAK2

MDAEAGVAGADQLPRRQYYLNLLLLAYQSFGVVYGDLSTSPLYVYKSTFSGKLRHYQDEETVFGVLSLIFWTFTLIPLLK

YVIIVLSADDNGEGGPFALYSLLCRHAKLSLLPNQQAADEELSSYYRNGFAPRNGSSPWSRRFLEKHKKMRTLFLLIVLC

GASMVIGDGVLTPAISVLSSMSGLHVRATGLHHSSVVLLSCIVLVGLFALQHRGTQKVAFMFAPIVIIWLLSIGGIGLYN

ILHWNPNVYQALSPYYMVKFFRKTGSEAMFADLGHFTSASVRVAFITVIYPCLMLQYMGHAAFLSKNTFHMPTGFYDTIP

EPVFWPVFVVATLAAVVGSQAVISATFSIVKQCHALGCFPRVKVVHTSRWIYGQIYIPEINWILMVLCVAVTIAFRDTTL

IGNAYGIACMTVMLVTTFLMALIVIFVWQRNIIFSLVFLVFFGSIEAVYLSSSLMKVPQGGWVPLVLAFIFMSVMYIWHY

GSRRKYQFDLQNKVSMRSILSLGPSLGIVRVPGVGLIYTELVTGVPSIFSHFVTNLPAFHEVLVFLCVKSVPVPYVSPDE

RYLVGRIGPKEYRMYRCIVRYGYKDVQRDDDNFENMLVMSIAKFIMMEAEDASSASYDIANEGRMAVITTTDASGSPLAM

RDFDGLADSMSTRSSKSESLRSLQSSYEQESPSVNRRRRVRFEVPEEDGMGQQVKEELTALVEAKHAGIAYIMGAFLYQS

QEELKFPEEVCY

>ZmHAK3

MWLTDPEYGLSPKNTIGIDVYNPPPLHWTSLLVLAYQSCGVVYGDLSTSPLYVYKGTFSGSLHRFLDEETVFGVFSVVFW

TITLIPLLKYVFIVLSADDCGEGGTFALYSLLVRHAKFSLMPNQQAADEELSAYYRPGYSTEETPILKALRNFLEKHRKS

RTFLLLMVLFGASLVIGDGVLTPAMSVLSSFSGLQVHSNALTHGEVVLLSCIVLVCLFTLQHWGTRRVAFLFAPVVVLWL

LLLAALGIYNIAVWNPRILRALSPYYVVRFFQRTGKDGWISLGGVLLSMTGTEAMYADLGHFTAASIRIAFVGLIYPCLV

LQYMGQAAFLSKSPDCNIHFIFFESIPRPIFWPVLVIATLAAIVGSQAVISATFSIVRQCTALGCFPRVKIVHTSNRIHG

QIYSPEINWILMLICLGVTVGFRDTDLIGNAYGMACAGVMVVTTLLMALVMVFVWQQGFILAAMFLLAFGSVESVYLSAA

LMKVPQGGWLPLALSLVVVAIMYVWHYGTRRRHLFDVQNKVSLKWLHALGPSLGIVRVPGIGLIYSELATGVPAIFSHFV

TNLPAFHQVLVFVCVKAVPIPHVRCYERHLIGRIGPREFRMYRCVIRHGYKDVPGDDNDFENDLVVRIAEFVHMEAAEAA

AHADAPRCSDASVDGRMAVVNRPFDLSRTGLLMRAPLPNPEDSVVVRAATAVTTAGDSSKTETMQSLQTMYEAESPGFAI

RRRIRFEIDDSTSESMDPAVKEELSALVEAKHAGVAYIMGHSYIKARKSSSIVKKLAIDVAYSFLRKNCRGPAVALNIPH

ISLIEVGMIYYV

>ZmHAK4

MSRILATNLPQQKKRIYKDLLLAYKTLGVVFGGLVTSPLYVYPSMNLTNPTEEDYLGIYSIMFWTLTLIGVVKYICIALN

ADDHGEGGTFAMYSLLCQHANIGILPSKKIYTEEEQGLVPARPVAARRPSKVRRFIERSITARRLLQLTAILGMCMLIGD

GILTPAISILSAVDGLRGPFPSVSKPTVEALSAGILIGLFLLQKYGTSKVSFMFSPIMAAWTFTTPIIGIYSIWRYYPGI

FKSVSPYYVVHFFVTNRKRGWQLLGGTVLCITGAEAMFADLGHFNKRSIQIAFLSSIYPSLVLTYAGQTAYLINHVGDFG

DGFYKFVPRPVYWPMFVIATLAAIVASQSLISATFSVVKQSVALDYFPRVRVVHTSKDKEGEVYSPETNYLLMLLCVGAI

IGFGDGKDIGNAFGVVVILVMLITTILLSLVMLIVWGTHVVLVALYLVPFLILEGTYVSAVCTKIMKGGWLPFAISLVLA

LVMFSWYYGRQRKAEYEMANKVTLERLSELLAAPDVRRAPGLCLFYSNMQEWRWLTPVLAHYIKNMRSLHGVTIFVTLRY

QLVAKVDAESRMAVRRFGPRGVYGCTIQYGYAGPLYEEEEEDLAGQVVRAVRQHIEREAAASSTAEVEEEAAELEEARAA

GVVHVMGKTRFHVGRNTGLFDRVLLGFYEFLHTTCRSALPALGIPLQQRVEIGMLYKA

>ZmHAK5

MAEPHHTSSNGAAHGDADDASEKMPPKRLQRYDSLHMEAGMFPAGGSTHAAKVGWATTLHLAFQSIGVVYGDMGTSPLYV

FSSTFTNGINNTDDLLGVMSLIIYTVILLPLIKYCFIVLRANDNGDGGTFALYSLISRYARVSLIPNQQAEDAMVSHYKL

ESPTYPMKRAHWIKKKMESSPKFKVLLFLVTVLATSMVIGDGVLTPSMSVLSAVGGIQQRATNLTQGQIVGISIAILIVL

FLVQCFGTDKVGYTFAPIILAWFLLIAGIGVYNLIKHDVSVLKAFNPKYIVDYFKRNGKQGWISLGGVILCITGTEAMFA

DLGHFNVRAIQIGFSVVLFPSVLLAYIGQAAYLRVYPENVANTFYKSIPGPLYWPTFVVAVAAAIIASQAMISGAFAIIA

QSQVLGCFPRVRVTHTSTKFEGQVYIPEINYVLMVLCVAVTAIFQTTEKIGNAYGIAVVFVMFITTLLVTLVMVMIWKTS

LLWIALFLVIIGGAELVYLSSAFYKFTQGGYLPLAFAGILMFIMATWHYVHVHRYNYELQNKVSSNYVAELSARRNLARL

PGIGFLYSELVQGIPPILPHLIERVPSIHSVLVIISIKYLPISKIETSERFLFRYVEPRDYRLFRCVVRYGYNDKVEDPR

EFEGLVIEHLKQFIHQESFYSQGDNSTEEVEDANEPSVRVQGATLSDSSSDRSTAAPSNGCIYEIQTIQREMEDGVVHML

GEANVVAEPNADFFKKIIVDYAYNFMRKNFRQPEKITCVPHNRLLRVGMTYEI

>ZmHAK7

MDEEVGAAGRQQVQWKSYCSTLSLLAFQSFGVVYGDLSTSPLYVYRNSLSGRLNGYLDETTIFGLFSLVFWTFTLVPLLK

YVIIVLSADDNGEGGAFALYSLLCRHAKFSLLPNQQAADEELSTYYHPGTDRAVVSSPFKRFLEKHRKLRTCLLLFVLFG

ACMVIGDGVFTPTISVLSAISGLQDPATSGLGDGWIVFIACVMLVGLFALQHRGTHKVAFLFAPIIVLWLLSIGIIGLYN

IIRWNPRIFVALSPHYIVKFFKKTGKDGWISLGGVLLAITGTEAMFADLGHFSAASIRLAFVSVIYPCLVLQYMGQAAFL

SKNIPAVYNSFYLSIPSPLFWPVFVIATLAAILGSQAIISATFSIVKQCLALGCFPRVKVVHTSRWIRGQIYIPEINWIL

MVLCLAVTLGFRDITVIGNAYGLACITVMFVTTCLMSLVIIFVWQKNLLISLLFFVFFGALEGAYLSAAVMKVPQGGWAP

IALSAVFMFIMYVWHYGTRRKYLFDLQNKVSMRWILNLGPSLGIVRVPGIGLIYTELVTGVPAIFSHFVTNLPAFHQVLV

FVCVKSVPVPYVPMDERYLIGRIGPREYRMYRCIVRYGYKDVQKDDENFENNLVMSIARFIQMEAEESASSGTGRSYESS

TEGRMAVVHTTGTTGTGLVMMASAEDAEGTSLSRSSKSETLRSLQSIYEQESAGTVSRRRRRVRFQIDEEERIEPQVRDE

LSDLLEAKEAGAAYIIGHSYVKARKNSNFLKTFAINYAYSFLRKNCRGPSVTLHIPHISLIEVGMIYYV

>ZmHAK8

MDLEFGRGLRSPKRDSWKTTLLLAYQSLGVVYGDLSISPLYVYKSTFAEDIQHSETNEEIFGVLSFVFWTLTLIPLVKYV

SVVLRADDNGEGGTFALYSLICRHANVSLLPNRQIADEELSTYKLECPPEITDKSRIKVWLERHRKLRVALLVMVMIGTC

MVIGDGVLTPAISVFSAVSGLEFSLSKDHREYAVIPITCVILAFLFALQHYGTHRVGFLFAPIVLAWLFCMSALGLYNII

HWNPQIYQALNPSYMFKFLKKTRKYGWMSLGGILLCMTGSEAMFADLGHFSYSAIQLAFTCLVYPALILAYMGQAAYLSK

HHDFYSSSQVGFYIAVPDKIRWPVLILAILASVVGSQAIISGTFSIINQSLSLSCFPRVKVVHTSEKIHGQIYIPEINWL

LMILCIAVTVGFRDTKHMGNASGLAVITVMLVTTFLTSLVIVLCWHRPPLLALAFLLFFGSIEALYFSASLIKFLEGAWL

PILLALILMAVMLIWHYTTIKKYEFDLHNKVTLEWLLALGDRLGMVRVPGIGLVYTDLTSGVPANFSRFVTNLPAFHQVL

VFVCVKSVPVPYVFPAERYLIGRVGPPGHRSYRCIVRYGYRDVHQDVDSFETELVETLATFITLDASYRCSEASERELEL

DPGEQERRLTVIASNPLRRRASYDLQDSVQHSAASTVEVRATAADSLSPRDAEISSAAGPKQVRFFIDSHVVSPEAAENK

QVADELEALAAAREAGTAFILGHSHVQCKPGSSVLKRLAVDVGYNFLRRNCRGPDVALRVPPASLLEVGMVYVL

>ZmHAK9

MDPEFGVGTAPQKGRRAWRTTLLLAYQSLGVVYGDLSISPLYVYKSTFAEDITHSETNQEIFGALSFVFWTLTLVPLLKY

ATIVLRADDSGEGGTFALYSLICRHANVSLLPNRQVADEELSTYRLERPREAAGRSGLLRAWLEKHTRLHTALLVMVMIG

TCMVIGDGVLTPAISVFSAVSGLELSLSKDQHEYAVIPITCAILVFLFALQHYGTHRVGFLFAPIILAWLLCMSAIGLYN

IVRWNPQIYQALNPVYMIRFLRKTKKSGWMSLGGILLCMTGSEAMFADLGHFSYSAIQLAFTSLVYPSLILGYMGQAAYL

SQHHNLDASYQIGFYIAVPECVRWPVLVLAILASVVGSQAIISGTFSIINQSQSLSCFPRVKVVHTSDKVHGQIYIPEVN

WILMILCVAVTVGFRNTKHMGNASGLAVITVMLVTTCLMPLVIMLCWDRSPWLALAFFLFFGSIEALYFSASLIKFLDGA

WVPILLALILLAVMFVWHHTTIRKYEYDMHNKVTLEWLLALGDKLGMVRVPGIGLVYTDLTSGVPANFSRFVTNLPAFHR

VLVFVCVKSVPVPHVLPAERYLVGRVGPPGHRSYRCIVRYGYRDVHQDVDSFETELVESLATFIKLDALFRCSDAAGDQQ

RDSSYYERENALTVIGSNPLRRHLALGYDDSHSHDDGASSAGSDRVDGIELAAAAPAPAVVKKQVRFAVAPPRSPGVDET

VLEELHELCEAREAGTAFILGHSHVKTKPGSSILKRLAVGVGYNFLRRNCRGPDVVLRVPPASLLEVGMVYVL

>ZmHAK10

MKSPPVMDPEAPPSPPLPGTPPDDEEEKRGGRNGVPWRMTLSLAYQSLGVVYGDLSTSPLYVYKAAFAEDIQHTESNEEI

LGVLSFVFWTLTLVPLLKYVCVVLRADDHGEGGTFALYSLLCRHARAALLPPGRTAAGDEDQFLDGADGGTKKATAQNGN

AVTLGGGAAASVRRLLERHKVLQRVLLVLALVGTCMVIGDGVLTPAISVFSAVSGLELSMEKEHHKYVELPIACIILVCL

FALQHYGTHRVGFIFAPVVVTWLLCISVIGVYNIIHWEPTVYRALSPYYMYKFLRKTQRGGWMSLGGILLCVTGSEAMFA

DLGHFNQLSIQIAFTCMVYPALILAYMGQAAYLCKHHTMESDYRIGFYVSVPEKIRWPVMAIAILAAVVGSQAVITGTFS

MIKQCTSLGCFPRVKIIHTSAKVHGQIYIPEINWILMILCLAVTIGFRNTKHLGNASGLAVITVMLVTTCLMSLVIVLCW

HKSIFLAIGFIVFFGTIEALYFSAALIKFREGAWVPIVLAFIFILIMCIWHYGTIKRYEFDVQSKVSINWLLGLSPNLGI

VRVRGIGLIHTELETGVPAIFSHFVTNLPAFHQVLIFMCIKNVPIPHVRPEERFLVGRIGPKQYRMYRCIVRYGYHDFHK

DDIEFEKELVCSVAEFIRSGSSKLNGMPEEFDEEEQRMAVVRSNSIRMLEEEATVEKTVGPSQASREIRSPSPTPPPVVG

PKKRVRFVLPAASPKPNAGVQEELQELSDAREAGMAFILGHSHVKAKSGSSFLRRFVINFCYDFLRRNSRGPNYAVSIPH

ASTLEVGMMYYI

>ZmHAK11

MSSMASLSESELTNRGSMWELDQNLDQPMDEEAGRLKNMYREKKFSSALLLRLAFQSLGVVFGDLGTSPLYVFYNIFPRG

IGEGEDEDVIGALSLIIYTLTFIPLLKYVFVVLRANDNGQGGTFALYSLLCRHAKINTIPNQHRTDEELTTYSRQTYEEN

SVAAKIKRWIESHAYKRNILLVLVLIGTCTAIGDGILTPAISVLSASGGIRVQNQNMSTDVVVLVAVVILIGLFSMQHYG

TDKVGLLFAPIVLLWFILIGSVGAINIHKYNNSVLRAYNPISIYRFFQRRRNYDIWTSLGGIMLSITGTEALFADLCHFP

VLAIQIAFTLIVFPCLLLAYTGQAAYIISNKTHVADAFYRSIPDAIYWPAFVIATAAAIVASQATISATYSIIKQALALG

CFPRVKIVHTSKKFLGQIYMPDINWILLVLCIAVTAGFKNQSQIGNAYGTAVVIVMLVTTFLMVPIMLLVWKSHWILVVT

FIVLSLMVEVPYFVACILKIDQGGWVPLVVATAFFLIMYVWHFCTVKRYEFEMHSKVSMAWILGLGPSLGLVRVPGIGFV

YTELASGVPHIFSHFITNLPAIHSVVVFVCVKYLPVYTVPTEERFLVRRIGPRSFHMFRCVARYGYKDLHKRDEDFEKVL

FDCVLFFVRLESMMEGYSDSDEFSVPEQAPGIGRAAFLSVGERTCATVCSSGDLSFSSQDSVVPAAAQSSRAPTGLRVLH

YSASASAAGQGSSGGTVGDELEFLNRCKDAGVVHILGNTIVRARRDSGVVKKLAVDYMYAFMRRVCRENSVIFNVPHESL

LNVGQIFYI

>ZmHAK12

MLMASMSDSETTNRGSMWELDQNLDQPMDEEASQLKNMYKEKKFSSILLLRLAFQSLGVVFGDLGTSPLYVFYNIFPHGV

DEDEDVIGALSLIIYTLTLIPLMKYVFVVLRANDNGQGGTFALYSLLCRHAKVSTIPNQHKTDEELTTYSRQTYEENSLA

AKVKKWLEGHAYKKNCLLILVLIGTCTAIGDGILTPAISVLSAAGGIRVQNQNMSTDVVVIVAVFILIGLFCMQHYGTDK

VGWLFAPLVLLWFILIGSVGLVNIRKYNSSVLKAYNPVYIFRYFRRGKSEIWTSLGGVMLSITGTEALYADLCHFPVLAI

QFAFTLVVFPCLLLAYTGQAAYIIDNKDHVVDAFYRSIPEAIYWPAFIIATLAAVVASQATISATYSIIKQALALGCFPR

VNVVHTSKKFLGQIYIPDINWVLMILCIAVTAGFKNQSQIGNAYGTAVVIVMLVTTFLMVPVMLLVWKSHWILVVIFLVL

SLTVELPYFTACINKVDQGGWVPLVIAITFFVIMHVWHFCTVKRYEFEMHSKVSMAWILGLGPSLGLVRVPGIGFVYTEL

ASGVPHIFSHFVTNLPAIHSVVVFVCVKYLPVYTVPAEERFIMKRIGPKNYHMFRCVARYGYKDIHKKDDNFEKMLLDRL

LIFVRLESMMDGYSDSEDLTVMEHKAKRSTRSLQLIEKAGGNNTMSSTGDLSYSSSQDSIVLAKSPLTGNSLTRYSSQTP

GDELEFLNRSKDAGVVHFLGNTVVQARRDSGILKKVAVNYVYAFLRKMCRENSVIFNVPHESLLNVGQIYYI

>ZmHAK13

MDDVEEGVGRPAVQLQYTTKRAASSPWGLRKATLLLAYQSFGVVYGDLCISPVYVYKNTFSGKLRLHEEDEEILGVLSLV

FWSLTLIPLLKYIILVLGADDDGEGGTFALYSLMCRRSRMGLLMNSINDGCLSVYSQEEEPREEELKSSLAIKSFIERHY

SLRVLLLLFVLMGTSMVIGDGVFTPTMSVLSAVSGLRIKFPELHESKKLFIIHIAHNDLLLLGADASQYFPDYTVLLACF

ILVVLFALQHYGTHRVGFLFAPILLAWLGCIGGIGIYNIFRWNPSVVRALSPYYIYNFFRKAGKDGWSSLGGIVLCITGA

EAMFADLGHFSKLSLRLGFTIVVYPCLVLAYMGEAAYLSKHREDLQSSFYKALPDRVFWPVLIIATLATVVGSQAIISAT

FSIISQSRALGCFPRIKIVHTSSHVHGQIYIPEVNWALMFLCLAVTVGFRDTEMIGNAYGLAVILVMFATTCLMFLVITV

VWSRSVALAALFTAGFGSVELTYLSACLAKVPHGGWLPLLLSLGTLLAMSTWHYGTKRKREHEAQSKVRLDRFLGLSAGM

GLVRVPGVGFVYAASAAAGGVPPVFAHFVTNFPAFHRVLVFVSLQTLAVPRVPPGERFLVGRVGAPAHRMFRCVVRYGYK

EGRRDHFNFENQLLMKVVEFLQLQDAAAAAKAGGCVSGSGELSVIPAHVDAGSAPPSCSEIDAGRRVRFEEPSGAAAGSE

EEVKTLLEELESGVSYMIGHTCVQAHESSPAVKKFAINVVYGFLRRNSRRPAVELGIPNTSLIEVGMTYKI

>ZmHAK14

MKAESGGVVRRSLQKTESAEMRWVVPGGANEDVEIESSDDGSVDTSATASGSRGGGCSDEDEGYENDEMLRQLLVRTGPR

ADSFDVEALDVPGVYRHQEFTLGSSIVLTLQTLGVVFGDVGTSPLYTFDVMFNKYSITAKEDVLGALSLVIYTLILIPFL

KYTLIVLWGNDGGEGGTFALYSLICRNAKASLLPNQLPSDTRIANFNLKVPSVELERSLKIKERLETSSMLKKLLLMLVL

FGTSMVIADGVVTSAMSVMSAVNGLKVGIASVNEGEVVMITVAFLIVLFSLQRFGTSKVGLAVGPALFVWFCCLAGIGMY

NLRVYGPEVLQAFNPVYIYYYFERNPTEAWMSLGGCLLCATGSEAMFADLCYFSVKSVQLTFVFLVLPCLLLGYLGQAAF

LMENLDKSQQVFFLSIPSQAFWPVVFIATLAALIASRAMTTAIFSTIKQATALGCFPRLKIIHTSRKFMGQIYIPVMNWF

LLVSCLAFVAVFGSINEIGNAYGIAELGVMMMTTVLVTIIMLLIWQVNIVIVLCFLNLFLGLELFFFSSVLGSAADGSWV

LLVFAAVLYLVMYIWNYGTKLKYETEVKQKLSMDLLIQLGCNLGTVRAPGIGLLYNELVRGVPAIFGHFLTTLPAMHSMI

IFVCIKWVPVPVVPQNERFLFRRVCPKNYHMFRCIARYGYKDVRKENHQAFEQLLIESLEKFIRREAQERSLERSLESDH

NDDKDSEEEIASSSSRVIDESGGADNSALGSSMSLEEGSSMDNELSFIHKAKESGVVYLLGHGDIRARKESFFVKKLVIN

YFYAFLRRNCRRGIATLSIPHTRLMQVGMQYMV

>ZmHAK15

MAAATGGMRKAPSLEWRWVSTEDDDDEDRAGDGAAAAVVGAVSRGGSFESEDDEDDEEEEDEEKKGRKRLIRTVPSVDWF

DVEGNEVSVAQQLDDSEEFDFGRTMFLALQTLAVVFGDIGIGPLYTFDVMFNKYPILGEEDVLGALSLVLYTLILMPLVK

YVLVVLWANDDGEGGLFALYSLICRNAKVCLIPNQVQSEKRMSSFRLKLPTPELERSIKVKEKLESSLLLKKVLLGLVLF

GTSMFISNGVITPAMSVLSAVSGLKVGLPNTSQDAVVMISIALLVVLFSVQRYATSKVGFAIGPSLLLWFCCLGGIGIYN

LSIYGSTAFRAFNPLYIIYYFGRNPFQAWLSLGGCLLCATGSEAIFSNLCYFPVRYVQYMFVLLVLPCLVLAYLGQAAFL

IANQKSSEHVFFSSIPSGVFWPVFLVANLAALIASRTMTVAIFQCLKQSIALGCFPRLKIVHTSRKFMAKIYIPVVNWFL

LVSCLGFIILFRNIYDVGNAYAIAELGVMIMATVYVTIIMLLIWESNITKVLSFVITFLFLELIFFSSALSSVGDGGWAL

LIFASVLLMIMFIWNYGSKLKYDSEVKQKLPKDLMRKLGPNLGTIRAPGLGLVCSDIVKGVPAIFGHFLTSLPAIHSIIV

FVCIRNVPVPVVPQSERFLFQRVCSRGYHLFRCIARYGYKDKKQEHHSVFERLLIEGLEKFIQREAVELSLQSEDDIDSD

EEPPTPVKIITAPNGSLYSLDVPLLADYVPSTELIHEASCSTPQHDPVLDYAQNLELELAFIEQSKRSGVIYLIDNPIIK

ARKDSWFFKKLMINYFFAFLRNNCRRAIMLMSIPHSNMMQVRMTSYV

>ZmHAK16-a

MAAHQHGTAGRGGGGGSMEIFPYSYSSGRDLELVDLPAEPVDVKRQDSLYRDATMPAHHGHNGQESWVRTLRLAFQCVGI

LYADLGTSPLYVYANTFKYGVHHEDDVLGVLSIIIYSFVLFTMIKIVFIALYANDDGDGGTFALYSLISRYAKVCLIPNQ

QAEDELVSRYKHRSKPSATLRRAQWMKDLLETSKTAKVSLFFLTILATALAISDCMLTPPISVLAAVNGLKLRAPHLTTD

ETVWITVGILVVFFAVQRLGTDKIGYTFAPVVFVWLLLISGIGLYNTVKYDVSTLKAFNAKYIVDYFRRNKKKGWVSLGE

ILLCFTGTEALFADLGYFSIRSIQLSFTFGLLPSVMLTYIGQAAYMRKHIDTLDISNVFFQSIPRSLFWPTFVLALITSV

IGSQAMVSCAFATMSHLQALNCFPRVKILHTSRRYSGQLYSPEVNVFLCIAACVVTHRTLQKSESLIHKYYTCFCYRLCM

HACIRTEICVVLVMVITTLLMTIVMLLVWKVNIWWIAVFFAVFMSTECVYTAAVLYKFTHGPYVPLAMSAVLMFVMAVWH

YVHVKRYRHELERTVSRDAARDLLERGDLKRVPGLGLFYTELVQGIPPIFPHLVGKIPTIHSVVVFITVKSLPVPHVHVT

DRFLFRQVEPKEFMVFRCVARYGYRDALETAADFVSVLVEYLQYYVRDLNLYGVVGDEPFKIVFHSARVDSFSWEKHSSG

HAVYAEEMLTPAQSFSELTMHPVSMSSRLAHFQTGKMNLEEMLKIEEDQKIIQREVDNGVVYIIGESEVVAKPHSNLFKK

IIVNYVYSFLRKNSRNGEKMLSIPRGQLLKVGITYEI

>ZmHAK16-b

MAHTGAAGGPETSLPHSGDLELELPPPVNWLRQDSLYRDATRPAHGAHHHGGQESWVRTLRLAFQCVGIMYADLGTSPLY

VYANTFKYGVHHEDDVLGVLSIIIYSFALFTMVKIVFIALYANDEGDGGTFALYSLISRYAKICLIPNQQDEDELVLRYK

HQAKPSATRRRAQWMKNFLETSKAAKISLFFLTILATALAISDCMLNPPISVLAAVNGLKLKAPHLTKDAEVWITVGILV

ALFSVQRFGTDKIAYTFAPVVTVWLLLIGGIGVYNVIKYDIGTLRAFDPKYIIHYFQRNKKKGWVSLGEILLCVTGTEAL

FADLGYFSIRSIQLSSTFGLLPSVLLTYIGQAAYLRKHMDMDISNAFFNSVPSSLFWPAFILALIASVIGSQAMISCAFA

TMSHLQALNCFPRVKILRTSRHYWGQMYIPEVNIFLCVSACVVTLSFRTTGFIARAHEICVVLVMVITTLLMTIVMLLVW

KVNIWWIAIFFAVFMSTELVYTGAVLYKFVHGPYLSLAMSAVLMAIMIVWHYVLVKRYKYELEHTVSRDKVKGILERQDL

KRVPGLGLIYTELVQGIPPIFPHLIEKIPTVHSVVVFITVKHLPIPHVDVSERFLFRQVEPKHLMVFRCVARYGYLDTLE

MASEFVKILVEYLQYYIRDINLYALGDPLMRVSNCSARIDSFSTEKPSGRHAVYAEEMLTPIQSFSELTMHPVGINSVLT

HLQTAKLNVEEMIKIEEDQKMVEREVDNGVVYILGETEVVAKPHSNLLKKIIVNYVYSFLRKNSRNGEKMLSIPRGQLLK

VGIAYEI

>ZmHAK18

METTSTTNEQTDRGAMWELERNLDQPMDAEAGRLRNMYREKSYPTLVLLQLAFQSLGVVFGDLGTSPLYVFSNIFPHEIE

DTEQIIGALSLVIYSLTLIPLVKYVFIVLRANDNGQGGTFALYSLLCRHAKINTIPNQHRTDEELTTYSRHTYDEKSLAA

KIKRWLEGHQFRKNVILILVLFGTCMAVGDGILTPAISVLSATGGIQVEEKKMKNDAVVIVSVVILIGLFSMQHFGTDKV

SWLFAPIVFVWFILIGVLGAVNISKYDQSVLKAFNPIYVYRYFKRGKTSWASLGGIMLSITGTEALFADLSYFPVQAIQI

AFTVVVFPCLLLQYTGQAAYIAQNKDHVSHAFYFSLPDSVLWPSFIVATAAAIVASQATISMTYSIIKQALALGCFPRVR

IIHTSKKYLGQIYSPDINWILLVFCIAVTAGFKNQSQIANAYGTAVIMVMLVTTFLMIPIMLLVWRSHWTLVILFTVLSL

FVEIPYFTAVVRKIDQGGWVPLVFAVAFLIIMYVWHYGTLKRYEFEMHSKVSMAWILGLGPSLGLVRVPGVGLVYTELAS

GVPHIFSHFITNLPAIHSTLVFVCVKYLPVYTVPLDERFLVKRIGPKNFHMFRCVARYGYKDIHKKDDDFEQMLFDSLML

FVRLESMMEEYTDSDEYSTRETNLSGSANPRINGISAGSNMDLSYTSHDSIIQVQSPNYTGNSQAVSPGQLYHTVGDEIA

FLNACRDAGVVHILGNTVIRARRDSGFIKKIAINYLYAFLRKICRENSAIFNVPHESLLNVGQVFYV

>ZmHAK19

MSLEVEKPPETTKQLKRQDSLYGDAEKVSGFKHHGSEGGWPRLLQLAFQSIGIIYGDVGTSPLYAISSTFPDGIKDQDDL

LGVLSLILYTLILIPMVKYVFIVLYADDNGDGGTFALYSLISRHSKVRLIPNQQAEDAMVSNYGIEAPSPELRVAQWLKQ

KLESSKAAKISLFIITILGTSMVMGDGTLTPSISVLSAVSGIREKVPSLTETQVVWISVPILFALFSVQRYGTDKVGYSF

APIITLWFFLIAGIGMYNLIVHEIGVLRAFNPMYIVDYFRRNGKDGWVSLGGVILCVTGTEGMYADLSHFNIKAIQISFS

TVLLPSVALCYIGQTAYLRKFPESVADTFFRSIPELMFWPTFIIAILSAIIASQAMLSGAFAILSKALSLGCFPSVQVIH

TSKSYEGQVYIPEVNFLMGLASIIVTITFRTTTEIGNAYGICVVTVFSITTHLTTIVMLLVWRKNFIIVLLFYVVFSSIE

LVYLSSILTKFIQGGYLPFCFSLVLMALMITWHYVHVMKYWYELDHVVPANEVTTLLEKHEVRRIPGVGLLYSDLVQGIT

PVFPRLVQRIPSVHAVFLFMSIKHLPIPHVAPVERFLFRQVGPREHRMFRCVARYGYCDKLEESGLFKGFLMESLKTFIQ

DEAAFKTNSTAGDTKELTDPKVSGHDLARWVEKEKQMIDKEMERGVVYLMGEANVIAGPESSAAKKIVVDYVYAFLRKNL

TEGEKVLSIPKDQLLKVGITYEI

>ZmHAK20

MALETDKRNSREAKKVGGNFQRHDSLYGDAERVSDTGYVVSECCGLDTHTYYMRQDSWARTLHLAFQCIGVIYGDIGTSP

LYVYASTFTSGISNVDDLYGVLSLILYSLILLPMIKYVFIVLYANDNGDGGTFALYSLISRYAKVSLIPNQQAEDAMVSN

YALETVSAPMKRAQWTKEMLESSKAAKLAIFLLTVLGTSMVISDGVLTPAISVISAVSGLQQKAPQLKQDQMVWISVAIL

VVLFAVQRFGTDKVGYSFAPIILLWFMFIAGIGIYNLVEYDIGVLRAFYPKYIVDYFRRNGRDAWVSLGGILLCFTGTEA

MFADLGHFNIRSIQLSFSFILFPAVSLAYIGQAAFLRKHPEHVHDTFYKSIPGPMFWPTFIVAVSAAIIASQAMISGSFA

IISQSQTLGCFPRVKVLHTSKLYEGQVYIPEVNFVLGLLCVVVTLAFKTTTNIGNAYGICVTAVMVITTILLAVVMLLIW

RVSIWLIIPFCLVFGSIETVYLSSVLYKFKQGGYLPIVSATVLVTIMGVWHYVHVKKYWYELEHIVTNQEMQELAQAHDI

KRTSGVGFLYTELVQGVPPIFPHLIEKMPFVHSVLVFVSVKHLPIPRVEVAERFRFRRVESRISKMFRCVARYGYIDTVE

GAKEFSASLVEGLQSYIEEGHFMTTVEIEDTEPETTTTSITESHTRTRSSTVHIEEALRPSETTELTQPRMGSSYSAHSS

GRISDEQSRAIAEEKQFVQRELQKGVVYILGETEIWAGPNSSFLKKIVVDYMYSFLRKNFRQGEKAFAIPRQQVLKVGMV

YEI

>ZmHAK21

MVLATAERGGEEKVDHRPAAAAVVDVESGSLYSSSSSVVDRQDSLFREAVTGGHHRGAGGHSHDHDSWGTTLRLAFQCVG

IMYGDLGTSPLYVYSTTFAHGGVGHPDDILGVLSLIIYSFLLFTVVKIVLVALHANDDGDGGTFALYSLISRYAEVSLLP

NHQAEDELVSGYTCTSHGKPPSAALRRAHWLKHLLETSKSAKISLFLLTILAIAMVISDAVLTPPISVLSAVSGLKEKVP

DLTTDQTVWITVAILVLLFAIQRFGTDKVGYSFAPIILLWLLLIGGVGLYNLVKYDAGVLRSFNPKYIVDYFRRNKKDGW

VSLGDILLVFTGTEALFANLGYFSIKSIQFSFSLGLLPSVLLTYIGQAAYLRKHPEHFADTFFRSTPSALFWPTFILAIA

ASIIGSQAMISCAFATVSHLQTLSCFPRVRILHTSKRYHGQLYVPQVNLLLCVAACLVTVSFKTTTIIGKAHVNVYAEIC

VILVMLITTLLMTIVMLLVWRVNVWWIALFFVVFVPTESIYLSSVLYKFTHGPYVPVAMSAVLMAAMVVWHYVHVKRYKY

ELRHTLSPAKAEKLLAEKRDHLKSVPGVGLFYTELVQGVPPIFPHLVDKVPAIHSVLVFVSIKHLHVPHVDASERFLFRQ

VVEPREFRVFRCVARYGYRDSLGDEAQDFVAALLESLQCYVRDVNLYSVHEMQNVSYPVSRDQSLSREKPSGRHAVYAEE

MITPIQSFSELSHGASSNRLPQFQQFAMVHEQASKMNIEELARIEEEQMVIQREAEKGVVYILGETEVVARPQSSLIKKI

AVNYIYSFLRKNFMQGEKMLSIPHGKLLKVGISYEI

>ZmHAK22

MPQQAVLVGLTAGMVAAEGKAAGNKTMMQKQQASSCCSDRDQDMADLELALEAVVPVSVQRQDSLYRDATRAGGGGQQQH

EGWARTLRLAFQCVGVLYGDIGTSPLYVYSSTFAGGIRDTDDLLGVLSLIIYSFLLFTIIKYVYIALRANDDGDGGTLAL

YSLISRHAKVSLVPNHQPEDELQTTDAAAAVLGKHGSVRRRTVQLAASHGREQRAVWVKELLETSKPVRISLFFLTIVAT

AMVISDACLTPAISVLSAVGGLKEKAPNLTTGTAGARPRVRTPDRVDHCGHPGASVRRAALRHPQGGLPVRPRLLLWLLL

IGGVGVYNLLRHDVTVLRAFNPKYILDYFRRNGRDAWVSLGGVLLCFTGTEALFADLGYFSIRSIQLSFGFGLVPAVLLA

YIGQAAFLRRYPEQVSNAFYQSTPESIFWPTFVLALAASVIGSQAMISCAFATISHSQALGCFPRVKVLHTSRQYRGQLY

IPEVNLLLALVACVVTLASKTTAVIAEAHGICVVLVMLITTLLLTLVMLLVWRVNAACVALFFAVFAAAESVYLSSVLYR

FAHGGYIPVAMSAVLVAVMVLWHYVHVKRYKYELERTVSHESVVRDLLPRCRTVPGVGLFYTDLVQGIPPVFPHLVDKIP

SIHAVLLFVSVKHLPVPHVDATERFLFRQVASSSESDTAAGPRVFRCVARYGYRDLLEEASDFAGSLVERLQYYIRDVNL

YGVEHQQPGAKVSYPSSRCDSMILMARQRSSVMMLRQSSAASYYSSYAAAAETQQLQLARARSTSGAGGILLHSASERAE

QLARARSTGIFAEEMLTPAESFSELSRMGSIGGGGMQAVKISLEEMARIEEEQRFIEREMEKGVVYILGESEVVARPHSS

LLKKLLVNYAYAFLRNNCRQGEKMLAIPKSQLLKVGMSYEI

>ZmHAK23

MDDGGIQEEPPSTRFLTPTRSGGTRWVDGSEVDSSESTPSWSLEDERSAGGVSSNGGAAAASRVSSGAFRRRFGKRPRRV

DSLDVESMNVRGAHGHSSKEISMLSTLAMAFQTLGVVYGDMGTSPLYVFSDVFSKVPIKSEVEILGALSLVMYTIALIPF

AKYVFIVLKANDNGEGGTFALYSLICRYAKVSLLPNQQRVDEDISSFRLKLPTPELERAISVKDCLEKKPLFKNILLFLV

LMGTSMVIGDGILTPSMSVMSAVSGLQGQVPGFDTNAVVIVSIVVLLLLFSVQRFGTGKVGFMFAPILALWFINLGSIGI

YNLVKYDISVVRAFNPVYIYLFFETNGIKAWSALGGCVLCITGAEAMFADLGHFSVKSIQVAFTAVVFPCLLIAYMGQAA

FLMKNPLVVERIFYDSVPGVLFWPVFVIATLAAMIASQAMISATFSCIKQAMALGCFPRIKIIHTSKKVMGQIYIPVMNW

FLMVMCIIIVATFRSTNDIANAYGIAEVGVMMVSTALVTLVMLLIWQTNLFLVLCFPILFGAVEFVYLTAVLSKIQEGGW

LPLAFSSLFLCIMYTWNYGSVLKYQSEMRGKISLDFILDLGATLGTVRVPGIGLVYNELVQGIPSIFGQLLVTLPAMHST

IVFVCIKYVPVPYVPLEERFLFRRVGQKDYHMFRSVARYGYKDVRKEDHGFFEQLLVESLEKFLRREAQEIALEASTMEA

ERDDISVVSEVPQTPAGDGDLQTPLLSDQRSGDNNRMAATDGSDPVLPSSSMSLEEDPGLEYELSALREAMASGFTYLLA

HGDVRARKESVFTKKFVINYFYAFLRRNCRAGTATLKVPHSNIMRVGMTYMV

>ZmHAK24

MDAESGRGAAGARKKSWRSELVLAYQSLGVVYGDLATSPLYVYKSAFAGGDIEHSEGNEEIYGVLSLVFWTLTLITLLKY

VLVVLRAADDGEGGTFALYSLICRRVRAGLLPGVGDSAVDELKDQPDGALPPPASSVRAALQQRRELQWLLLLFALLGTS

MVIGDGVLTPAVSVFSAVSGLKLSMVNEQHQYVLLPVTCVILVGLFALQHFGTHRVGFLFAPIVCLWLLCISTIGVYNIF

IWNPHIYKALSPYYMYRFLQKTQVGGWMSLGGILLCVTGSEAMYADLGHFSQSSIKIAFTAVVYPSLVLAYMGQAAYISR

HHNFERNHHIGFYISVPEKIRWPILGIAILAAVVGSQAVITGTFSVIKQCCSLNCFPRVKIVHTSSTVHGQIYIPEINWI

LMILCLAVTIGFRNTKQMANAQGLAVITVMIVTTCFMSLVIVLCWNKNVVFALAFLLFFGAIEAVYFSASLMKFHEGAWV

PIIVSFIFLTVMCVWHYGTAKKYEFDVENKVSISWLLNLGSSLGIVRVRGIGLIHTELVSGIPAIFSHFVTNLPAFHQVL

VFLCIKSVSVPHVQPEERFLVGRIGLKQYRLYRVVVRYGYRDVQQDSLQFEKALVSSIAEFIRSGDSDQNGYPDGSESPY

ERLSIISKGLPFQEADGDGSPSPESSARKDTNPILVSSKSRRVRFVLPENAQINSQVRNELQELTEAREAGMSFIMGRSY

MKAKSGSSLMKRIAINFIYEFLTRNSRGPAYAANVPHVSTLEVGMVCQV

>ZmHAK25

MDLESADGAEAADGKRRGESLRATLLLAYQSLGVVYGDVATSPLYVYKSAFAGNDIQHSEGNEEIYGVLSFVFWTLTLIT

LIKYVLIVLRADDGGEGGTFALYSLICRHVRAGLLPGGGTRDELMEEEKVTGRRGERPVSRVRAVLEKYRVLQRLLLLFA

LLGTCMVIGDGVLTPAVSVFSAVSGLELSLEKEQHKYIELPVACAILICLFALQHYGTHKVGFLFAPIVCIWLVCISAIG

LYNIIRWDPHVYRALSPYYMYQFLRKTQTGGWMSLGGILLCVTGSEAMYADLGHFSQSSIQIAFISVVYPALVLAYMGQA

AFISQHHSFESSYHIGFYVSVPETLRWPVLVIAILAAVVGSQAIITGTFSIIKQCSSLSCFPGVKIVHTSSTLHGQIYIP

EINWMLMILCLAVTIGFRDTKHLANAQGLAVITVMLVTTCLMSLVIVLCWNKSIFLALGFLLFFGTIEVIYFSASLVKFH

EGAWVPISLSFIFMVIMSVWHYGTIKKYEFDVQNKVSVNWLLNLGPSLGIVRVRGIGLIHTELMSGIPAIFSHFVTNLPA

FHQVLVFLCVKSVPVPHVQPEERFLVGRIGPKEYRLYRVIVRYGYRDVQKDDLEFEKELVSNIAEFIRSSGEYDKNGFVE

DADKPFEKLSTISTGINMLEEDGEVDAHVSPHKEIDPHNAAPKRKKARFMIPKSAQVDSEVRRELQELMDAREAGMSFIL

GHSYMKAKSGSSFIKRVVINFFYEFLRKNSRGPAYAANIPHASTLEVGMVYQV

>ZmHAK26

MEHRAAAPDVIVQVNSAAVAAVDERISTCQITEEEGGIVGHEQVPVGCLARRTFSQSYRTRPRNPLEFTGWQLALLAYQS

LGVVYGDIGTSPLYTFSSFALPDPGTADILGILSLILWTLTLVSLVKYVFIVLHADDHGEGGTFALYSLLRQHVNFSGKS

VPVPVTRLASDANLRFHSRKSSQQPRMLEFLEGSAMAQAVITYLVLVGTCMVMGDGALTPSISVLSAVQGIQSRSSSIKQ

GHVVLLCVVILVILFLFQQYGTSKVGFTFSPIMLVWFALIASTGLYNIIKHYPPVLKAISPHYIYLFFARNKRAGWEQFG

TVVLCITGAEAMFADLGHFNKKSIQMAYSCLVYPSLILAYAGQAAFLIKNPSKLSTTFYSSVPEPLFWPVFVVATLAAIV

ASQALISASFSIVRQSVALGCFPRVTMKHTSKKYEGRVYSPEINYFLMVACILITVGFKGGPEIGQAYGVAVIWVMLITT

HLITVVMVIIWQLHSAIAWSFYVIFAAIEGLMTISLLYKIAQGGWVPFAITAFFLIITLSWTYGRSKKQEYEVSNLMDRQ

EFIKTVNTSNRVPGICIFCTDLMNGIPPIVRHYVEHMGCLRELMVFVTVRHLPVTSVLPEERFLFDRLEPFGVYRCIVQY

GYMDTQNMEDDEYVVSIVASLKEIAQSGAEILMMDSALANGTTFVLGRVILNMSPQRNNCFKRFVINNLYRFLQKNFRSN

ISSLKIAPSKTLQVGMQYEI

>ZmHAK27

MAPAQHQAAADRGSREEIVIVDLESEAGADDAAAAAATMQRQDSLYAAATRAAGANHHGQDSWARTLRLAFQCVGILYGD

IGTSPLFVYSSTFRDGVGHPDDLLGALSLIIYSFLLFTVIKYVYIALRANDDGDGGTFALYTLISRHAKVSLIPNQQVEE

DDLVAKYNRDKPPATLRRAEWMKELLETNKAVKVSLFLITMLATAMVISDAILTPAISVLSAVGGLKEKATFLTTDEIVW

ITVGILVALFAIQRFGTDKVGYLFAPIILLWLLLIAAVGFYNLVKYDTGALRAFNMKYIIDYFRRNKKKGWASLGGILLC

FTGTEALFADLGYFSIRSIQLSFGFGLVPSVLLAYIGQAAYLRVHPEDVANTFYRSTPISLFWPTFILALAASIIGSQAM

ISCAFATISHSQTLGCFPRVKILHTSRQYSGQLYIPEVNYLLCLGACLVTVGFRTTVIIGEAHGICVVLVMIVTTLLLTI

VMLLVWKISVWWIVAFFLVFMSSESIYLSAILSRFAHGAYVPVAMSAVLMVVMVVWHYVHVNKYRFELEHSIPRDKVKEL

LERSDIQRVPGIGLFYTELVQGIPPVFRHLIEKIPSIHSVLIFVSMKHLPIPSVDMSERFLFRQVDREDYKVFQCVARYG

YRDPFEEAKDFVDKLVEHLQYYIRDVNLYGLGCEPMMKQSFSYCSSRAESFGSHEKTKVKAVYAEEMLTPAESFSEHARQ

ASGKNKLFTQFQGDKMNIVEMMKIQQEQQAVLEEMSKGVVYIFGESEVVARPHSSLIKKIAINYIYSFLRKNSRNGEKML

SIPRRQILKVGVSYEI
